# Depression among healthcare workers in the Eastern Mediterranean Region: a systematic review and meta-analysis

**DOI:** 10.1186/s12960-021-00628-6

**Published:** 2021-07-10

**Authors:** Amit Abraham, Karima Chaabna, Sathyanarayanan Doraiswamy, Sapna Bhagat, Javaid Sheikh, Ravinder Mamtani, Sohaila Cheema

**Affiliations:** 1 Institute for Population Health, Weill Cornell Medicine-Qatar, Education City, Qatar Foundation, P.O. Box 24144, Doha, Qatar; 2 Office of the Dean, Weill Cornell Medicine-Qatar, Doha, Qatar

**Keywords:** Depression, Healthcare workers, Eastern Mediterranean Region, Epidemiology

## Abstract

**Background:**

Depression is a major population health challenge globally. This systematic review and meta-analysis aims to (i) determine depression prevalence and (ii) identify the risk and protective factors of depression among healthcare workers (HCWs) in the Eastern Mediterranean Region (EMR).

**Methods:**

The protocol was registered on Open Science Framework (registration ID: https://osf.io/rdv27). We searched five databases (PubMed, Embase, PsycINFO, Al Manhal, Google Scholar) till July 22, 2020 without language restrictions. We included studies from the EMR using a depression screening or diagnostic instrument to measure the depression prevalence among HCWs. Studies were assessed and data were pooled using random-effects meta-analysis based on the Cochrane handbook.

**Results:**

The systematic review identified 108 studies from 12 EMR countries with varying quality. Working long hours, poor sleep quality and being female were risk factors for depression in EMR HCWs. The meta-analysis comprised 77 studies providing 122 prevalence measures across 7 EMR countries. The pooled prevalence of depression among EMR HCWs was 33.03% (95% CI = 27.40–39.19%). Emergency HCWs had markedly higher rates of depression [53.14% (95% CI = 26.63–77.99%)] compared to HCWs of other specialties. Most studies had an appropriate sample size.

**Conclusions:**

Depression among EMR HCWs is a major concern. Steps must be taken to prevent, identify, and manage depression among HCWs. Fostering a compassionate and empathetic environment is critically important to building a resilient healthcare system. Generating high-quality regional data from longitudinal studies on mental health will further contribute to a better understanding and management of depression among EMR HCWs.

**Supplementary Information:**

The online version contains supplementary material available at 10.1186/s12960-021-00628-6.

## Background

Depression is one of the most urgent yet underappreciated population health challenges globally. It was the leading contributor to years of life lost due to disability in 2015 [1]. An analysis of the Global Burden of Disease data demonstrates an increase of 49.86% in the global burden of depression from 1990 to 2017 [2].

Healthcare workers (HCWs) are one of the high-risk groups for adverse mental health outcomes worldwide. Published literature establishes that HCWs are susceptible to alarming levels of psychological distress [3], anxiety [4], emotional exhaustion and burnout [5].This is especially relevant for depression—its prevalence among HCWs ranges from 21.53% to 32.77% in high-income nations, much higher than that of the general population worldwide (4.40% in 2015) [1, 6–9]. HCWs are subject to exceedingly high levels of academic and professional stress and must manage workplace stressors in addition to stressors in their personal lives [6, 10–12]. Thus, not only are HCWs negatively impacted by sustained exposure to high pressure, but the quality of care they provide to patients and patient safety may also be compromised. This may result in patient dissatisfaction, high HCW turnover rates, medical errors, and associated financial costs [6, 10–12]. Increased psychological morbidity in HCWs due to long working hours delivering care to critically ill patients is well described. What we are currently experiencing with the ongoing COVID-19 pandemic is testament to this. The resilience of HCWs in the era of COVID-19 has been further eroded due to the loss of social and familial support secondary to population health measures, such as physical distancing [13–15].

The World Health Organization’s Eastern Mediterranean Region (EMR) comprises 21 member states and Palestine in the Middle East and North Africa and has a population size of nearly 583 million [16]. There is limited information describing the overall burden of depression in HCWs within the EMR. The EMR countries are inundated with myriad challenges that adversely affect mental well-being, including political instability and conflict, healthcare disparities and HCW shortages, stigma, and a lack of investment in mental health services [17, 18]. These challenges can have repercussions on the already limited healthcare workforce and may exacerbate the HCW shortage. One EMR country, Iran, reported a prevalence of depression among HCWs ranging from 22.00% to 45.30% in four meta-analyses published between 2017 and 2020 [19–22]. High depression prevalence among HCWs similar to Iran is also likely to be found across the EMR region due to similarities in culture, traditions, and customs [23]. We thus aimed to conduct a systematic review and meta-analysis to describe the epidemiology of depression among HCWs in the EMR. The objectives of our study are to (i) estimate the prevalence of depression and (ii) identify the risk and protective factors that may predispose to or protect HCWs in the EMR from developing depression.

## Methods

### Overview

We conducted a systematic review and meta-analysis of primary studies on depression among HCWs in the World Health Organization’s EMR, based on the Cochrane Handbook for Systematic Reviews [24]. The protocol was registered on the Open Science Framework [25] (registration ID: https://osf.io/rdv27). The systematic review and meta-analysis are reported using the Preferred Reporting Items for Systematic Reviews and Meta-Analyses (PRISMA) guidelines (Additional file 1: Additional material 1) [26] and the PRISMA for Abstracts Checklist (Additional file 1: Additional material 2) [27]. This systematic review and meta-analysis is a segment of a research project that aims to synthesize scholarly literature on population health issues in the EMR [18, 28–36].

### Eligibility criteria

The eligibility criteria were established a priori*,* as defined in the protocol [25]. All measures of depression and depressive symptoms were considered if an instrument (whether diagnostic or screening; validated or not) had been used to quantify it, or if a trained mental health professional made the diagnosis. If the study used a diagnostic test, we reported the findings as depression. If the study reported dysthymia following the criteria set out in the Diagnostic and Statistical Manual of Mental Disorders, it was reported accordingly. If a screening instrument was used in the study, we reported results as depressive symptoms, because screening instruments are not designed to diagnose depression. Studies with data on point and lifetime prevalence, as well as risk and protective factors, were included in this systematic review and meta-analysis.

In addition to Palestine, the 21 countries from EMR included in our study are Afghanistan, Bahrain, Djibouti, Egypt, Iran, Iraq, Jordan, Kuwait, Lebanon, Libya, Morocco, Oman, Pakistan, Qatar, Saudi Arabia, Somalia, Sudan, Syria, Tunisia, United Arab Emirates (UAE), and Yemen [16]. We included cross-sectional, cohort, and case control studies that had data on any type of HCWs. Any publication type, including published literature, theses and dissertations, and conference proceedings were considered eligible. Preprints identified prior to July 22, 2020 were also included to potentially characterize depression epidemiology during the COVID-19 pandemic.

We excluded studies that lacked data on depression but presented data on related mental health illnesses, such as burnout or anxiety; interventional and experimental studies; as well as qualitative studies. Systematic reviews were also excluded. However, all identified primary studies from any systematic review that met the eligibility criteria were included. Any study reporting data on solely hospital administrative workers was excluded, as were studies that assessed depression by merely asking participants if they were depressed or not. No language or date restrictions were applied.

### Search strategy

To identify relevant primary studies, two authors (AA & KC) systematically searched Medline (via PubMed), Embase, and PsycINFO from inception until October 16, 2019. A combination of keywords related to EMR countries and regions (such as ‘Middle East’, ‘North Africa’, ‘Persian Gulf’, etc.) or population descriptor (such as ‘Arab’, Bedouin’ etc.), HCWs, and depression were used (Additional file 1: Additional material 3). The search terms were modified appropriately for the other databases. To ensure comprehensiveness, we searched for gray and non-gray literature in Google Scholar and Al Manhal. Al Manhal is an electronic database that provides scholarly and grey literature on the Middle East and North Africa in English, Arabic, and French languages [37]. An update of the search was completed using Google Scholar Alerts on July 22, 2020. We also systematically checked the bibliographies of relevant included studies for additional references.

### Study selection

Using the online systematic review software, Rayyan [38], AA removed all identified duplicates, following which AA and SB independently conducted a multistage screening process (title/abstract screening and full-text screening). Studies in French and Arabic were independently screened by KC and SC1. Discrepancies at both stages were reconciled through discussion with a third reviewer (KC) under the supervision of the senior authors (SD, SC, and RM). The reasons for exclusion at each step were recorded.

### Data extraction

Data extraction was independently conducted by at least two reviewers (divided between AA, KC, SB, and SC1). An iterative process was used to develop a standardized table to extract relevant information, after piloting on a small study sample. A summary of information extracted from each study includes: (i) study methodology; (ii) country; (iii) setting; (iv) participant demographics; (v) sample size and methodology; (vi) time the study was conducted; (vii) outcomes of interest; (viii) instrument and diagnosis cutoff; (ix) comorbidities; (x) risk and protective factors; (xi) recommendations, (xii) limitations; and (xiii) funding and conflicts of interest. If any retrieved article was in a language unknown to the authors, data were extracted from the abstract and the corresponding (excluded) systematic review, if applicable. In the case of multiple publications from the same study, the more comprehensive study was prioritized. A consensus meeting was held between AA, KC, SC1, and SB to resolve any disagreement, under supervision of the senior authors (SD, SC, and RM).

### Quality assessment

The methodological quality of the primary studies was assessed by a single reviewer (divided between AA, KC, and SB) using an a priori checklist adapted from the Cochrane Risk of Bias [39] and PICOTS framework [40]. Studies were classified as having low or high risk of bias depending on (i) sampling technique (probability versus non-probability based); (ii) sample size (≥ 100 versus < 100); (iii) adequate description of study participants (age, sex, and profession); (iv) setting (hospital or healthcare center setting); and (v) instrument utilized to diagnose depression (use of a validated screening/diagnostic instrument versus a de novo instrument). The minimum sample size was calculated based on the pooled prevalence of depression after meta-analysis. For a depression prevalence of 33.03% and a sample size of 100, the 95% confidence interval (CI) was calculated as 23.9–43.1% (binomial method calculation) [41]—a reasonable 95% CI estimate for perceived depression prevalence measure. Studies that did not provide information regarding any of the above-mentioned criteria were designated as having an unclear risk of bias.

### Qualitative synthesis

We narratively synthesized the findings from all the included studies. The characteristics of the primary studies synthesized are provided in Additional file 1: Additional material 4. Additional file 1: Additional material 5 lists the risk and protective factors, as well as the recommendations and limitations noted by the authors of the primary studies. The list of the excluded studies at the end of full text screening is provided in Additional file 1: Additional material 6.

### Quantitative synthesis

We conducted a meta-analysis based on the random-effects model to compute pooled point prevalence estimates and their 95% CI. Forest plots were generated. Pooling was done with the ‘PLOGIT’ transformation method, which used the logit transformation of the proportion. To calculate pooled prevalence for the EMR region, we examined all studies eligible for our meta-analysis. Studies eligible for our meta-analysis reported a sample size of more than 20 and provided a prevalence of depression alone (not combined with other mental health illnesses). The heterogeneity between studies was assessed using the I^2^ statistic, Q test of heterogeneity, and prediction interval. The heterogeneity was considered as insignificant when the Q test’s *p* value was higher than 0.10 and I^2^ < 50%.

To explore the heterogeneity between studies, we conducted a sub-group analysis according to period of publication. We categorized the data as those published before 2011, between 2011 and 2019, and in 2020. We analyzed 2020 separately as a measure of the potential excess strain placed on HCWs due to the ongoing COVID-19 pandemic. In addition, the pooled prevalence was calculated by EMR country, sex, profession [doctors, resident physicians, nurses, Emergency Medical Technicians (EMTs) and allied healthcare (all other professions)], and specialty. For professions, an additional category for ‘all HCWs’ was used if the primary study failed to specify profession and specialty. Resident physicians are known to be exposed to heavy workload and stress, resulting in elevated depression risk [6, 42]. We wanted to determine whether EMR resident physicians suffered different rates of depression compared to physicians who had completed their residency training and thus considered them as a separate category. Pooled prevalence was also estimated by the type of instrument to determine whether variability between studies potentially arose due to the instrument used to ascertain the diagnosis of depression among study participants. The meta-analysis was conducted using R software (version 4.00) and its ‘meta’ package.

## Results

### Characteristics of the primary studies

**Fig. 1:**
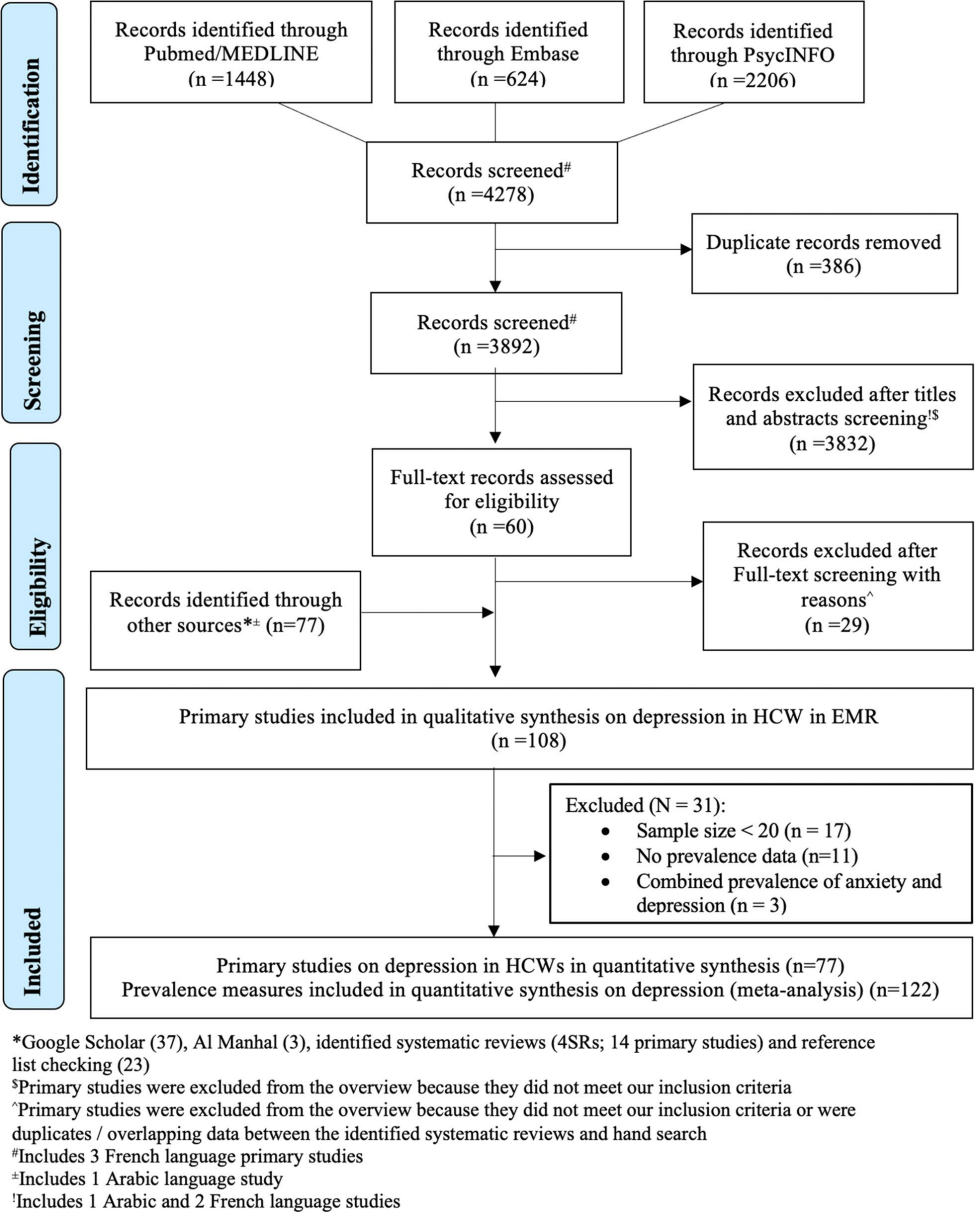
PRISMA 2009 flowchart depicting literature search

The main and the supplementary search strategies identified 108 primary studies. Ninety-seven of these primary studies provided 555 prevalence measures relevant to the epidemiology of depression amongst HCWs across 12 EMR countries (Bahrain, Egypt, Iran, Iraq, Jordan, Lebanon, Oman, Pakistan, Saudi Arabia, Sudan, Tunisia, and the UAE) (Additional file 1: Additional material 5). The remaining 11 primary studies exclusively described the risk and/or protective factors of depression among EMR HCWs. After excluding the studies that had a sample size of less than 20, 77 primary studies providing 122 prevalence measures were included for the meta-analysis, comprising 26,029 HCWs across seven countries (Fig. 1).

Most of the primary studies (75/108) included both male and female HCWs. The HCWs identified in the studies comprised physicians, nurses, pharmacists, dentists, EMTs, midwives, allied health staff, and unspecified HCWs. Most studies focused on nurses (49/108). The medical specialties reported in the studies include internal medicine, surgery, pediatrics, obstetrics and gynecology, radiology, family medicine, psychiatry, intensive and critical care medicine, anesthesia, and emergency medicine. Except for one longitudinal cohort study [43], all primary studies included in our systematic review adopted a cross-sectional design. Primary studies were published between 2005 and 2020. All primary studies reported point prevalence of depression, with the Beck Depression Inventory being the most frequently used instrument to assess depressive symptoms.

There were seven studies [44–50] that assessed depression among HCWs in the context of COVID-19, with data from five EMR countries: Iran, Jordan, Oman, Pakistan, and Saudi Arabia. Of these, three studies were peer-reviewed manuscripts [45–47], while four were preprints [44, 48–50].

### Methodological quality assessment of primary studies

Out of 108 primary studies, 70 (64.81%) appropriately described the study participants. Except for three primary studies [48, 49, 51], all others clearly described the study setting. Eighty-two out of 108 studies (75.92%) had an adequate sample size and less than half (46.29%) used probability-based sampling methods. Only one primary study [52] used a non-validated screening or diagnostic instrument. The quality assessment of the primary studies is described in Additional file 1: Additional material 7.

We recorded the conflict of interest and funding declarations from all the primary studies included in our systematic review and meta-analysis. Forty-six primary studies disclosed no conflict of interest, 37 studies failed to provide a conflict of interest declaration, and two studies reported a conflict of interest non-relevant to the study context. Conflict of interest and funding source was unclear for 23 studies due to non-availability of full text or due to the language of publication (full text in Persian). Forty-eight studies failed to provide funding sources, 25 studies disclosed no funding sources and 12 studies disclosed funding sources (university, hospital, or governmental grants) (Additional file 1: Additional material 4).

### Epidemiology of depression among HCWs in the EMR

In the EMR, both the highest and lowest depression prevalence occurred in Iran, based on the findings of the systematic review. The overall depression prevalence ranged from 0.90% among nurses working in critical care units and emergency rooms to 99.90% among medical staff (specific profession and specialty not reported). The pooled prevalence of depression from Egypt, Iran, Lebanon, Oman, Pakistan, Saudi Arabia, and the UAE among all HCWs was 33.03% (95% CI = 27.40–39.19%) (Table 1). Only point prevalence (and no lifetime prevalence) data was identified.

**Table 1:** Meta-analysis of depression among HCWs in the EMR

Studied subgroups	Years of data collection	Number of prevalence measures	Total sample size	Prevalence range (%)	Effect size	Subgroup Comparison	Heterogeneity between studies			
					Weighted average prevalence (%)	95% CI	Q between subgroup tests *p* value	Cochrane Q test's *p* value	I^2^ (%)	95% prediction interval^a^
EMR										
All countries (Egypt, Iran, Lebanon, Oman, Pakistan, Saudi Arabia and the United Arab Emirates)	2005–2020	94	26,029	2.40–99.90	33.03	27.40–39.19	N/A	< 0.0001	98.80	3.53–86.94
By period										
< 2011	2005–2010	23	5810	2.40–81.81	25.80	17.84–35.76	0.3860	< 0.0001	98.00	3.05–79.34
2011–2019	2011–2019	38	10,682	2.51–92.90	33.87	25.05–43.99		< 0.0001	98.90	3.26–88.61
2020	2020	10	3363	24.00–99.90	46.12	21.30–73.02		< 0.0001	99.50	1.02–98.61
Sex										
Females	2012–2017	5	1285	2.90–53.80	23.35	8.24–50.82	0.1154	< 0.0001	97.30	8.24–50.82
Males	2017–2018	3	310	26.70–57.10	53.43	27.43–77.69		< 0.0001	90.60	0.00–100.00
Profession										
Health Care Workers	2010–2020	12	3814	25.00–99.90	55.22	33.14–75.41	0.2261	< 0.0001	99.40	3.03–97.99
Doctors	2008–2020	17	3368	6.10–85.30	36.27	24.70–49.69		< 0.0001	97.90	4.46–87.42
Residents	2012–2020	26	7003	1.70–81.81	32.78	22.08–45.63		< 0.0001	98.80	2.66–89.68
Nurses	2005–2020	49	11,942	2.40–92.90	26.87	20.67–34.12		< 0.0001	98.30	3.07–80.97
EMTs	2017–2019	2	344	26.70–41.00	32.89	23.63–43.71		0.0073	72.00	N/A
Allied Health Staff	2008–2020	3	428	7.80–63.20	26.39	7.97–59.74		< 0.0001	96.80	0.00–100.00
Specialty										
Internal Medicine	2013–2017	3	419	3.00–46.00	20.54	4.42–59.10	0.0001	< 0.0001	96.40	0.00–100.00
Surgery	2013–2017	3	178	1.70–70.60	12.66	1.16–64.11		< 0.0001	92.70	0.00–100.00
Obstetrics & Gynecology	2013	1	20	12.00	10.00	2.51–32.38		N/A	N/A	N/A
Family Medicine	2014–2016	2	279	20.00–22.00	20.43	16.10–25.57		0.7560	0.00	N/A
Emergency Medicine	2014–2016	3	403	31.40–81.81	53.14	26.63–77.99		< 0.0001	96.00	0.00–100.00
Radiology	2017	1	45	2.20	2.22	0.31–14.16		N/A	N/A	N/A

^a^Only calculated when 3 or more data points are available

*EMT* Emergency Medical Technician

### Sensitivity analysis of depression prevalence by instrument

Seventeen different instruments were used to measure depression and dysthymia in the primary studies included in the systematic review, only four of which were diagnostic instruments: Diagnostic and Statistical Manual of Mental Disorders-IV (DSM-IV), WHO Composite International Diagnostic Interview (CIDI), Minnesota Multiphasic Personality Inventory-2 (MMPI-2), and the Present State Examination/Schedules for Clinical Assessment in Neuropsychiatry (PSE-10/SCAN).

The depression prevalence range reported in the primary studies that used diagnostic instruments was 2.90–81.81%, while the range in primary studies using screening instruments varied from 2.40% for the Depression Anxiety and Stress Scale-42 (DASS-42) to 99.90% for the Depression Anxiety and Stress Scale-21 (DASS-21) (Table 2). The pooled depression prevalence across all diagnostic instruments was 26.31% (95% CI = 6.29–65.50%) as compared to 33.37% (95% CI = 27.74–39.53%) for the screening instruments (*p* = 0.6949). There was no statistical difference between the pooled prevalence from the diagnostic and screening instruments, indicating that there is probably no added heterogeneity due to the instruments.

**Table 2:** Sensitivity analysis of instrument

Studied subgroups	Years of data collection	Number of prevalence measures	Total sample size	Prevalence range (%)	Effect size	Subgroup comparison	Heterogeneity between studies			
					Weighted average prevalence (%)	95% CI	Q between subgroup tests *p* value	Cochrane Q test's *p* value	I^2^ (%)	95% prediction interval^a^
EMR										
All countries (Egypt, Iran, Lebanon, Oman, Pakistan, Saudi Arabia and the United Arab Emirates)	2005–2020	94	26,029	2.40–99.90	33.03	27.40–39.19	N/A	< 0.0001	98.80	3.53–86.94
Diagnostic Instruments^b^	2012–2015	5	1192	2.90–81.81	26.31	6.29–65.50	0.6949	< 0.0001	99.10	0.05–99.61
Screening Instruments	2005–2020	89	24,837	2.40–99.90	33.37	27.74–39.53		< 0.0001	98.80	3.87–86.16
BDI	2005–2018	25	6679	7.80–89.25	33.33	24.91–42.97	< 0.0001	< 0.0001	97.90	5.43–81.31
BDI–2	2006–2018	8	1082	22.00–92.90	45.90	26.88–66.18		< 0.0001	97.20	3.80–94.80
BDI–SF	2017	1	247	9.88	9.72	6.60–14.09		N/A	N/A	N/A
DASS–21	2010–2020	18	4903	9.50–99.90	40.21	26.04–56.22		< 0.0001	99.00	3.22–93.15
DASS–42	2007–2016	2	470	2.40–29.10	9.23	1.36–42.79		N/A	95.40	N/A
HADS	2012–2020	14	4412	6.10–82.00	26.05	15.98–39.47		< 0.0001	98.30	2.53–82.72
PHQ–9	2013–2020	10	4332	2.51–71.4	33.89	18.20–54.15		< 0.0001	99.20	2.01–92.77
Other^c^	2006–2016	11	2712	6.20–85.10	33.45	18.47–52.73		< 0.0001	98.30	2.15–92.00

*BDI* Beck Depression Inventory, *BDI-2* Beck Depression Inventory-2, *BDI-SF* Beck Depression Inventory-Short Form, *DASS-21* Depression Anxiety and Stress Scale-21, *DASS-42* Depression Anxiety and Stress Scale-42, *HADS* Hospital Anxiety and Depression Scale, *PHQ-9* Patient Health Questionnaire-9

^a^Only calculated when 3 or more data points available

^b^Includes: Composite International Diagnostic Interview (CIDI); Diagnostic and Statistical Manual of Mental Disorders-IV (DSM-IV); Minnesota Multiphasic Personality Inventory-2 (MMPI-2); Schedules for Clinical Assessment in Neuropsychiatry (WHO SCAN); and clinical diagnosis by doctors

^c^Other: General Health Questionnaire-28 (GHQ-28), Center for Epidemiologic Studies Depression Scale (CES-D), Hamilton Depression Rating Scale (HAM-D), Zung Self-Rating Depression Scale, Symptom Checklist for Depression, de novo questionnaire

In addition, the pooled prevalence computed for all HCWs and EMR countries was significantly different between the screening instruments, varying between 9.23% for DASS-42 to 45.90% for BDI-2 (*p* < 0.0001). See Additional file 1: Additional material 8 for further information about the instruments used.

### Geographical pattern of depression prevalence

**Fig. 2:**
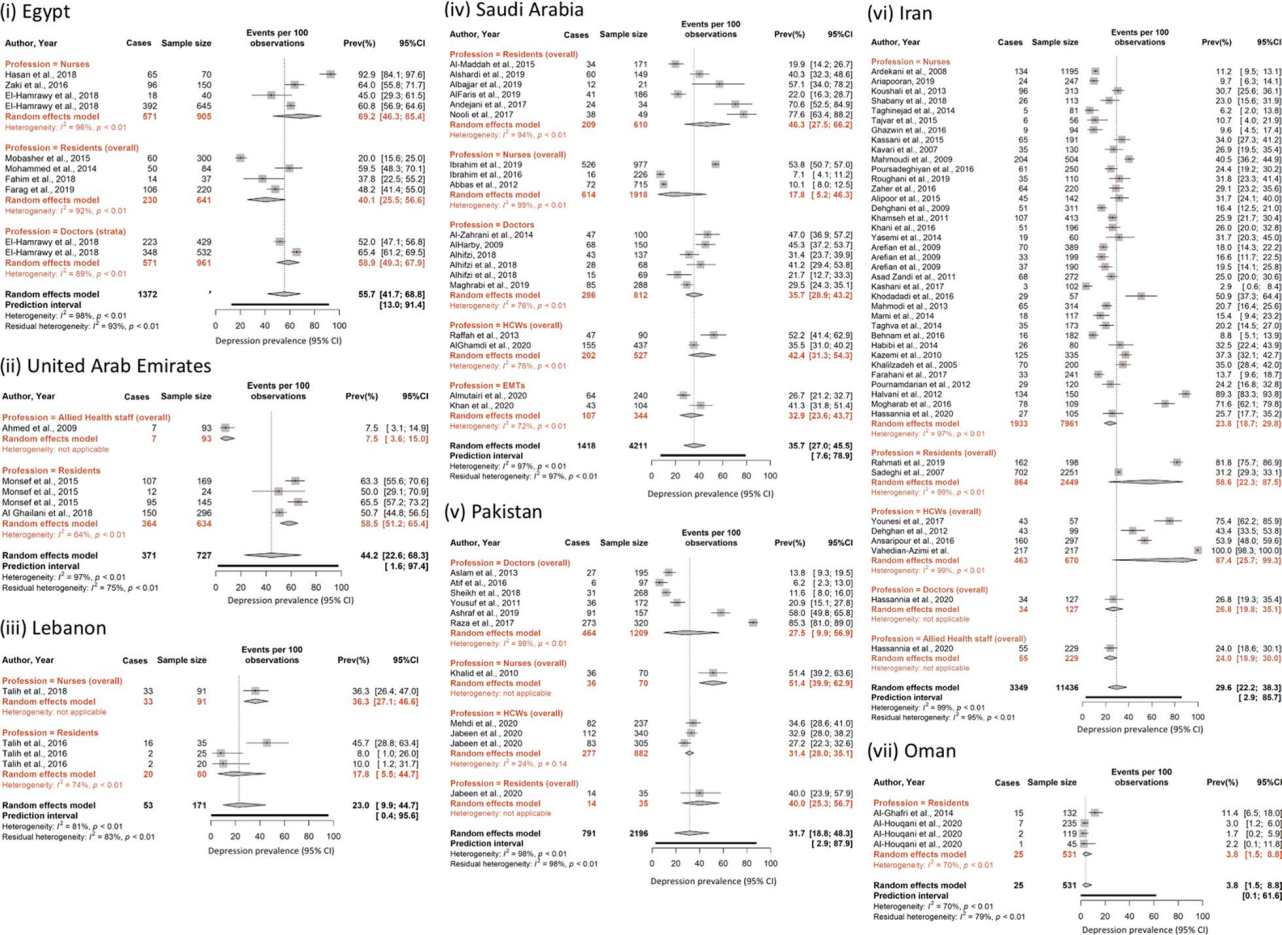
Forest plots with pooled prevalence of depression in EMR HCWs by country

Among the EMR countries, Iran reported both the highest [99.90% among male and female medical staff (specific profession and specialty not reported)] and lowest (0.90% among male and female nurses working in critical care units and emergency rooms) depression prevalence, as described earlier. Nurses (specialty not specified) in Iraq self-reported the second lowest depression prevalence (2.40%), while nurses working in a psychiatric hospital in Egypt had the second-highest prevalence (92.90%). The lowest pooled prevalence of depression among all professions was found in Oman [3.75% (95% CI = 1.54–8.84%)], and the highest in Egypt [55.69% (95% CI = 41.74–68.79%), Table 3 and Fig. 2a–c].

**Table 3:** Meta-analysis of depression among HCWs in the EMR by country and profession

Studied subgroups	Years of data collection	Number of prevalence measures	Total sample size	Prevalence range (%)	Effect size	Subgroup Comparison			Heterogeneity between studies	
					Weighted average prevalence (%)	95% CI	Q between subgroup tests p value	Cochrane Q test's p value	I2 (%)	95% prediction intervala
EMR										
All countries (Egypt, Iran, Lebanon, Oman, Pakistan, Saudi Arabia and the United Arab Emirates)	2005–2020	94	26,029	1.70–99.90	33.03	27.40–39.19	N/A	< 0.0001	98.80	3.53–86.94
Egypt										
All professions	2013–2018	10	2507	20.00–92.90	55.69	41.74–68.79	N/A	< 0.0001	97.50	12.98–91.37
Doctors	2016	2	961	52.00–65.40	58.92	49.34–67.88	0.0716	< 0.0001	88.70	N/A
Residents	2013–2018	4	641	20.00–59.52	40.06	25.51–56.61		< 0.0001	92.40	2.80–93.94
Nurses	2013–2016	4	645	45.00–92.90	69.16	46.25–85.39		< 0.0001	95.90	2.32–99.53
All specialties	No data									
Iran										
All professions	2005–2020	44	11,436	2.90–99.90	29.61	22.16–38.33	N/A	< 0.0001	98.60	2.87–85.67
Health care workers	2010–2020	4	670	43.4. –99.90	87.40	25.69–99.29	0.1089	0.0027	99.30	0.00–100.00
Doctors	2020	1	127	26.80	26.77	19.80–35.13		N/A	N/A	N/A
Residents	2014–2015	2	2449	31.20–81.81	58.60	22.26–87.50		< 0.0001	98.60	N/A
Nurses	2005–2020	36	7961	2.90–89.25	23.84	18.74–29.83		< 0.0001	96.70	4.58–67.14
Allied health staff	2020	1	229	24.00	24.02	18.92–29.97		N/A	N/A	N/A
All specialties	2005–2020	44	11,436	2.90–99.90	29.61	22.16–38.33	N/A	< 0.0001	98.60	2.87–85.67
Emergency medicine	2014–2015	1	198	81.81	81.82	75.82–86.59	< 0.0001	N/A	N/A	N/A
Lebanon										
All Professions	2013	4	171	8.00–46.00	22.98	9.93–44.69	N/A	0.0062	81.30	0.41–95.63
Residents	2013	3	80	8.00–46.00	17.75	5.46–44.65	0.0081	0.0026	73.70	0.00–100.00
Nurses	2013	1	91	36.20	36.26	27.07–46.59		N/A	N/A	N/A
All specialties	2013	4	171	8.00–46.00	22.98	9.93–44.69	N/A	0.0062	81.30	0.41–95.63
Internal medicine	2013	1	35	46.00	45.71	30.22–62.09	0.0062	N/A	N/A	N/A
Surgery	2013	1	25	8.00	8.00	2.01–26.94		N/A	N/A	N/A
Obestetrics & Gynecology	2013	1	20	12.00	10.00	2.51–32.38		N/A	N/A	N/A
Oman										
All professions	2017	4	531	1.70–11.40	3.75	1.54–8.84	N/A	0.0023	69.9	0.09–61.61
Residents	2017	4	531	1.70–11.40	3.75	1.54–8.84	N/A	0.0023	69.9	0.09–61.61
All specialties	2017	4	531	1.70–11.40	3.75	1.54–8.84	N/A	0.0023	69.9	0.09–61.61
Internal Medicine	2017	1	235	3.00–11.40	2.98	1.43–6.11	0.0023	N/A	N/A	N/A
Surgery	2017	1	119	1.70	1.68	0.42–6.47		N/A	N/A	N/A
Radiology	2017	1	45	2.2	2.22	0.31–14.16		N/A	N/A	N/A
Pakistan										
All professions	2008–2020	11	2196	6.10–85.30	31.69	18.75–48.26	N/A	< 0.0001	97.80	2.89–87.86
Health care workers	2020	3	882	27.30–34.60	31.42	28.00–35.05	0.0081	0.1375	24.00	10.20–64.88
Doctors	2008–2018	6	1209	6.10–85.30	27.53	9.87–56.85		< 0.0001	97.40	0.37–97.46
Residents	2020	1	35	39.90	40.00	25.32–56.73		N/A	N/A	N/A
Nurses	Not reported	1	70	51.40	51.43	39.85–62.85		N/A	N/A	N/A
All specialties	No data									
Saudi Arabia										
All professions	2012–2020	19	4211	7.10–70.60	35.70	26.96–45.51	N/A	< 0.0001	96.80	7.62–78.90
Health Care Workers	2013–2020	2	527	35.47–52.20	42.39	31.33–54.27	0.3543	0.0032	76.50	N/A
Doctors	2015–2016	6	812	22.00–47.00	35.69	28.87–43.15		0.0002	76.20	16.50–60.93
Residents	2012–2018	6	610	19.90–70.60	46.34	27.55–66.22		< 0.0001	94.40	4.30–94.32
Nurses	2014–2017	3	1918	7.10–53.80	17.78	5.15–46.27		< 0.0001	98.90	0.00–100.00
EMTs	2017–2019	2	344	26.70–41.00	32.89	23.63–43.71		0.0073	72.00	N/A
All specialties	2012–2020	19	4211	7.10–70.60	35.70	26.96–45.51	N/A	< 0.0001	96.80	7.62–78.90
Internal medicine	Not reported	1	149	40.20	40.27	32.70–48.33	< 0.0001	N/A	N/A	N/A
Surgery	2017	1	34	70.60	70.59	53.44–83.39		N/A	N/A	N/A
Emergency medicine	2015–2016	2	205	31.40–41.00	34.63	28.44–41.40		0.1666	0.00	N/A
Family medicine	2015–2016	1	69	22.00	21.74	13.55–32.98		N/A	N/A	N/A
UAE										
All professions	2008–2016	5	727	7.80–65.50	44.2	22.60–68.25	N/A	< 0.0001	97.00	1.62–97.45
Residents	2012–2016	4	634	50.00–65.50	58.47	51.15–65.43	< 0.0001	0.0066	63.9	29.95–82.25
Allied Health Staff	2008	1	93	7.80	7.53	3.63–14.96		N/A	N/A	N/A
All specialties	No data									

*EMT* Emergency Medical Technician

^a^Only calculated when 3 or more data points available

### Time trend of depression prevalence

The earliest reported prevalence data in our systematic review was among nurses in Iran, dating back to 2005, while the newest data is reported among all HCWs in Saudi Arabia in May 2020. Prior to 2011, the pooled prevalence of depression among all HCWs across seven EMR countries was 25.80% (95% CI = 17.84–35.76%), compared to 33.87% (95% CI = 25.05–43.99%) in 2011–2019. Prevalence appreciably increased in 2020 [46.12% (95% CI = 21.30–73.02%)] after the appearance of the COVID-19 pandemic. Over time (2005–2020), depression prevalence has notably increased, although this difference is not statistically significant.

### Overview of depression prevalence by HCW profession and specialty

In the EMR, the highest and lowest depression prevalence measures among the different professions and specialties were observed in Iran. The depression prevalence ranged from 0.90% among nurses to 99.90% among medical staff (profession not specified). The pooled prevalence of depression ranged from 26.39% (95% CI = 7.97–59.74%) among allied health staff to 55.22% in the category of all HCWs (95% CI = 33.14–75.41%) (Table 1). The highest pooled prevalence was found among nurses in Egypt [69.61% (95% CI = 46.25–85.39%)], and the lowest among medical resident physicians in Oman [3.75% (95% CI = 1.54–8.84%)] (Table 3). The pooled prevalence among all resident physicians in the EMR [32.78% (95% CI = 22.08–45.63%)] mirrored that of other HCW professions. No statistical difference in depression prevalence was identified between different HCW professions.

The pooled prevalence of depression varied significantly between specialties (*p* = 0.0001). Emergency medicine HCWs who primarily work in emergency departments were at the highest risk of suffering from depression [pooled prevalence = 53.34% (95% CI = 26.63–77.99%)] compared to other specialties. Radiologists were found to have the lowest pooled prevalence of depression at 2.22% (95% CI = 0.31–14.16%).

### Overview of depression prevalence by sex

Among males, the depression prevalence varied from 9.50% among all medical resident physicians in Oman to 76.10% among dental surgeons in Pakistan. The depression prevalence among females ranged from a low of 2.90% among nurses working in the emergency department in Iran to a high of 70.10% among all HCWs (profession and specialty not specified) in Iraq.

Only eight prevalence measures [53–60] on the levels of depression among males and females were eligible to be included in the meta-analysis. Despite the prevalence in males [53.43% (95% CI = 27.43–77.69%)] being approximately double that in females [23.35% (95% CI = 8.24–50.82%)], no statistically significant difference between male and female HCWs in the EMR was detected (*p* = 0.1154).

### Heterogeneity in studies describing depression prevalence

Strong heterogeneity in depression prevalence was evident in the different subgroup meta-analyses (*p* value < 0.0001). The I^2^ for the pooled estimates indicated that most of the variability in depression prevalence was due to heterogeneity between studies rather than chance (I^2^ > 50%). Consequently, the prediction intervals were broad, confirming substantial variability between studies in measuring the depression prevalence.

### Risk and protective factors for depression among HCWs

**Fig. 3:**
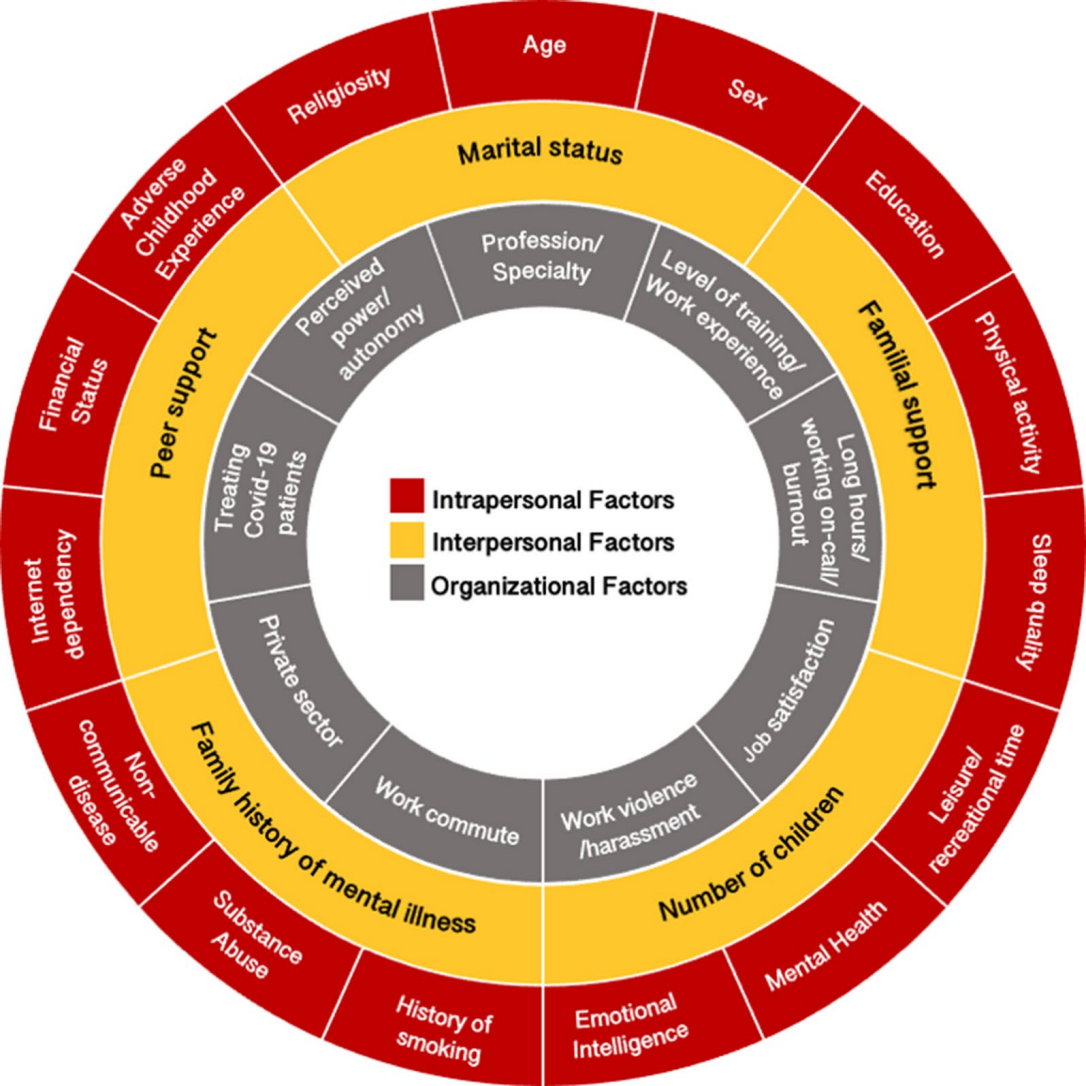
Risk and protective factors of depression among EMR HCWs

Ninety-two primary studies from 12 EMR countries reported data on risk and protective factors for depression among HCWs. We summarized the identified risk and protective factors as intrapersonal, interpersonal, and organizational categories in Fig. 3. The most commonly identified intrapersonal factors include sex, age, and sleep quality. Specifically, primary studies reported female HCWs being at a higher risk of suffering from depressive symptoms as compared to males. A dearth of good quality sleep (as reported in the primary studies) was associated with depressive symptoms, and while age was frequently mentioned as a risk factor, the data (young vs old) was contradictory among the studies. The commonly associated interpersonal factors were marital status, but as with age, the results (single vs married) were inconsistent. Among organizational factors, the profession and specialty, long work hours as reported in the primary studies (including being on-call), level of training, and years of work experience were the key factors predisposing to depression. Detailed information regarding risk and protective factors are presented in Additional file 1: Additional material 5.

We also provide a summary of recommendations to manage depression among HCWs by the authors of the primary studies (Table 4). We classified the reported recommendations into three categories: (i) prevention of depression onset, and early recognition of depression; (ii) provision of counseling and mental health services; and (iii) specific research recommendations to enhance the diagnosis and management of depression in HCWs. Detailed recommendations reported by the primary studies are provided in Additional file 1: Additional material 5.

**Table 4:** Summary of recommendations

Prevention	
Regular screening of HCWs [1–11] (Institutional level)	
Assign mentors for HCWs to exchange knowledge and experience [12–15] (Institutional level)	
Programs to encourage awareness, build resilience and healthy lifestyle behaviors, study skills, and reduce stigma [1, 2, 4–6, 9, 12, 13, 16–38] (Governmental, Community & Institutional levels)	
Reforming institutional policies and facilities to enhance work-life balance and ensure safe work environment [3, 8, 12, 13, 19–22, 24, 26–33, 35, 36, 38–46] (Institutional level)	
Provide support to ensure financial stability of communities [4] (Governmental & Community levels)	
Treatment	
Appropriate psychiatric/religious counselling and mental health services for HCWs at risk of or suffering depression [2, 9, 10, 13, 20, 22, 32, 35, 47–52] (Institutional level)	
Governmental level support for healthcare institutional programs [33, 43, 44] (Governmental level)	
Research	
Development and use of better diagnostic instruments [8, 50] (Institutional level)	
Further detailed research to study prevalence and consequences of depression [6, 7, 12, 14, 16, 23, 29, 38, 42, 48, 51, 53–55] (Governmental & Institutional level)	

## Discussion

Our systematic review includes 108 primary studies including data from 12 EMR countries. A majority of these studies appropriately described the study participants and setting with adequate sample size and used a validated instrument to identify depressive symptoms or depression; a few studies, however, used non-probability based sampling. The meta-analysis revealed a high prevalence of depression among EMR HCWs—more than one-third of HCWs suffered depressive symptoms or depression. Among all HCWs in the EMR, Egypt demonstrated the highest depression prevalence with more than 50% reporting depressive symptoms, likely due to the heavy workload, severe staffing shortages and low compensation for HCWs in the country [61]. While there was no statistically significant difference in the prevalence of depression between different HCW professions, emergency HCWs had a higher depression prevalence that was statistically significant when compared to their counterparts in other specialties.

Our meta-analysis demonstrates a pooled depression prevalence of 33.03% among HCWs in the EMR countries between 2005 and 2020. Depression was higher in countries, such as Egypt, the UAE, Saudi Arabia, and Pakistan as compared to other countries in the region. The EMR appears to have a unique confluence of factors that predisposes HCWs to depression. HCWs in high income countries of the EMR, namely, Bahrain, Kuwait, Oman, Qatar, Saudi Arabia, and the UAE, face stigma, since mental health illness is perceived negatively. This is similar to other countries worldwide [6, 62–64]. On the other hand, Oman had very low rates of depression (3.75%), possibly due to limited data. The large range in rates of HCW depression is similar to the variations observed in OECD countries, such as in Australia (21.00–60.00%) [65, 66] and the UK (11.30–36.10%) [67, 68]. Lower middle-income countries, such as Bangladesh (27.30%) [69], Philippines (16.90%) [70] and India (27.71%) [71]) appear to have comparable depression rates to HCWs from similar settings in the EMR. Several low- or lower middle-income EMR countries, such as Iraq, Libya, Pakistan, Syria, Tunisia, and Yemen have ongoing unrest, economic fragility, and political instability. This may result in additional stress and workload for HCWs in these countries [17]. Consequently, substantial variation in depression prevalence exists between these countries.

The increase in depression prevalence from before 2011 and between 2011 and 2019 in our study might be the result of increasing pressure and workload on HCWs as a result of: healthcare systems becoming increasingly commercialized in the private sector; political upheaval in the low- and lower middle-income countries, a lack of resources and highly trained healthcare workforce, potentially an artifact of increasing awareness and a reduction in stigma against disclosing depressive symptoms [18, 64, 72, 73]. The current COVID-19 pandemic continues to impose an increasing strain on healthcare systems and HCWs, which has directly led to elevated psychiatric morbidity among HCWs [74]. This is borne out in our data, with a substantial (but statistically insignificant) rise in the depression prevalence during 2020 [13–15]. However, it is noteworthy that several studies reporting this data in 2020 were preprints and not yet published in peer-reviewed journals.

The prevalence of depressive symptoms or depression among EMR resident physicians is 32.78%. This is similar to the depression prevalence in resident physicians worldwide between 1972 and 2012 as determined by Mata et al*.* (28.80%, prevalence range: 20.90–43.20%) [6]. Depression does not manifest only when professional life begins and may appear even earlier in student life. In their meta-analysis, Rotenstein et al*.* report that 27.20% of medical students globally suffer from depression [75]. Furthermore, one cross-sectional study reports that 72.50% of resident physicians who screen positive for depression did not actively seek treatment [76]. This can compromise the quality of patient care and safety. Depressed resident physicians commit significantly more medical errors than do their peers [77]. Overall, the data suggest that health professions students are at risk for depression and remain so throughout their careers. Thus, it is critical to educate students about recognizing depression and to cultivate self-care (daily practice of 20–30 min of stretching, meditation, exercise etc.) as part and parcel of the health professions curriculum and training, so they are better able to cope with the stresses of their future careers.

Our meta-analysis demonstrates that the HCWs from emergency departments had statistically significant higher measures of depression as compared to HCWs from other departments (*p* = 0.0001). This is consistent with previously published literature, as emergency HCWs work in high pressure environments, sometimes with limited resources, and self-report higher levels of depressive symptoms [78, 79]. It is incumbent on healthcare systems to pay attention to emergency HCWs and provide them with an enabling environment, inclusive of tools to help build their mental resilience along with access to peer support groups.

A differential sex burden was frequently described in the identified primary studies, with females at an elevated risk of depression or depressive symptoms. This is similar to global reports [80, 81]. However, our meta-analysis did not yield a higher depression prevalence among females. This discrepancy is likely due to the small number of included primary studies that reported depression prevalence data by sex. The results of other commonly cited risk factors for depression/depressive symptoms, such as marital status, age, level of education, and amount of work experience, were unclear and often contradictory. There is a need for additional research to identify risk factors unique to the EMR.

To implement comprehensive interventions for the prevention of depression, several factors must be considered within the personal, social, and institutional domains. Prominent among these considerations is the need to build resilience and positive coping mechanisms among HCWs to ameliorate depression risk. Such strategies may include meditation, self-care including paying attention to healthy nutrition, exercise, and sleep, and cognitive behavioral therapy (CBT) [82]. Ensuring regular screening and the availability of appropriate and anonymized mental health services is recommended. For HCWs, where such services are already available, the often-mentioned barriers to the uptake of these services include lack of time, inconvenient access, absence of confidentiality, and a preference to self-manage [83]. These concerns must be addressed to result in substantive change. A 2015 Cochrane review reported that person-directed interventions (such as relaxation techniques or CBT) were more effective in alleviating stress among HCWs compared to organizational interventions (such as targeting stress reduction in the workplace) [84]. Nevertheless, reforming institutional policies (such as organizing peer support sessions), minimizing system-based causes (ensuring HCW autonomy and safety), and changing work culture (including rest breaks) should be deemed imperative. Ideally, healthcare systems should adopt a more upstream approach when it comes to implementing such policies. In a longitudinal study conducted in Japan, the Sense of Coherence Scale was a useful indicator for the development of future new-onset depressive symptoms among resident physicians who were not currently depressed [85]. Customizing and using this scale in concurrence with policies to monitor and support HCWs may help proactively alleviate the depression burden.

This systematic review and meta-analysis on depression among HCWs in the EMR has several strengths: rigorous and meticulous methodology, multiple database search strategy inclusive of both scholarly and gray literature, and no language or time restrictions applied to the search strategy. To our knowledge, this is the first study to systematically include gray literature on depression prevalence from the EMR. Gray literature is an important source of disease epidemiology within the EMR due to multiple regional languages and publication in non‐indexed journals affiliated to local universities [29]. We summarize data on several countries in the region that will enable them to tailor specific guidelines to monitor and treat depression among HCWs. However, our systematic review and meta-analysis also has some limitations. Most primary studies were cross-sectional in design—this is especially pertinent for depression, as the severity of depression may fluctuate over time as circumstances change. Hence, we may not have a comprehensive picture of the true prevalence of depression/depressive symptoms within the EMR. The primary studies included in our review used numerous instruments with varying cutoffs, resulting in considerable heterogeneity. In addition, the quality of the included studies varied—few studies used appropriate sampling methods, but most studies described the study participants well. We were confined to data from only 12 out of the 21 EMR countries and Palestine. In some instances, the sample population was described as ‘medical staff’. This may have included administrative staff, who are not our population of interest. Finally, we included preprints in our systematic review to evaluate data within the context of the COVID-19 pandemic, but these studies were not peer reviewed and hence their results cannot be substantiated.

Our primary search strategy identified 31 studies, compared to 77 studies by the supplementary search strategy. This is unsurprising as many studies reporting on the disease burden in the EMR are likely published as part of the gray literature and in a language other than English [28, 29]. This emphasizes the need for including and assessing gray literature while conducting research in the region. Of the included studies from Iran, 44.44% were in Persian, so we may have missed pertinent data. However, we extracted available relevant data from the English language abstract. In addition, we included data from any (excluded) systematic review that described relevant data for these primary studies. For the other EMR countries, our screening and data extraction were comprehensive, as the authors are proficient in English, Arabic, Urdu, and French.

The findings of this systematic review and meta-analysis are highly relevant to inform governments, healthcare systems, training institutions, and other pertinent stakeholders in the EMR. The scale and burden of depression amongst HCWs have been overlooked worldwide, and this is especially true of the EMR. Determining the magnitude of the problem, as well as the factors associated with depression, is the first step in devising effective interventions to address depression in this population.

## Conclusion

Optimal mental health of HCWs is imperative for societal good and ensuring their individual well-being. Depression amongst HCWs, including EMR HCWs, is concerning. Our findings for depression amongst HCWs in the EMR are similar to the global prevalence estimates for this population. Prospective, longitudinal studies using standardized instruments to determine the depression prevalence among EMR HCWs with greater precision are required. De-stigmatization and the promotion of an inclusive, non-judgmental work culture can help reduce the toll of depression. The establishment of support systems and mentoring programs is critical to building resilience among HCWs. Furthermore, legislative efforts toward establishing policies that allow for early detection and management of mental health problems among trainees and HCWs are required. Addressing mental health problems among HCWs should be prioritized, so they can enjoy a good quality of life and at the same time feel healthy to provide quality care to their patients.

## Supplementary Information

**Additional file 1.** Additional Information.

## Data Availability

The data set(s) supporting the conclusions of this article is(are) included within the article (and its additional file(s)).

## Abbreviations

CI: Confidence Interval
CIDI: WHO Composite International Diagnostic Interview
DSM-IV: Diagnostic and Statistical Manual of Mental Disorders-IV
EMR: Eastern Mediterranean Region
EMT: Emergency Medical Technicians
HCW: Health Care Worker
MMPI-2: Minnesota Multiphasic Personality Inventory-2
PRISMA: Preferred Reporting Items for Systematic Reviews and Meta-Analyses
PSE-10/SCAN: Present State Examination/Schedules for Clinical Assessment in Neuropsychiatry
UAE: United Arab Emirates
WHO: World Health Organization
DASS-42: Depression Anxiety and Stress Scale
DASS-21: Depression Anxiety and Stress Scale-21
