# Supplementary material for: Depression among healthcare workers in the Eastern Mediterranean Region: a systematic review and meta-analysis
Source: Hum Resour Health. 2021 Jul 10;19:81. doi: 10.1186/s12960-021-00628-6 (PMC8271293; doi:10.1186/s12960-021-00628-6)
Supplement: Supplementary file 1 — Additional file 1. Additional Information. [file 12960_2021_628_MOESM1_ESM.docx]

**Depression among Healthcare Workers in the Eastern Mediterranean Region: A Systematic Review and Meta-Analysis**

**Social Psychiatry and Psychiatric Epidemiology**

Authors: Amit Abraham (ORCID: 0000-0002-9696-8598)^1^, Karima Chaabna (ORCID: 0000-0002-1443-207X)^1^, Sathyanarayanan Doraiswamy (ORCID: 0000-0002-4198-8065)^1^, Sapna Bhagat (ORCID: 0000-0002-3764-8244)^1^, Javaid Sheikh (ORCID: 0000-0002-5762-4186)^2^, Ravinder Mamtani (ORCID: 0000-0001-8507-281X)^1^*, Sohaila Cheema (ORCID: 0000-0002-5874-4059)^1^*

*Equal senior author contribution

Affiliations:

1 - Institute for Population Health, Weill Cornell Medicine-Qatar, Doha, Qatar

2 – Office of the Dean, Weill Cornell Medicine-Qatar, Doha, Qatar

Corresponding Author:

Dr. Amit Abraham

Institute for Population Health

Weill Cornell Medicine - Qatar

Education City, Qatar Foundation

Doha, Qatar

P.O. Box 24144

Telephone: +974 44928528

Fax: +974 44928333

Email: [ama2006@qatar-med.cornell.edu](mailto:ama2006@qatar-med.cornell.edu)

**Captions**

Additional Material 1. The 2009 PRISMA checklist for reporting a systematic review.

Additional Material 2: The PRISMA for Abstracts checklist

Additional Material 3: Search strategy

Additional Material 4: Characteristics of included studies on depression in healthcare workers in the EMR

Additional Material 5: Risk / Protective Factors and Recommendations

Additional Material 6: Excluded Studies

Additional Material 7: Quality assessment of primary studies

Additional Material 8: Depression prevalence by instrument

**Additional Material 1. The 2009 PRISMA checklist for reporting a systematic review**

| **Section/topic** | **#** | **Checklist item** | **Reported on page #** |
| --- | --- | --- | --- |
| **TITLE** | | |  |
| Title | 1 | Identify the report as a systematic review, meta-analysis, or both. | 1 |
| **ABSTRACT** | | |  |
| Structured summary | 2 | Provide a structured summary including, as applicable: background; objectives; data sources; study eligibility criteria, participants, and interventions; study appraisal and synthesis methods; results; limitations; conclusions and implications of key findings; systematic review registration number. | 2 |
| **INTRODUCTION** | | |  |
| Rationale | 3 | Describe the rationale for the review in the context of what is already known. | 3 |
| Objectives | 4 | Provide an explicit statement of questions being addressed with reference to participants, interventions, comparisons, outcomes, and study design (PICOS). | 3 |
| **METHODS** | | |  |
| Protocol and registration | 5 | Indicate if a review protocol exists, if and where it can be accessed (e.g., Web address), and, if available, provide registration information including registration number. | 3 |
| Eligibility criteria | 6 | Specify study characteristics (e.g., PICOS, length of follow-up) and report characteristics (e.g., years considered, language, publication status) used as criteria for eligibility, giving rationale. | 4 |
| Information sources | 7 | Describe all information sources (e.g., databases with dates of coverage, contact with study authors to identify additional studies) in the search and date last searched. | 4 |
| Search | 8 | Present full electronic search strategy for at least one database, including any limits used, such that it could be repeated. | 4, Additional Material 3 |
| Study selection | 9 | State the process for selecting studies (i.e., screening, eligibility, included in systematic review, and, if applicable, included in the meta-analysis). | 4-5 |
| Data collection process | 10 | Describe method of data extraction from reports (e.g., piloted forms, independently, in duplicate) and any processes for obtaining and confirming data from investigators. | 5 |
| Data items | 11 | List and define all variables for which data were sought (e.g., PICOS, funding sources) and any assumptions and simplifications made. | 5 |
| Risk of bias in individual studies | 12 | Describe methods used for assessing risk of bias of individual studies (including specification of whether this was done at the study or outcome level), and how this information is to be used in any data synthesis. | 5 |
| Summary measures | 13 | State the principal summary measures (e.g., risk ratio, difference in means). | 5-6 |
| Synthesis of results | 14 | Describe the methods of handling data and combining results of studies, if done, including measures of consistency (e.g., I^2^) for each meta-analysis. | 5-6 |
| Section/topic | # | Checklist item |  |
| Risk of bias across studies | 15 | Specify any assessment of risk of bias that may affect the cumulative evidence (e.g., publication bias, selective reporting within studies). | 5-6 |
| Additional analyses | 16 | Describe methods of additional analyses (e.g., sensitivity or subgroup analyses, meta-regression), if done, indicating which were pre-specified. | 6 |
| **RESULTS** | | |  |
| Study selection | 17 | Give numbers of studies screened, assessed for eligibility, and included in the review, with reasons for exclusions at each stage, ideally with a flow diagram. | 7-8, Figure 1 |
| Study characteristics | 18 | For each study, present characteristics for which data were extracted (e.g., study size, PICOS, follow-up period) and provide the citations. | 7-8 |
| Risk of bias within studies | 19 | Present data on risk of bias of each study and, if available, any outcome level assessment (see item 12). | 8, Additional Material 7 |
| Results of individual studies | 20 | For all outcomes considered (benefits or harms), present, for each study: (a) simple summary data for each intervention group (b) effect estimates and confidence intervals, ideally with a forest plot. | Additional Material 5, 6 |
| Synthesis of results | 21 | Present results of each meta-analysis done, including confidence intervals and measures of consistency. | 8-10, Tables 1-3 |
| Risk of bias across studies | 22 | Present results of any assessment of risk of bias across studies (see Item 15). | 8-10 |
| Additional analysis | 23 | Give results of additional analyses, if done (e.g., sensitivity or subgroup analyses, meta-regression [see Item 16]). | 8-10, Table 2 |
| **DISCUSSION** | | |  |
| Summary of evidence | 24 | Summarize the main findings including the strength of evidence for each main outcome; consider their relevance to key groups (e.g., healthcare providers, users, and policy makers). | 11-14 |
| Limitations | 25 | Discuss limitations at study and outcome level (e.g., risk of bias), and at review-level (e.g., incomplete retrieval of identified research, reporting bias). | 13 |
| Conclusions | 26 | Provide a general interpretation of the results in the context of other evidence, and implications for future research. | 13 |
| **FUNDING** | | |  |
| Funding | 27 | Describe sources of funding for the systematic review and other support (e.g., supply of data); role of funders for the systematic review. | 14 |

Source: Moher D, et al., 2009 [1]

Available at: <http://www.prisma-statement.org/documents/PRISMA%202009%20checklist.pdf>

**Additional Material 2: The PRISMA for Abstracts checklist**

| **TITLE** | **CHECKLIST ITEM** | **REPORTED ON PAGE #** |
| --- | --- | --- |
| 1. Title: | Identify the report as a systematic review, meta-analysis, or both. | 1 |
| **BACKGROUND** |  |  |
| 2. Objectives: | The research question including components such as participants, interventions, comparators, and outcomes. | 2 |
| **METHODS** |  |  |
| 3. Eligibility criteria: | Study and report characteristics used as criteria for inclusion. | 2 |
| 4. Information sources: | Key databases searched and search dates. | 2 |
| 5. Risk of bias: | Methods of assessing risk of bias. | 2 |
| **RESULTS** |  |  |
| 6. Included studies: | Number and type of included studies and participants and relevant characteristics of studies. | 2 |
| 7. Synthesis of results: | Results for main outcomes (benefits and harms), preferably indicating the number of studies and participants for each. If meta-analysis was done, include summary measures and confidence intervals. | 2 |
| 8. Description of the effect: | Direction of the effect (i.e. which group is favoured) and size of the effect in terms meaningful to clinicians and patients. | 2 |
| **DISCUSSION** |  |  |
| 9. Strengths and Limitations of evidence: | Brief summary of strengths and limitations of evidence (e.g. inconsistency, imprecision, indirectness, or risk of bias, other supporting or conflicting evidence) | 2 |
| 10. Interpretation: | General interpretation of the results and important implications | 2 |
| **OTHER** |  |  |
| 11. Funding: | Primary source of funding for the review. | 2 |
| 12. Registration: | Registration number and registry name. | 2 |

Source: Beller EM, et al., 2013 [2]

Available at: <http://www.prisma-statement.org/Extensions/Abstracts.aspx>

**Additional Material 3: Search strategy**

| **PubMed Query** | | | **Notes** | **Items found** | **Date Last Updated** | |
| --- | --- | --- | --- | --- | --- | --- |
| (((((((("Depression"[Mesh] OR "Depressive Disorder"[Mesh] OR "Bipolar Disorder"[Mesh] OR "Adjustment Disorders"[Mesh] OR "Affective Disorders, Psychotic"[Mesh] OR Depress*[Text Word] OR Melancholi*[Text Word] OR "Involutional Psychosis"[Text Word] OR "Involutional Psychoses"[Text Word] OR "Involutional Paraphrenia"[Text Word] OR "Involutional Paraphrenias"[Text Word] OR Dysthymi*[Text Word] OR "Reactive Disorders"[Text Word] OR "Reactive Disorder"[Text Word] OR "Adjustment Disorder"[Text Word] OR "Adjustment Disorders"[Text Word] OR "Anniversary Reaction"[Text Word] OR "Anniversary Reactions"[Text Word] OR "Transient Situational Disturbance"[Text Word] OR Bipolar[Text Word] OR Manic[Text Word] OR Mania[Text Word] OR Manias[Text Word] OR "Affective Psychoses"[Text Word] OR "Affective Psychosis"[Text Word] OR "Psychotic Affective Disorders"[Text Word] OR "Psychotic Mood Disorders"[Text Word] OR "Psychotic Mood Disorder"[Text Word] OR "Psychotic Affective Disorder"[Text Word]))))))) AND (("medical staff"[Text Word] or "healthcare staff"[Text Word] or "health care staff"[Text Word] OR "Healthcare provider"[Text Word] OR "Healthcare providers"[Text Word] OR "Healthcare practitioner"[Text Word] OR "Healthcare practitioners"[Text Word] OR "Healthcare worker"[Text Word] OR "Healthcare workers"[Text Word] OR "Healthcare personnel"[Text Word] OR "Healthcare profession”[Text Word] OR "Healthcare professions”[Text Word] OR "Health care provider"[Text Word] OR "Health care providers"[Text Word] OR "Health care practitioner"[Text Word] OR "Health care practitioners"[Text Word] OR "Health care worker"[Text Word] OR "Health care workers"[Text Word] OR "Health care personnel"[Text Word] OR "Health care profession”[Text Word] OR "Health care professions”[Text Word] OR “Medical professional”[Text Word] OR “Medical professionals”[Text Word] OR “Medical personnel”[Text Word] OR “Medical provider”[Text Word] OR “Medical providers”[Text Word] OR “Medical practitioner”[Text Word] OR “Medical practitioners”[Text Word] OR “Medical profession”[Text Word] OR “Medical professions”[Text Word] OR "Emergency Medicine Technicians"[Text Word] OR "Emergency Medicine Technician"[Text Word] OR Paramedic*[Text Word] OR dentist*[Text Word] OR dental*[Text Word] OR Endodont*[Text Word] OR pedodont*[Text Word] OR paedodont*[Text Word] OR orthodont*[Text Word] OR Periodont*[Text Word] OR Prosthodont*[Text Word] OR Nurs*[Text Word] OR Pharmac*[Text Word] OR Doctor*[Text Word] OR Physician*[Text Word] OR Clinician*[Text Word] OR “Occupational therapist”[Text Word] OR Anesthesiologist* [Text Word] OR Anesthetist*[Text Word] OR Anaesthetist*[Text Word] OR Anaesthesiologist* [Text Word] OR Cardiologist*[Text Word] OR Dermatologist*[Text Word] OR Endocrinologist*[Text Word] OR Gastroenterologist*[Text Word] OR Hepatologist*[Text Word] OR “General Practitioner”[Text Word] OR “General Practitioners”[Text Word] OR Geriatrician*[Text Word] OR Gerontologist*[Text Word] OR Gynecologist*[Text Word] OR Gynaecologist*[Text Word] OR Hospitalist*[Text Word] OR internist*[Text Word] OR Nephrologist*[Text Word] OR Neurologist*[Text Word] OR Obstetrician*[Text Word] OR Orthopedic*[Text Word]OR Orthopaedic*[Text Word] OR Oncologist*[Text Word] OR Ophthalmologist*[Text Word] OR Osteopath*[Text Word] OR Otologist*[Text Word] OR Otolaryngologist*[Text Word] OR Pathologist*[Text Word] OR Pediatrician*[Text Word] OR Paediatrician*[Text Word] OR Physiatrist*[Text Word ]OR Psychatrist*[Text Word] OR Pulmonologist*[Text Word] OR Radiologist*[Text Word] OR Rheumatologist*[Text Word] OR Surgeon*[Text Word] OR Urologist*[Text Word] OR “Occupational therapists”[Text Word] OR “Physical therapist”[Text Word] OR “Physical therapists”[Text Word] OR Physiotherapist*[Text Word] OR health personnel[MeSH Terms] AND ((("Qatar"[Mesh] OR "Bahrain"[Mesh] OR "Oman"[Mesh] OR "Saudi Arabia"[Mesh] OR "Kuwait"[Mesh] OR "United Arab Emirates"[Mesh] OR "Yemen"[Mesh] OR "Egypt"[Mesh] OR "Jordan"[Mesh] OR "Lebanon"[Mesh] OR "Syria"[Mesh] OR "Iraq"[Mesh] OR "Iran"[Mesh] OR "Somalia"[Mesh] OR "Afghanistan"[Mesh] OR "Libya"[Mesh] OR "Morocco"[Mesh] OR "Tunisia"[Mesh] OR "Djibouti"[Mesh] OR "Sudan"[Mesh] OR "South Sudan"[Mesh] OR "Pakistan"[Mesh] OR "Africa, Northern"[Mesh] OR "Africa, Eastern"[Mesh] OR "middle east"[Mesh] OR "Arabs"[Mesh] OR "UAE"[Text Word] OR "U.A.E"[Text Word] OR Emirat*[Text Word] OR "United Arab Emirates"[Text Word] OR Qatar*[Text Word] OR Oman*[Text Word] OR (Saudi[Text Word] AND Arabia*[Text Word]) OR Saudi*[Text Word] OR Kuwait*[Text Word] OR Bahrain*[Text Word] OR Yemen*[Text Word] OR Egypt*[Text Word] OR Jordan*[Text Word] OR Leban*[Text Word] OR Syria*[Text Word] OR Iraq*[Text Word] OR "West Bank"[Text Word] OR Gaza*[Text Word] OR Palestin*[Text Word] OR Iran*[Text Word] OR Somali*[Text Word] OR Afghan*[Text Word] OR Libya*[Text Word] OR Morocc*[Text Word] OR Tunis*[Text Word] OR Djibouti*[Text Word] OR Sudan*[Text Word] OR South Sudan*[Text Word] OR Pakistan*[Text Word] OR (North[Text Word] AND Africa*[Text Word]) OR North-Africa*[Text Word] OR ("Africa"[Text Word] AND "Northern"[Text Word]) OR "Northern Africa"[Text Word] OR "East Africa"[Text Word] OR ("Africa"[Text Word] AND "Eastern"[Text Word]) OR "Maghreb"[Text Word] OR "Maghrib"[Text Word] OR Arab*[Text Word] OR Bedouin*[Text Word] OR "Gulf Cooperation Council"[Text Word] OR "GCC"[Text Word] OR "Middle East"[Text Word]))))) | | | No filters | 1,448 | 16-Oct-19 | |
| **Embase Query** | **Notes** | **Items Found** | | **Date Last Updated** | |  |
| (exp Middle East/ or exp North Africa/ or exp Arab/ or exp Djibouti/ or exp Pakistan/ or exp Sudan/ or exp South Sudan/ or exp Iran/ or exp Afghanistan/ or exp Somalia/ or exp Bahrain/ or exp Egypt/ or exp Jordan/ or exp Kuwait/ or exp Lebanon/ or exp Libya/ or exp Iraq/ or exp Morocco/ or exp Oman/ or exp Pakistan/ or exp Qatar/ or exp Saudi Arabia/ or exp Syria/ or exp Tunisia/ or exp United Arab Emirates/ or exp Yemen/ or exp Palestine/ or Middle East.mp. or North Africa.mp. or EMRO.mp. or Eastern Mediterranean.mp. or Arab.mp. or Arabs.mp. or Arab World.mp. or Iran*.mp. or Afghan*.mp. or Somali*.mp. or Bahrain*.mp. or Djibouti.mp. or Egypt*.mp. or Jordan*.mp. or Kuwait*.mp. or Leban*.mp. or Libya*.mp. or Iraq*.mp. or Morocc*.mp. or Oman*.mp. or Pakistan*.mp. or Qatar*.mp. or Saudi*.mp. or Sudan*.mp. or Syria*.mp. or Tunisia*.mp. or United Arab Emirates.mp. or Emirat*.mp. or West Bank.mp. or Ghaza*.mp. or Gaza*.mp. or Palestin*.mp. or Yemen*.mp. or UAE.mp. or KSA.mp.) AND (exp health personnel/ or medical staff.mp. or healthcare staff.mp. or health care staff.mp. or Healthcare provider?.mp. or Healthcare practitioner?.mp. or Healthcare worker?.mp. or Healthcare personnel.mp. or Healthcare profession?.mp. or Health care provider?.mp. or Health care practitioner?.mp. or Health care worker?.mp. or Health care personnel.mp. or Health care profession?.mp. or Medical professional?.mp. or Medical personnel.mp. or Medical provider?.mp. or Medical practitioner?.mp. or Medical profession?.mp. or Emergency Medicine Technician?.mp. or Emergency Medicine Technician.mp. or Paramedic*.mp. or dentist*.mp. or dental*.mp. or Endodont*.mp. or pedodont*.mp. or paedodont*.mp. OR orthodont*.mp. or Periodont*.mp. or Prosthodont*.mp. or Nurs*.mp. or Pharmac*.mp. or Doctor*.mp. or Physician*.mp. or Clinician*.mp. OR Anesthesiologist*.mp. OR Anesthetist*.mp. OR Anaesthetist*.mp OR Anaesthesiologist*.mp. OR Cardiologist*.mp. OR Dermatologist*.mp. OR Endocrinologist*.mp. OR Gastroenterologist*.mp. OR Hepatologist*.mp. OR General Practitioner?.mp. OR Geriatrician*.mp. OR Gerontologist*.mp. OR Gyn?ecologist*.mp. OR Hospitalist*.mp. OR internist*.mp. OR Nephrologist*.mp OR Neurologist*.mp. OR Obstetrician*.mp. OR Orthopedic*.mp. OR Orthopaedic*.mp. OR Oncologist*.mp. OR Ophthalmologist*.mp. OR Osteopath*.mp. OR Otologist*.mp. OR Otolaryngologist*.mp. OR Pathologist*.mp. OR P?ediatrician*.mp. OR Physiatrist*.mp. OR Psychatrist*.mp. OR Pulmonologist*.mp. OR Radiologist*.mp. OR Rheumatologist*.mp. OR Surgeon*.mp. OR Urologist*.mp. OR Occupational therapist?.mp. or Physical therapist?.mp. or Physiotherapist?.mp.) AND (exp depression/ or Depressi*.mp. or bipolar disorder?.mp. or Adjustment Disorder?.mp. or exp adjustment disorder/ or dysphoria.mp. or dysthymia.mp. or melancholia.mp. or mourning syndrome.mp. or Perry syndrome.mp. or pseudodementia.mp. or seasonal affective disorder?.mp. or affective.mp. or exp affective psychosis/ or exp bipolar disorder/ or Melancholi*.mp. or Involutional Psychosis.mp. or Involutional Psychoses.mp. or Involutional Paraphrenia?.mp. or Dysthymi*.mp. or Reactive Disorder?.mp. or Anniversary Reaction?.mp. or Transient Situational Disturbance.mp. or Manic.mp. or Mania?.mp. or Psychotic Mood Disorder?.mp.) | Exclude Medline journals | 624 | | 16-Oct-19 | |  |

| **PsycInfo Query** | **Notes** | **Items Found** | **Date Last Updated** |
| --- | --- | --- | --- |
| DE "Health Personnel" or DE "Paramedics" OR TX("medical staff" or "healthcare staff" or "health care staff" OR "Healthcare provider" OR "Healthcare providers" OR "Healthcare practitioner" OR "Healthcare practitioners" OR "Healthcare worker" OR "Healthcare workers" OR "Healthcare personnel" OR "Healthcare profession” OR "Healthcare professions” OR "Health care provider" OR "Health care providers" OR "Health care practitioner" OR "Health care practitioners" OR "Health care worker" OR "Health care workers" OR "Health care personnel" OR "Health care profession” OR "Health care professions” OR “Medical professional” OR “Medical professionals” OR “Medical personnel” OR “Medical provider” OR “Medical providers” OR “Medical practitioner” OR “Medical practitioners” OR “Medical profession” OR “Medical professions” OR "Emergency Medicine Technicians" OR "Emergency Medicine Technician" OR Paramedic* OR dentist* OR dental* OR Endodont* OR pedodont* OR paedodont* OR orthodont* OR Periodont* OR Prosthodont* OR Nurs* OR Pharmac* OR Doctor* OR Physician* OR Clinician* OR “Occupational therapist” OR Anesthesiologist* OR Anesthetist* OR Anaesthetist* OR Anaesthesiologist* OR Cardiologist* OR Dermatologist* OR Endocrinologist* OR Gastroenterologist* OR Hepatologist* OR “General Practitioner” OR “General Practitioners” OR Geriatrician* OR Gerontologist* OR Gynecologist* OR Gynaecologist* OR Hospitalist* OR internist* OR Nephrologist* OR Neurologist* OR Obstetrician* OR Orthopedic*OR Orthopaedic* OR Oncologist* OR Ophthalmologist* OR Osteopath* OR Otologist* OR Otolaryngologist* OR Pathologist* OR Pediatrician* OR Paediatrician* OR Physiatrist* OR Psychatrist* OR Pulmonologist* OR Radiologist* OR Rheumatologist* OR Surgeon* OR Urologist* OR “Occupational therapists” OR “Physical therapist” OR “Physical therapists” OR Physiotherapist*) AND (DE "Arabs" OR MA qatar OR bahrain OR oman OR "Saudi arabia" OR saudi OR Kuwait OR Arabia* OR "united arab emirates" OR yemen OR egypt OR jordan OR lebanon OR syria OR Iraq OR libya OR morocco OR tunisia OR djibouti OR sudan OR "South sudan" OR pakistan OR "North africa" OR "northern africa" OR "east africa" OR "eastern africa" OR bedouin OR maghreb OR maghrib OR "gulf cooperation council" OR GCC OR SU qatar OR bahrain OR oman OR "Saudi arabia" OR saudi OR Kuwait OR Arabia* OR "united arab emirates" OR yemen OR egypt OR jordan OR lebanon OR syria OR Iraq OR Iran OR Afghanistan OR Somalia OR libya OR morocco OR tunisia OR djibouti OR sudan OR "South sudan" OR pakistan OR "North africa" OR "northern africa" OR "east africa" OR "eastern africa" OR bedouin OR maghreb OR maghrib OR "gulf cooperation council" OR GCC) OR (TX UAE OR TX U.A.E OR TX Emirat* OR TX United Arab Emirates OR TX Qatar* OR TX Oman* OR TX Saudi Arabia* OR TX Saudi* OR TX Kuwait* OR TX Bahrain* OR TX Yemen* OR TX Egypt* OR TX Jordan* OR TX Leban* OR TX Syria* OR TX Iraq* OR TX West Bank OR TX Gaza* OR TX Palestin* OR TX Iran* OR TX Somali* OR TX Afghan* OR TX Libya* OR TX Morocc* OR TX Tunis* OR TX Djibouti* OR TX Sudan* OR TX South Sudan* OR TX Pakistan* OR TX North Africa* OR TX North-Africa* OR TX "Northern Africa" OR TX "East Africa" OR TX "Eastern Africa" OR TX Maghreb OR TX Maghrib OR TX Arab* OR TX Bedouin* OR TX "Gulf Cooperation Council" OR TX GCC OR TX "Middle East") OR (TX UAE OR TX U.A.E OR TX Emirat* OR TX United Arab Emirates OR TX Qatar* OR TX Oman* OR TX Saudi Arabia* OR TX Saudi* OR TX Kuwait* OR TX Bahrain* OR TX Yemen* OR TX Egypt* OR TX Jordan* OR TX Leban* OR TX Syria* OR TX Iraq* OR TX West Bank OR TX Gaza* OR TX Palestin* OR TX Libya* OR TX Morocc* OR TX Tunis* OR TX Djibouti* OR TX Sudan* OR TX South Sudan* OR TX Pakistan* OR TX North Africa* OR TX North-Africa* OR TX "Northern Africa" OR TX "East Africa" OR TX "Eastern Africa" OR TX Maghreb OR TX Maghrib OR TX Arab* OR TX Bedouin* OR TX "Gulf Cooperation Council" OR TX GCC OR TX "Middle East") AND (DE "Major Depression" OR DE "Anaclitic Depression" OR DE "Dysthymic Disorder" OR DE "Endogenous Depression" OR DE "Late Life Depression" OR DE "Postpartum Depression" OR DE "Reactive Depression" OR DE "Recurrent Depression" OR DE "Treatment Resistant Depression") OR (DE "Depression (Emotion)")) OR (DE "Bipolar Disorder" OR DE "Bipolar I Disorder" OR DE "Bipolar II Disorder" OR DE "Cyclothymic Disorder" OR DE "Mania")) OR (DE "Mania" OR DE "Hypomania")) OR (DE "Affective Disorders" OR DE "Disruptive Mood Dysregulation Disorder" OR DE "Major Depression" OR DE "Seasonal Affective Disorder")) OR (DE "Adjustment Disorders") OR DE "Affective Disorders" OR DE "Disruptive Mood Dysregulation Disorder" OR DE "Major Depression" OR DE "Seasonal Affective Disorder" OR TX (Depressi* OR Dysthymi* OR Bipolar OR Mani* OR "Seasonal affective disorder" OR Melanchol* OR "Adjustment disorder" OR coping OR cope OR cyclothymi* OR "Internalizing symptom" OR "Internalizing symptoms" OR dysphor* OR "Reactive disorder" OR "Mood dysregulation" OR "mood disorder" OR "mood disorders" OR Affective psychos?s) | All 3 concepts Limiters - Publication Type: All Journals; Population Group: Human; Document Type: Journal Article Search modes - Boolean/Phrase | 2,127 | 16-Oct-19 |
| DE "Health Personnel" or DE "Paramedics" OR TX("medical staff" or "healthcare staff" or "health care staff" OR "Healthcare provider" OR "Healthcare providers" OR "Healthcare practitioner" OR "Healthcare practitioners" OR "Healthcare worker" OR "Healthcare workers" OR "Healthcare personnel" OR "Healthcare profession” OR "Healthcare professions” OR "Health care provider" OR "Health care providers" OR "Health care practitioner" OR "Health care practitioners" OR "Health care worker" OR "Health care workers" OR "Health care personnel" OR "Health care profession” OR "Health care professions” OR “Medical professional” OR “Medical professionals” OR “Medical personnel” OR “Medical provider” OR “Medical providers” OR “Medical practitioner” OR “Medical practitioners” OR “Medical profession” OR “Medical professions” OR "Emergency Medicine Technicians" OR "Emergency Medicine Technician" OR Paramedic* OR dentist* OR dental* OR Endodont* OR pedodont* OR paedodont* OR orthodont* OR Periodont* OR Prosthodont* OR Nurs* OR Pharmac* OR Doctor* OR Physician* OR Clinician* OR “Occupational therapist” OR Anesthesiologist* OR Anesthetist* OR Anaesthetist* OR Anaesthesiologist* OR Cardiologist* OR Dermatologist* OR Endocrinologist* OR Gastroenterologist* OR Hepatologist* OR “General Practitioner” OR “General Practitioners” OR Geriatrician* OR Gerontologist* OR Gynecologist* OR Gynaecologist* OR Hospitalist* OR internist* OR Nephrologist* OR Neurologist* OR Obstetrician* OR Orthopedic*OR Orthopaedic* OR Oncologist* OR Ophthalmologist* OR Osteopath* OR Otologist* OR Otolaryngologist* OR Pathologist* OR Pediatrician* OR Paediatrician* OR Physiatrist* OR Psychatrist* OR Pulmonologist* OR Radiologist* OR Rheumatologist* OR Surgeon* OR Urologist* OR “Occupational therapists” OR “Physical therapist” OR “Physical therapists” OR Physiotherapist*) AND (DE "Arabs" OR MA qatar OR bahrain OR oman OR "Saudi arabia" OR saudi OR Kuwait OR Arabia* OR "united arab emirates" OR yemen OR egypt OR jordan OR lebanon OR syria OR Iraq OR libya OR morocco OR tunisia OR djibouti OR sudan OR "South sudan" OR pakistan OR "North africa" OR "northern africa" OR "east africa" OR "eastern africa" OR bedouin OR maghreb OR maghrib OR "gulf cooperation council" OR GCC OR SU qatar OR bahrain OR oman OR "Saudi arabia" OR saudi OR Kuwait OR Arabia* OR "united arab emirates" OR yemen OR egypt OR jordan OR lebanon OR syria OR Iraq OR Iran OR Afghanistan OR Somalia OR libya OR morocco OR tunisia OR djibouti OR sudan OR "South sudan" OR pakistan OR "North africa" OR "northern africa" OR "east africa" OR "eastern africa" OR bedouin OR maghreb OR maghrib OR "gulf cooperation council" OR GCC) OR (TX UAE OR TX U.A.E OR TX Emirat* OR TX United Arab Emirates OR TX Qatar* OR TX Oman* OR TX Saudi Arabia* OR TX Saudi* OR TX Kuwait* OR TX Bahrain* OR TX Yemen* OR TX Egypt* OR TX Jordan* OR TX Leban* OR TX Syria* OR TX Iraq* OR TX West Bank OR TX Gaza* OR TX Palestin* OR TX Iran* OR TX Somali* OR TX Afghan* OR TX Libya* OR TX Morocc* OR TX Tunis* OR TX Djibouti* OR TX Sudan* OR TX South Sudan* OR TX Pakistan* OR TX North Africa* OR TX North-Africa* OR TX "Northern Africa" OR TX "East Africa" OR TX "Eastern Africa" OR TX Maghreb OR TX Maghrib OR TX Arab* OR TX Bedouin* OR TX "Gulf Cooperation Council" OR TX GCC OR TX "Middle East") OR (TX UAE OR TX U.A.E OR TX Emirat* OR TX United Arab Emirates OR TX Qatar* OR TX Oman* OR TX Saudi Arabia* OR TX Saudi* OR TX Kuwait* OR TX Bahrain* OR TX Yemen* OR TX Egypt* OR TX Jordan* OR TX Leban* OR TX Syria* OR TX Iraq* OR TX West Bank OR TX Gaza* OR TX Palestin* OR TX Libya* OR TX Morocc* OR TX Tunis* OR TX Djibouti* OR TX Sudan* OR TX South Sudan* OR TX Pakistan* OR TX North Africa* OR TX North-Africa* OR TX "Northern Africa" OR TX "East Africa" OR TX "Eastern Africa" OR TX Maghreb OR TX Maghrib OR TX Arab* OR TX Bedouin* OR TX "Gulf Cooperation Council" OR TX GCC OR TX "Middle East") AND (DE "Major Depression" OR DE "Anaclitic Depression" OR DE "Dysthymic Disorder" OR DE "Endogenous Depression" OR DE "Late Life Depression" OR DE "Postpartum Depression" OR DE "Reactive Depression" OR DE "Recurrent Depression" OR DE "Treatment Resistant Depression") OR (DE "Depression (Emotion)")) OR (DE "Bipolar Disorder" OR DE "Bipolar I Disorder" OR DE "Bipolar II Disorder" OR DE "Cyclothymic Disorder" OR DE "Mania")) OR (DE "Mania" OR DE "Hypomania")) OR (DE "Affective Disorders" OR DE "Disruptive Mood Dysregulation Disorder" OR DE "Major Depression" OR DE "Seasonal Affective Disorder")) OR (DE "Adjustment Disorders") OR DE "Affective Disorders" OR DE "Disruptive Mood Dysregulation Disorder" OR DE "Major Depression" OR DE "Seasonal Affective Disorder" OR TX (Depressi* OR Dysthymi* OR Bipolar OR Mani* OR "Seasonal affective disorder" OR Melanchol* OR "Adjustment disorder" OR coping OR cope OR cyclothymi* OR "Internalizing symptom" OR "Internalizing symptoms" OR dysphor* OR "Reactive disorder" OR "Mood dysregulation" OR "mood disorder" OR "mood disorders" OR Affective psychos?s) | All 3 concepts Limiters - Publication Type: Dissertation Abstract; Population Group: Human Search modes - Boolean/Phrase | 69 | 16-Oct-19 |
| DE "Health Personnel" or DE "Paramedics" OR TX("medical staff" or "healthcare staff" or "health care staff" OR "Healthcare provider" OR "Healthcare providers" OR "Healthcare practitioner" OR "Healthcare practitioners" OR "Healthcare worker" OR "Healthcare workers" OR "Healthcare personnel" OR "Healthcare profession” OR "Healthcare professions” OR "Health care provider" OR "Health care providers" OR "Health care practitioner" OR "Health care practitioners" OR "Health care worker" OR "Health care workers" OR "Health care personnel" OR "Health care profession” OR "Health care professions” OR “Medical professional” OR “Medical professionals” OR “Medical personnel” OR “Medical provider” OR “Medical providers” OR “Medical practitioner” OR “Medical practitioners” OR “Medical profession” OR “Medical professions” OR "Emergency Medicine Technicians" OR "Emergency Medicine Technician" OR Paramedic* OR dentist* OR dental* OR Endodont* OR pedodont* OR paedodont* OR orthodont* OR Periodont* OR Prosthodont* OR Nurs* OR Pharmac* OR Doctor* OR Physician* OR Clinician* OR “Occupational therapist” OR Anesthesiologist* OR Anesthetist* OR Anaesthetist* OR Anaesthesiologist* OR Cardiologist* OR Dermatologist* OR Endocrinologist* OR Gastroenterologist* OR Hepatologist* OR “General Practitioner” OR “General Practitioners” OR Geriatrician* OR Gerontologist* OR Gynecologist* OR Gynaecologist* OR Hospitalist* OR internist* OR Nephrologist* OR Neurologist* OR Obstetrician* OR Orthopedic*OR Orthopaedic* OR Oncologist* OR Ophthalmologist* OR Osteopath* OR Otologist* OR Otolaryngologist* OR Pathologist* OR Pediatrician* OR Paediatrician* OR Physiatrist* OR Psychatrist* OR Pulmonologist* OR Radiologist* OR Rheumatologist* OR Surgeon* OR Urologist* OR “Occupational therapists” OR “Physical therapist” OR “Physical therapists” OR Physiotherapist*) AND (DE "Arabs" OR MA qatar OR bahrain OR oman OR "Saudi arabia" OR saudi OR Kuwait OR Arabia* OR "united arab emirates" OR yemen OR egypt OR jordan OR lebanon OR syria OR Iraq OR libya OR morocco OR tunisia OR djibouti OR sudan OR "South sudan" OR pakistan OR "North africa" OR "northern africa" OR "east africa" OR "eastern africa" OR bedouin OR maghreb OR maghrib OR "gulf cooperation council" OR GCC OR SU qatar OR bahrain OR oman OR "Saudi arabia" OR saudi OR Kuwait OR Arabia* OR "united arab emirates" OR yemen OR egypt OR jordan OR lebanon OR syria OR Iraq OR Iran OR Afghanistan OR Somalia OR libya OR morocco OR tunisia OR djibouti OR sudan OR "South sudan" OR pakistan OR "North africa" OR "northern africa" OR "east africa" OR "eastern africa" OR bedouin OR maghreb OR maghrib OR "gulf cooperation council" OR GCC) OR (TX UAE OR TX U.A.E OR TX Emirat* OR TX United Arab Emirates OR TX Qatar* OR TX Oman* OR TX Saudi Arabia* OR TX Saudi* OR TX Kuwait* OR TX Bahrain* OR TX Yemen* OR TX Egypt* OR TX Jordan* OR TX Leban* OR TX Syria* OR TX Iraq* OR TX West Bank OR TX Gaza* OR TX Palestin* OR TX Iran* OR TX Somali* OR TX Afghan* OR TX Libya* OR TX Morocc* OR TX Tunis* OR TX Djibouti* OR TX Sudan* OR TX South Sudan* OR TX Pakistan* OR TX North Africa* OR TX North-Africa* OR TX "Northern Africa" OR TX "East Africa" OR TX "Eastern Africa" OR TX Maghreb OR TX Maghrib OR TX Arab* OR TX Bedouin* OR TX "Gulf Cooperation Council" OR TX GCC OR TX "Middle East") OR (TX UAE OR TX U.A.E OR TX Emirat* OR TX United Arab Emirates OR TX Qatar* OR TX Oman* OR TX Saudi Arabia* OR TX Saudi* OR TX Kuwait* OR TX Bahrain* OR TX Yemen* OR TX Egypt* OR TX Jordan* OR TX Leban* OR TX Syria* OR TX Iraq* OR TX West Bank OR TX Gaza* OR TX Palestin* OR TX Libya* OR TX Morocc* OR TX Tunis* OR TX Djibouti* OR TX Sudan* OR TX South Sudan* OR TX Pakistan* OR TX North Africa* OR TX North-Africa* OR TX "Northern Africa" OR TX "East Africa" OR TX "Eastern Africa" OR TX Maghreb OR TX Maghrib OR TX Arab* OR TX Bedouin* OR TX "Gulf Cooperation Council" OR TX GCC OR TX "Middle East") AND (DE "Major Depression" OR DE "Anaclitic Depression" OR DE "Dysthymic Disorder" OR DE "Endogenous Depression" OR DE "Late Life Depression" OR DE "Postpartum Depression" OR DE "Reactive Depression" OR DE "Recurrent Depression" OR DE "Treatment Resistant Depression") OR (DE "Depression (Emotion)")) OR (DE "Bipolar Disorder" OR DE "Bipolar I Disorder" OR DE "Bipolar II Disorder" OR DE "Cyclothymic Disorder" OR DE "Mania")) OR (DE "Mania" OR DE "Hypomania")) OR (DE "Affective Disorders" OR DE "Disruptive Mood Dysregulation Disorder" OR DE "Major Depression" OR DE "Seasonal Affective Disorder")) OR (DE "Adjustment Disorders") OR DE "Affective Disorders" OR DE "Disruptive Mood Dysregulation Disorder" OR DE "Major Depression" OR DE "Seasonal Affective Disorder" OR TX (Depressi* OR Dysthymi* OR Bipolar OR Mani* OR "Seasonal affective disorder" OR Melanchol* OR "Adjustment disorder" OR coping OR cope OR cyclothymi* OR "Internalizing symptom" OR "Internalizing symptoms" OR dysphor* OR "Reactive disorder" OR "Mood dysregulation" OR "mood disorder" OR "mood disorders" OR Affective psychos?s) | All 3 concepts Limiters - Population Group: Human; Book Type: Conference Proceedings Search modes - Boolean/Phrase | 10 | 16-Oct-19 |
| DE "Health Personnel" or DE "Paramedics" OR TX("medical staff" or "healthcare staff" or "health care staff" OR "Healthcare provider" OR "Healthcare providers" OR "Healthcare practitioner" OR "Healthcare practitioners" OR "Healthcare worker" OR "Healthcare workers" OR "Healthcare personnel" OR "Healthcare profession” OR "Healthcare professions” OR "Health care provider" OR "Health care providers" OR "Health care practitioner" OR "Health care practitioners" OR "Health care worker" OR "Health care workers" OR "Health care personnel" OR "Health care profession” OR "Health care professions” OR “Medical professional” OR “Medical professionals” OR “Medical personnel” OR “Medical provider” OR “Medical providers” OR “Medical practitioner” OR “Medical practitioners” OR “Medical profession” OR “Medical professions” OR "Emergency Medicine Technicians" OR "Emergency Medicine Technician" OR Paramedic* OR dentist* OR dental* OR Endodont* OR pedodont* OR paedodont* OR orthodont* OR Periodont* OR Prosthodont* OR Nurs* OR Pharmac* OR Doctor* OR Physician* OR Clinician* OR “Occupational therapist” OR Anesthesiologist* OR Anesthetist* OR Anaesthetist* OR Anaesthesiologist* OR Cardiologist* OR Dermatologist* OR Endocrinologist* OR Gastroenterologist* OR Hepatologist* OR “General Practitioner” OR “General Practitioners” OR Geriatrician* OR Gerontologist* OR Gynecologist* OR Gynaecologist* OR Hospitalist* OR internist* OR Nephrologist* OR Neurologist* OR Obstetrician* OR Orthopedic*OR Orthopaedic* OR Oncologist* OR Ophthalmologist* OR Osteopath* OR Otologist* OR Otolaryngologist* OR Pathologist* OR Pediatrician* OR Paediatrician* OR Physiatrist* OR Psychatrist* OR Pulmonologist* OR Radiologist* OR Rheumatologist* OR Surgeon* OR Urologist* OR “Occupational therapists” OR “Physical therapist” OR “Physical therapists” OR Physiotherapist*) AND (DE "Arabs" OR MA qatar OR bahrain OR oman OR "Saudi arabia" OR saudi OR Kuwait OR Arabia* OR "united arab emirates" OR yemen OR egypt OR jordan OR lebanon OR syria OR Iraq OR libya OR morocco OR tunisia OR djibouti OR sudan OR "South sudan" OR pakistan OR "North africa" OR "northern africa" OR "east africa" OR "eastern africa" OR bedouin OR maghreb OR maghrib OR "gulf cooperation council" OR GCC OR SU qatar OR bahrain OR oman OR "Saudi arabia" OR saudi OR Kuwait OR Arabia* OR "united arab emirates" OR yemen OR egypt OR jordan OR lebanon OR syria OR Iraq OR Iran OR Afghanistan OR Somalia OR libya OR morocco OR tunisia OR djibouti OR sudan OR "South sudan" OR pakistan OR "North africa" OR "northern africa" OR "east africa" OR "eastern africa" OR bedouin OR maghreb OR maghrib OR "gulf cooperation council" OR GCC) OR (TX UAE OR TX U.A.E OR TX Emirat* OR TX United Arab Emirates OR TX Qatar* OR TX Oman* OR TX Saudi Arabia* OR TX Saudi* OR TX Kuwait* OR TX Bahrain* OR TX Yemen* OR TX Egypt* OR TX Jordan* OR TX Leban* OR TX Syria* OR TX Iraq* OR TX West Bank OR TX Gaza* OR TX Palestin* OR TX Iran* OR TX Somali* OR TX Afghan* OR TX Libya* OR TX Morocc* OR TX Tunis* OR TX Djibouti* OR TX Sudan* OR TX South Sudan* OR TX Pakistan* OR TX North Africa* OR TX North-Africa* OR TX "Northern Africa" OR TX "East Africa" OR TX "Eastern Africa" OR TX Maghreb OR TX Maghrib OR TX Arab* OR TX Bedouin* OR TX "Gulf Cooperation Council" OR TX GCC OR TX "Middle East") OR (TX UAE OR TX U.A.E OR TX Emirat* OR TX United Arab Emirates OR TX Qatar* OR TX Oman* OR TX Saudi Arabia* OR TX Saudi* OR TX Kuwait* OR TX Bahrain* OR TX Yemen* OR TX Egypt* OR TX Jordan* OR TX Leban* OR TX Syria* OR TX Iraq* OR TX West Bank OR TX Gaza* OR TX Palestin* OR TX Libya* OR TX Morocc* OR TX Tunis* OR TX Djibouti* OR TX Sudan* OR TX South Sudan* OR TX Pakistan* OR TX North Africa* OR TX North-Africa* OR TX "Northern Africa" OR TX "East Africa" OR TX "Eastern Africa" OR TX Maghreb OR TX Maghrib OR TX Arab* OR TX Bedouin* OR TX "Gulf Cooperation Council" OR TX GCC OR TX "Middle East") AND (DE "Major Depression" OR DE "Anaclitic Depression" OR DE "Dysthymic Disorder" OR DE "Endogenous Depression" OR DE "Late Life Depression" OR DE "Postpartum Depression" OR DE "Reactive Depression" OR DE "Recurrent Depression" OR DE "Treatment Resistant Depression") OR (DE "Depression (Emotion)")) OR (DE "Bipolar Disorder" OR DE "Bipolar I Disorder" OR DE "Bipolar II Disorder" OR DE "Cyclothymic Disorder" OR DE "Mania")) OR (DE "Mania" OR DE "Hypomania")) OR (DE "Affective Disorders" OR DE "Disruptive Mood Dysregulation Disorder" OR DE "Major Depression" OR DE "Seasonal Affective Disorder")) OR (DE "Adjustment Disorders") OR DE "Affective Disorders" OR DE "Disruptive Mood Dysregulation Disorder" OR DE "Major Depression" OR DE "Seasonal Affective Disorder" OR TX (Depressi* OR Dysthymi* OR Bipolar OR Mani* OR "Seasonal affective disorder" OR Melanchol* OR "Adjustment disorder" OR coping OR cope OR cyclothymi* OR "Internalizing symptom" OR "Internalizing symptoms" OR dysphor* OR "Reactive disorder" OR "Mood dysregulation" OR "mood disorder" OR "mood disorders" OR Affective psychos?s) | All 3 concepts Limiters - Document Type: Abstract Collection Search modes - Boolean/Phrase | 0 | 16-Oct-19 |
| DE "Health Personnel" or DE "Paramedics" OR TX("medical staff" or "healthcare staff" or "health care staff" OR "Healthcare provider" OR "Healthcare providers" OR "Healthcare practitioner" OR "Healthcare practitioners" OR "Healthcare worker" OR "Healthcare workers" OR "Healthcare personnel" OR "Healthcare profession” OR "Healthcare professions” OR "Health care provider" OR "Health care providers" OR "Health care practitioner" OR "Health care practitioners" OR "Health care worker" OR "Health care workers" OR "Health care personnel" OR "Health care profession” OR "Health care professions” OR “Medical professional” OR “Medical professionals” OR “Medical personnel” OR “Medical provider” OR “Medical providers” OR “Medical practitioner” OR “Medical practitioners” OR “Medical profession” OR “Medical professions” OR "Emergency Medicine Technicians" OR "Emergency Medicine Technician" OR Paramedic* OR dentist* OR dental* OR Endodont* OR pedodont* OR paedodont* OR orthodont* OR Periodont* OR Prosthodont* OR Nurs* OR Pharmac* OR Doctor* OR Physician* OR Clinician* OR “Occupational therapist” OR Anesthesiologist* OR Anesthetist* OR Anaesthetist* OR Anaesthesiologist* OR Cardiologist* OR Dermatologist* OR Endocrinologist* OR Gastroenterologist* OR Hepatologist* OR “General Practitioner” OR “General Practitioners” OR Geriatrician* OR Gerontologist* OR Gynecologist* OR Gynaecologist* OR Hospitalist* OR internist* OR Nephrologist* OR Neurologist* OR Obstetrician* OR Orthopedic*OR Orthopaedic* OR Oncologist* OR Ophthalmologist* OR Osteopath* OR Otologist* OR Otolaryngologist* OR Pathologist* OR Pediatrician* OR Paediatrician* OR Physiatrist* OR Psychatrist* OR Pulmonologist* OR Radiologist* OR Rheumatologist* OR Surgeon* OR Urologist* OR “Occupational therapists” OR “Physical therapist” OR “Physical therapists” OR Physiotherapist*) AND (DE "Arabs" OR MA qatar OR bahrain OR oman OR "Saudi arabia" OR saudi OR Kuwait OR Arabia* OR "united arab emirates" OR yemen OR egypt OR jordan OR lebanon OR syria OR Iraq OR libya OR morocco OR tunisia OR djibouti OR sudan OR "South sudan" OR pakistan OR "North africa" OR "northern africa" OR "east africa" OR "eastern africa" OR bedouin OR maghreb OR maghrib OR "gulf cooperation council" OR GCC OR SU qatar OR bahrain OR oman OR "Saudi arabia" OR saudi OR Kuwait OR Arabia* OR "united arab emirates" OR yemen OR egypt OR jordan OR lebanon OR syria OR Iraq OR Iran OR Afghanistan OR Somalia OR libya OR morocco OR tunisia OR djibouti OR sudan OR "South sudan" OR pakistan OR "North africa" OR "northern africa" OR "east africa" OR "eastern africa" OR bedouin OR maghreb OR maghrib OR "gulf cooperation council" OR GCC) OR (TX UAE OR TX U.A.E OR TX Emirat* OR TX United Arab Emirates OR TX Qatar* OR TX Oman* OR TX Saudi Arabia* OR TX Saudi* OR TX Kuwait* OR TX Bahrain* OR TX Yemen* OR TX Egypt* OR TX Jordan* OR TX Leban* OR TX Syria* OR TX Iraq* OR TX West Bank OR TX Gaza* OR TX Palestin* OR TX Iran* OR TX Somali* OR TX Afghan* OR TX Libya* OR TX Morocc* OR TX Tunis* OR TX Djibouti* OR TX Sudan* OR TX South Sudan* OR TX Pakistan* OR TX North Africa* OR TX North-Africa* OR TX "Northern Africa" OR TX "East Africa" OR TX "Eastern Africa" OR TX Maghreb OR TX Maghrib OR TX Arab* OR TX Bedouin* OR TX "Gulf Cooperation Council" OR TX GCC OR TX "Middle East") OR (TX UAE OR TX U.A.E OR TX Emirat* OR TX United Arab Emirates OR TX Qatar* OR TX Oman* OR TX Saudi Arabia* OR TX Saudi* OR TX Kuwait* OR TX Bahrain* OR TX Yemen* OR TX Egypt* OR TX Jordan* OR TX Leban* OR TX Syria* OR TX Iraq* OR TX West Bank OR TX Gaza* OR TX Palestin* OR TX Libya* OR TX Morocc* OR TX Tunis* OR TX Djibouti* OR TX Sudan* OR TX South Sudan* OR TX Pakistan* OR TX North Africa* OR TX North-Africa* OR TX "Northern Africa" OR TX "East Africa" OR TX "Eastern Africa" OR TX Maghreb OR TX Maghrib OR TX Arab* OR TX Bedouin* OR TX "Gulf Cooperation Council" OR TX GCC OR TX "Middle East") AND (DE "Major Depression" OR DE "Anaclitic Depression" OR DE "Dysthymic Disorder" OR DE "Endogenous Depression" OR DE "Late Life Depression" OR DE "Postpartum Depression" OR DE "Reactive Depression" OR DE "Recurrent Depression" OR DE "Treatment Resistant Depression") OR (DE "Depression (Emotion)")) OR (DE "Bipolar Disorder" OR DE "Bipolar I Disorder" OR DE "Bipolar II Disorder" OR DE "Cyclothymic Disorder" OR DE "Mania")) OR (DE "Mania" OR DE "Hypomania")) OR (DE "Affective Disorders" OR DE "Disruptive Mood Dysregulation Disorder" OR DE "Major Depression" OR DE "Seasonal Affective Disorder")) OR (DE "Adjustment Disorders") OR DE "Affective Disorders" OR DE "Disruptive Mood Dysregulation Disorder" OR DE "Major Depression" OR DE "Seasonal Affective Disorder" OR TX (Depressi* OR Dysthymi* OR Bipolar OR Mani* OR "Seasonal affective disorder" OR Melanchol* OR "Adjustment disorder" OR coping OR cope OR cyclothymi* OR "Internalizing symptom" OR "Internalizing symptoms" OR dysphor* OR "Reactive disorder" OR "Mood dysregulation" OR "mood disorder" OR "mood disorders" OR Affective psychos?s) | All 3 concepts Limiters - Population Group: Human; Document Type: Dissertation Search modes - Boolean/Phrase | 69 | 16-Oct-19 |
| DE "Health Personnel" or DE "Paramedics" OR TX("medical staff" or "healthcare staff" or "health care staff" OR "Healthcare provider" OR "Healthcare providers" OR "Healthcare practitioner" OR "Healthcare practitioners" OR "Healthcare worker" OR "Healthcare workers" OR "Healthcare personnel" OR "Healthcare profession” OR "Healthcare professions” OR "Health care provider" OR "Health care providers" OR "Health care practitioner" OR "Health care practitioners" OR "Health care worker" OR "Health care workers" OR "Health care personnel" OR "Health care profession” OR "Health care professions” OR “Medical professional” OR “Medical professionals” OR “Medical personnel” OR “Medical provider” OR “Medical providers” OR “Medical practitioner” OR “Medical practitioners” OR “Medical profession” OR “Medical professions” OR "Emergency Medicine Technicians" OR "Emergency Medicine Technician" OR Paramedic* OR dentist* OR dental* OR Endodont* OR pedodont* OR paedodont* OR orthodont* OR Periodont* OR Prosthodont* OR Nurs* OR Pharmac* OR Doctor* OR Physician* OR Clinician* OR “Occupational therapist” OR Anesthesiologist* OR Anesthetist* OR Anaesthetist* OR Anaesthesiologist* OR Cardiologist* OR Dermatologist* OR Endocrinologist* OR Gastroenterologist* OR Hepatologist* OR “General Practitioner” OR “General Practitioners” OR Geriatrician* OR Gerontologist* OR Gynecologist* OR Gynaecologist* OR Hospitalist* OR internist* OR Nephrologist* OR Neurologist* OR Obstetrician* OR Orthopedic*OR Orthopaedic* OR Oncologist* OR Ophthalmologist* OR Osteopath* OR Otologist* OR Otolaryngologist* OR Pathologist* OR Pediatrician* OR Paediatrician* OR Physiatrist* OR Psychatrist* OR Pulmonologist* OR Radiologist* OR Rheumatologist* OR Surgeon* OR Urologist* OR “Occupational therapists” OR “Physical therapist” OR “Physical therapists” OR Physiotherapist*) AND (DE "Arabs" OR MA qatar OR bahrain OR oman OR "Saudi arabia" OR saudi OR Kuwait OR Arabia* OR "united arab emirates" OR yemen OR egypt OR jordan OR lebanon OR syria OR Iraq OR libya OR morocco OR tunisia OR djibouti OR sudan OR "South sudan" OR pakistan OR "North africa" OR "northern africa" OR "east africa" OR "eastern africa" OR bedouin OR maghreb OR maghrib OR "gulf cooperation council" OR GCC OR SU qatar OR bahrain OR oman OR "Saudi arabia" OR saudi OR Kuwait OR Arabia* OR "united arab emirates" OR yemen OR egypt OR jordan OR lebanon OR syria OR Iraq OR Iran OR Afghanistan OR Somalia OR libya OR morocco OR tunisia OR djibouti OR sudan OR "South sudan" OR pakistan OR "North africa" OR "northern africa" OR "east africa" OR "eastern africa" OR bedouin OR maghreb OR maghrib OR "gulf cooperation council" OR GCC) OR (TX UAE OR TX U.A.E OR TX Emirat* OR TX United Arab Emirates OR TX Qatar* OR TX Oman* OR TX Saudi Arabia* OR TX Saudi* OR TX Kuwait* OR TX Bahrain* OR TX Yemen* OR TX Egypt* OR TX Jordan* OR TX Leban* OR TX Syria* OR TX Iraq* OR TX West Bank OR TX Gaza* OR TX Palestin* OR TX Iran* OR TX Somali* OR TX Afghan* OR TX Libya* OR TX Morocc* OR TX Tunis* OR TX Djibouti* OR TX Sudan* OR TX South Sudan* OR TX Pakistan* OR TX North Africa* OR TX North-Africa* OR TX "Northern Africa" OR TX "East Africa" OR TX "Eastern Africa" OR TX Maghreb OR TX Maghrib OR TX Arab* OR TX Bedouin* OR TX "Gulf Cooperation Council" OR TX GCC OR TX "Middle East") OR (TX UAE OR TX U.A.E OR TX Emirat* OR TX United Arab Emirates OR TX Qatar* OR TX Oman* OR TX Saudi Arabia* OR TX Saudi* OR TX Kuwait* OR TX Bahrain* OR TX Yemen* OR TX Egypt* OR TX Jordan* OR TX Leban* OR TX Syria* OR TX Iraq* OR TX West Bank OR TX Gaza* OR TX Palestin* OR TX Libya* OR TX Morocc* OR TX Tunis* OR TX Djibouti* OR TX Sudan* OR TX South Sudan* OR TX Pakistan* OR TX North Africa* OR TX North-Africa* OR TX "Northern Africa" OR TX "East Africa" OR TX "Eastern Africa" OR TX Maghreb OR TX Maghrib OR TX Arab* OR TX Bedouin* OR TX "Gulf Cooperation Council" OR TX GCC OR TX "Middle East") AND (DE "Major Depression" OR DE "Anaclitic Depression" OR DE "Dysthymic Disorder" OR DE "Endogenous Depression" OR DE "Late Life Depression" OR DE "Postpartum Depression" OR DE "Reactive Depression" OR DE "Recurrent Depression" OR DE "Treatment Resistant Depression") OR (DE "Depression (Emotion)")) OR (DE "Bipolar Disorder" OR DE "Bipolar I Disorder" OR DE "Bipolar II Disorder" OR DE "Cyclothymic Disorder" OR DE "Mania")) OR (DE "Mania" OR DE "Hypomania")) OR (DE "Affective Disorders" OR DE "Disruptive Mood Dysregulation Disorder" OR DE "Major Depression" OR DE "Seasonal Affective Disorder")) OR (DE "Adjustment Disorders") OR DE "Affective Disorders" OR DE "Disruptive Mood Dysregulation Disorder" OR DE "Major Depression" OR DE "Seasonal Affective Disorder" OR TX (Depressi* OR Dysthymi* OR Bipolar OR Mani* OR "Seasonal affective disorder" OR Melanchol* OR "Adjustment disorder" OR coping OR cope OR cyclothymi* OR "Internalizing symptom" OR "Internalizing symptoms" OR dysphor* OR "Reactive disorder" OR "Mood dysregulation" OR "mood disorder" OR "mood disorders" OR Affective psychos?s) | All 3 concepts Limiters - Population Group: Human; Document Type: Journal Article Search modes - Boolean/Phrase | 2,127 | 16-Oct-19 |
| Searches 1 or 2 or 3 or 4 or 5 or 6 |  |  |  |

**Additional Material 4: Characteristics of included studies on depression in healthcare workers in the EMR**

| **Included Study Citation** | **Country** | **Study Population** | **Setting** | **Study Design** | **Year of Data Collection** | **Sampling Method** | **Sample Size** | **Age** | **Sex** | **Instrument** | **Conflict of Interest** | **Funding Information** |
| --- | --- | --- | --- | --- | --- | --- | --- | --- | --- | --- | --- | --- |
| Abbas et al., 2012 [3] | Saudi Arabia | Nurses | All hospitals and centers inside King Fahad Medical City | Cross-sectional | . | Unclear | 715 | Mean age: 35.2 ± 8.2 | M: 83 (11.6%) F: 632 (88.4%) | Hospital Anxiety and Depression Scale (HADS) | Not reported | Not reported |
| Abdulrahman et al., 2018 [4] | United Arab Emirates | Medical residents | Healthcare centers in the emirates of Dubai and Abu Dhabi | Cross-sectional | May - December 2016 | Unclear | 302 | ≤25: 26 (9%) >25: 275 (91%) | M: 63 (21%) F: 236 (79%) | Patient Health Questionnaire (PHQ-9) | No conflict | None |
| Ahmed et al., 2009 [5] | United Arab Emirates | Medical staff | 3 government hospitals, 3 primary health care centers and Dubai Medical College (DMC) in UAE. | Cross-sectional | November 2008 | Unclear | 93 | . | . | Beck Depression Inventory (BDI-2) | Not reported | Not reported |
| Al Ghailani et al., 2018 [6] | United Arab Emirates | Residents | Six institutions (PHCCs & Hospitals) in Abu Dhabi | Cross-sectional | 2016 | Unclear | 296 | . | M: 63 (21.3%) F: 233 (78.8%) | de novo questionnaire | No conflict | None |
| Al-Amer et al. , 2020 [7] | Jordan | Nurses | Different health sectors | Cross-sectional | March 22 - 27, 2020 | Non-probability based sampling | 405 | Mean age was 30.27 years ± 8.38 | M: 28.6% F: 71.4% | Depression Anxiety Stress Scale (DASS-21), Arabic version | No conflict | Not reported |
| Al-Ghafri et al., 2014 [8] | Oman | Medical residents | Enrolled in an Oman Medical Specialty Board (OMSB) training programs | Cross-sectional | . | Non-probability based sampling | 132 | 25–30: 96 >30: 36 | M: 42 (33%) F: 90 (67%) | Patient Health Questionnaire (PHQ-9) | COI reported: Dr. Samir Al-Adawi is a member of the World Health Organization Expert Consultation Group on Feeding and Eating Disorders, reporting to the International Advisory Group for the Revision of ICD-10 Mental and Behavioral Disorders. The views expressed in this article are those of the authors, and not the official policies or positions of the Consultation Group, the Advisory Group, or of the World Health Organization. | None |
| Al-Hammad et al., 2012 [9] | Saudi Arabia | Nurses | Inpatient wards of King Khalid University Hospital | Cross-sectional | . | . | 281 | Mean: 35 years Range: 20-50 | M: 25 (9%) F: 256 (91%) | General Health Questionnaire-28 (GHQ-28) | Not reported | Not reported |
| Al-Houqani et al., 2020 [10] | Oman | Residents | All Oman Medical Specialty Board Residents | Cross-sectional | January - March 2017 | Non-probability based sampling | 399 | Mean: 29.1±1.9  Range: 25-31 | M:39.6% F: 60.4% | Patient Health Questionnaire (PHQ-9) | No conflict | None |
| Al-Hussein et al., 2010 [11] | Iraq | Nurses | 7 teaching hospitals in Mosul city | Cross-sectional | October 2006 - August 2007 | Probability based sampling | 250 | 20-30: 160 >30-40: 55 >40: 35 | M: 210  F: 40 | Depression Anxiety Stress Scale (DASS-42) | Not reported | Not reported |
| Al-Maddah et al., 2015 [12] | Saudi Arabia | Medical residents | King Fahd University Hospital (KFUH) | Cross-sectional | February - April 2012 | . | 171 | 25-30: (80%) 30-35: (16%) | M: 72 (42%) F: 99 (58%) | Beck Depression Inventory (BDI-2) | No conflict | Not reported |
| Al-Zahrani et al., 2014 [13] | Saudi Arabia | Physicians | Governmental ER hospitals in Makkah | Cross-sectional | . | Probability based sampling | 100 | Mean age of depressed: 36.6 Mean age of non-depressed:35.9 | M: 68 F: 32 | Beck Depression Inventory (BDI-2) | No conflict | None |
| Albajjar et al., 2019 [14] | Saudi Arabia | Interns | College of Medicine of Albaha University in Albaha city | Cross-sectional | 2017–2018 | Non-probability based sampling | 21 | Mean age: 22.03 ± 1.94  Age range: 19 and 26 years | M | Beck Depression Inventory (BDI-2) | No conflict | None |
| AlFahhad, 2018 [15] | Saudi Arabia | Healthcare workers | King Abdul Aziz Medical City (KAMC) Health Affair, Ministry of National Guard | Cross-sectional | September 2015 - August 2016 | Unclear | 300 | Mean age: 33±8.6 Range:22-62 | M: 99 (33%) F: 201 (67%) | Patient Health Questionnaire (PHQ-9) | No conflict | None |
| AlFaris et al., 2019 [16] | Saudi Arabia | Residents | 6 major specialties (family medicine, internal medicine, obstetrics and gynecology, general surgery, pediatrics and psychiatry) in King Saud University Medical City (KSUMC), Riyadh | Cross-sectional | 2017–2018 | Probability based sampling | 186 | Mean age: 26.9 | M: 87 (46.8%) F: 99 (53.2%) | Beck Depression Inventory (BDI-2) | The authors declare that they have no competing interests. The co-author GP is an Associate Editor of this journal. | None |
| AlGhamdi et al., 2020 [17] | Saudi Arabia | Healthcare workers | Web-based population survey | Cross-sectional | May 6 - May 13, 2020 | Non-probability based sampling | 428 | . | . | Depression Anxiety Stress Scale (DASS-21), English & Arabic versions | No conflict | None |
| AlHarby, 2009 [18] | Saudi Arabia | Physicians | PHCC in Ministry of Health at Jeddah Governorate | Cross-sectional | . | Unclear | 150 | . | M + F | Beck Depression Inventory (BDI-2) | Unknown | Unknown |
| Alhifzi et al., 2018 [19] | Saudi Arabia | Emergency physicians | 9 major hospitals in Riyadh, Saudi Arabia, | Case control | November 2015 - April 2016. | Non-probability based sampling | 137 | 35.2±8.6 | M: 48 (70.6%) F: 20 (29.4%) | Beck Depression Inventory (BDI-2) | No conflict | This study was supported by a grant from the Strategic Technologies Program of the National Plan for Sciences and Technology and Innovation in the Kingdom of Saudi Arabia (08-MED511-02). |
| Alipoor et al., 2015 [20] | Iran | Nurses | Fasa University of Medical Sciences | Cross-sectional | 2014 | Non-probability based sampling | 142 | Mean age: 31.5±8.6 | . | Depression Anxiety Stress Scale (DASS-21) | Unknown | Unknown |
| Alkhazrajy et al., 2014 [21] | Iraq | Health care providers | 5 primary health care centers at Alkadhmiya district | Cross-sectional | February - June 2012 | Unclear | 364 | Mean age: 38.937±10.754 | M: 187 F: 177 | DSM-IV-TR | Not reported | Not reported |
| Almutairi et al., 2020 [22] | Saudi Arabia | Paramedics / EMTs | Saudi Red Crescent Authority (SRCA) stations in Riyadh, Saudi Arabia | Cross-sectional | March-June 2017 | Unclear | 240 | <25yrs: 60 (25.0%) 25‐34yrs: 146 (60.8%)  35‐44yrs: 23 (9.6%)  45‐54yrs: 10 (4.2%) ≥55yrs: 1 (0.4) | M: 100% | Depression Anxiety Stress Scale (DASS-21) | No conflict | None |
| Alshardi et al., 2019 [23] | Saudi Arabia | Medical residents | King Abdulaziz Medical City (KAMC), Ministry of National Guard Health Affairs, Jeddah (tertiary care hospital) | Cross-sectional | . | Unclear | 149 | ≤ 26: 76 (51.0%)  > 26: 73 (49.0%) | M: 70 (47.0%) F: 79 (53.0%) | Patient Health Questionnaire (PHQ-9) | No conflict | Not reported |
| Andejani et al., 2017 [24] | Saudi Arabia | Plastic surgery residents | Saudi Plastic Surgery Program in Riyadh | Cross-sectional | 2nd half of April 2017 | Non-probability based sampling | 34 | <30: 73% | M: 19 F: 15 | Beck Depression Inventory (BDI-2) | No conflict | Not reported |
| Ansaripour et al., 2016 [25] | Iran | Healthcare workers | Health care homes of Isfahan University of Medical Science | Descriptive-analytic study of correlation type | 2013 | Non-probability based sampling | 297 | . | . | Patient Health Questionnaire (PHQ-9) | Unknown | Unknown |
| Ardekani et al., 2008 [26] | Iran | Nurses | All 12 hospitals in Shiraz | Cross-sectional | September -October 2006 | Probability based sampling | 1195 | Mean age: 30.81±7.08 | M: 131 (10.96%) F: 1064 (89.04%) | General Health Questionnaire-28 (GHQ-28) | Not reported | University grant |
| Arefian et al., 2009 [27] | Iran | Nurses | ICUs & CCUs of 3 Shahid Beheshti University hospitals | Cross-sectional | Summer & Autumn 2006 | Non-probability based sampling | 199 | 20 to>=40 | M+F | Beck Depression Inventory (BDI-2) | Not reported | Not reported |
| Ariapooran, 2019 [28] | Iran | Nurses | 3 hospitals in Malayer | Cross-sectional | January -March 2017 | Non-probability based sampling | 247 | Mean age: 31.03±5.44 | M: (45.7%) F: (54.3%) | Beck Depression Inventory 13 (BDI-13) | No conflict | University grant |
| Asad Zandi et al., 2011 [29] | Iran | Nurses | Military hospital, Tehran | Cross-sectional | February 2008-April 2009 | Non-probability based sampling | 272 | M: 38.69±7.8  F: 35.53±4.93 | M: 43% F: 57% | Depression Anxiety Stress Scale (DASS-21) | Not reported | Not reported |
| Ashraf et al., 2019 [30] | Pakistan | Medical doctors | General ward, Emergency and OPD of three hospitals of Islamabad/ Rawalpindi | Correlational study | June-September 2018 | Non-probability based sampling | 157 | . | M: 40% F: 60% | Depression Anxiety Stress Scale (DASS-21) | No conflict | None |
| Aslam et al., 2013 [31] | Pakistan | Postgraduate doctors | Medical and surgical wards Services hospital, Lahore | Cross-sectional | Jan 2012 | Non-probability based sampling | 195 | Mean age: 27 Range: 24-38 | M: 127 F: 64 | Hospital Anxiety and Depression Scale (HADS) | Not reported | Not reported |
| Atif et al., 2016 [32] | Pakistan | Doctors | Combined Military Hospital, Lahore | Cross-sectional | Feb 2014 to Jan 2015 | Probability based sampling | 97 | Mean age: 33.47±9.454 | M:52 (53.6%)  F: 45 (46.4%) | Hospital Anxiety and Depression Scale (HADS) | No conflict | Not reported |
| Badahdah et al., 2020 [33] | Oman | Healthcare workers (315 nurses + 194 physicians) | Web-based survey of 10 multiple healthcare facilities | Cross-sectional | First 2 weeks of April 2020 | Probability based sampling | 509 | Mean age: 37.67 years ± 7.68 | M: 19.7% F: 80.3% | World Health Organization Perceived Well-Being Index (WHO-5) | Not reported | None |
| Bana et al., 2019 [34] | Pakistan | Dental Surgeons | Karachi | Cross-sectional | April-June 2017 | Probability based sampling | 234 | 24-30: 121  31-40: 81 >41: 32 | M: 113 (48.3%) F: 121 (51.7%) | Aga Khan University Anxiety and Depression Scale (AKUADS) | Not reported | Not reported |
| Behnam et al., 2016 [35] | Iran | Nurses | Semnan University of Medical Sciences hospitals | Cross-sectional | 2014 | Unclear | 182 | Mean age: 29.74±7.919 | M: 44 (24.2%) F: 138 (75.8%) | Beck Depression Inventory (BDI-2) | Unknown | Unknown |
| Bukhari et al., 2016 [36] | Pakistan | Nurses | Federal government tertiary care hospital in Islamabad | Cross-sectional | . | Non-probability based sampling | 250 | 25-40 | F | Aga Khan University Anxiety and Depression Scale (AKUADS) | Not reported | Not reported |
| Bukhari et al., 2019 [37] | Pakistan | Nurses | Federal government tertiary care hospital in Islamabad | Cross-sectional | . | Non-probability based sampling | . | 25-40 | F | Aga Khan University Anxiety and Depression Scale (AKUADS) | Not reported | Not reported |
| Dachraoui et al., 2017 [38] | Tunisia | Medical and allied health staff | 5 ICUs in the university hospital Fattouma Bourguiba Monastir Tunisia | Cross-sectional | September 2016 | Unclear | 106 | Median age: 32 ±8.4 | M:42% F: 58% | Hospital Anxiety and Depression Scale (HADS) | Not reported | Not reported |
| Darawad, 2009 [39] | Jordan | Nurses | ICUs of the Jordan University Hospital (JUH) | Cross-sectional | . | Probability based sampling | 114 | Average: 27.1±4.6 Range: 22-46 | M: 42 (36.8%) F: 72 (63.2%) | Center for Epidemiologic Studies Depression Scale (CES-D) | Not reported | Not reported |
| Dehghan et al., 2012 [40] | Iran | Rural health workers | Larestan | Cross-sectional | 2010 | Unclear | 99 | . | . | Beck Depression Inventory (BDI-2) | No conflict | Funded by Larestan health center |
| Dehghani et al., 2009 [41] | Iran | Nurses | Namazi Hospital of Shiraz University of Medical Sciences | Cross-sectional | 2008 | Unclear | 311 |  |  | Zung Self-Rating Depression Scale | Unknown | Unknown |
| El Kissi et al., 2014 [42] | Tunisia | Nurses | Sousse Farhat Hached teaching hospital (CHU), Sousse | Cross-sectional | . | Non-probability based sampling | 228 | Mean: 41.5±8.6 Range: 24-58 | M: 102 (44.7%) F: 126 (55.3%) | Composite International Diagnostic Interview (CIDI) sections related to MDD (Tunisian version) | Not reported | Not reported |
| El-Hamrawy et al., 2018 [43] | Egypt | Doctors and nurses | A tertiary healthcare hospital and a PHCC in Shibin El-Kom city, Menoufia governorate | Cross-sectional | February - December, 2016 | Non-probability based sampling | 1646 | Mean age: 35.5 ± 9.37 | M+F | Patient Health Questionnaire (PHQ-9) | No conflict | None |
| Fahim et al., 2018 [44] | Egypt | Medical and surgical oncology residents | Suez Canal University Hospital | Cross-sectional | . | Unclear | 37 | . | . | Symptom Checklist for Depression (SCD) | Unknown | Unknown |
| Farag et al., 2019 [45] | Egypt | Residents | Ain Shams University Hospital | Cross-sectional | June 2018 -January 2019 | Unclear | 220 | Mean: 1.013 ± 26.968 Range: 23-30 | M: 43.64% F: 56.36% | Beck Depression Inventory | Unknown | Unknown |
| Farahani et al., 2017 [46] | Iran | Nurses | Khorramabad Hospitals | . | 2016 | Unclear | 241 | . | . | Beck Depression Inventory (BDI-2) | Unknown | Unknown |
| Ghazwin et al., 2016 [47] | Iran | Nurses | Emergency wards in 3 teaching hospitals affiliated to Tehran ‎University of Medical Sciences | Cross-sectional | July - December 2014 | Unclear | 94 | . | M: 29 (30.9%) F: 65 (69.1%) | Depression Anxiety Stress Scale (DASS-21) | No conflict | Not reported |
| Habibi et al., 2014 [48] | Iran | Nurses | ORs of Shahid Rajaie Hospitals, Ghazvin | . | 2012 | Unclear | 80 | 35.7±5.4 | F | Beck Depression Inventory (BDI-2) | Unknown | Unknown |
| Halayem-Dhouib S et al., 2010 [49] | Tunisia | Nurses | Razi Psychiatric hospital | Cross-sectional | . | Unclear | 54 | . | . | Beck Depression Inventory (BDI-2) | Unknown | Unknown |
| Halvani et al., 2012 [50] | Iran | Nurses | 3 medical centers | Descriptive analytical | . | Unclear | 150 | Range: 23-50 Mean: 31.1±6.7 | M+F | Beck Depression Inventory (BDI-2) | Not reported | None |
| Haqqi, 2013 [51] | Pakistan | Residents | 3 campuses of Ziauddin Hospital | . | . | Unclear | 86 | . | M+F | Beck Depression Inventory | No conflict | None |
| Hasan et al., 2018 [52] | Egypt | Nurses | Psychiatric hospital, Port Said | Cross-sectional | January - July 2016 | Unclear | 70 | ≤20 yrs: 14.2% 20–30 yrs: 57.1% 31–40 yrs: 22.8% ≥40 yrs: 5.0% | M: 26 (37.1%) F: 44 (62.9%) | Beck Depression Inventory (BDI-2) | No conflict | Not reported |
| Hassannia et al., 2020 [53] | Iran | Doctors, nurses & health staff | Online | Cross-sectional | April 6 to April 15, 2020 | Probability based sampling | 461 | . | . | Hospital Anxiety and Depression Scale (HADS) | No conflict | This study was supported and funded by Mazandaran University of Medical Sciences. |
| Ibrahim et al., 2016 [54] | Saudi Arabia | Nurses | King Abdulaziz University Hospital, Jeddah | Cross-sectional | 2014-2015 | Non-probability based sampling | 226 | Mean age: 36.5±9.2 Range: 21 to 60 | M: 18 (7.9%) F: 211 (92.1%) | Hospital Anxiety and Depression Scale (HADS) | No conflict | None |
| Ibrahim et al., 2019 [55] | Saudi Arabia | Expat nurses | Government healthcare facilities (20 hospitals and 2 primary healthcare clinics) in Al-Qassim | Cross-sectional | January - June 2017 | Non-probability based sampling | 977 | Mean age: 32 ± 7 | F: 99.4% | Depression Anxiety Stress Scale (DASS-21) | No conflict | This work was supported by Qassim University (Buraydah, Saudi Arabia), represented by the Deanship of Scientific Research, [grant number 1536-med-2016-1-12-S] during the academic year 1437 AH/ 2016 AD. It had no role in the study design, the collection, analysis, interpretation of data, the writing of the report, or in the decision to submit the article for publication. |
| Jabeen et al., 2020 [56] | Pakistan | Healthcare workers (nurses + doctors) | Wah Medical College and POF Hospital, Wah Cantt | Cross-sectional | May 1 - May 7, 2020 | Non-probability based sampling | 340 | Mean age: 40.65 ± 11.64 years | M: 35.9% F: 64.1% | Depression Anxiety Stress Scale (DASS-21) | No conflict | None |
| Kamimura et al., 2018 [57] | Iraq | Physicians | Includes solo practice, small clinics, large clinics, hospitals and academic practice | Online through one physician’s professional network + snowballing | October-December 2017 | Probability based sampling | 120 | Mean: 29.99 (SD=5.51) | M: 45% F: 55% | Patient Health Questionnaire (PHQ-9) | No conflict | This study was partially supported by the College of Social and Behavioral Science, University of Utah. |
| Kashani et al., 2017 [58] | Iran | Nurses | Emergency departments of 3 hospitals, Shohadaye Tajrish, Imam Hossein and Loghmane Hakim, Tehran | Cross-sectional | 2015 | Non-probability based sampling | 102 | Mean age: 30.2 ± 5.6 Age range: 23-49 | F | Minnesota Multiphasic Personality Inventory-2 (MMPI-2) test | Not reported | This study was conducted with a grant from the Clinical Re- search Development Center of Loghmane Hakim Hospital. This study was a part of Dr. Sahar Mirbaha’s thesis for Emergency Medicine Residency at Shahid Beheshti University of Medical Sciences, Tehran, Iran. |
| Kassani et al, 2014 [59] | Iran | Nurses | Ilam city hospitals | Cross-sectional | 2011 | Unclear | . | . | . | Beck Depression Inventory | Unknown | Unknown |
| Kassani et al., 2015 [60] | Iran | Nurses | 6 Ilam hospitals | Cross-sectional | 2013 | Probability based sampling | 191 | 20 - 30: 31.94% 30 - 40: 51.31% >40: 16.75% | M: 38.74% F: 61.26% | Beck Depression Inventory (BDI-2) | Not reported | The study was a research project supported financially by the research center for prevention of socio-psychological injuries of Ilam university of medical sciences (grant No. 201/52/22). |
| Kavari et al., 2007 [61] | Iran | Nurses | Shiraz Namazi Hospital | Cross-sectional | 2006 | Probability based sampling | 130 | . | . | Beck Depression Inventory (BDI-2) | Not reported | Not reported |
| Kazemi et al., 2010 [62] | Iran | Nurses | Military hospital, Tehran | . | 2010 | Probability based sampling | 335 | 31.4±7.5 | . | Beck Depression Inventory (BDI-2) | Unknown | Unknown |
| Khalid et al., 2010 [63] | Pakistan | Nurses | Abbasi Shaheed Hospital | Cross-sectional | . | Probability based sampling | 70 | 25-29: 44% 30-34: 33% 35-40: 23% | F | Hamilton Rating Depression Scale (HAM-D) | No conflict | Not reported |
| Khalilzadeh et al., 2005 [64] | Iran | Nurses | Urmia University of Medical Sciences | . | 2005 | Non-probability based sampling | 200 |  |  | Beck Depression Inventory (BDI-2) | Unknown | Unknown |
| Khamseh et al., 2011 [65] | Iran | Nurses | Educational hospitals affiliated to a university, Tehran | Cross-sectional | . | Non-probability based sampling | 413 | 30-40 |  | Depression Anxiety Stress Scale (DASS-21) | Unknown | Unknown |
| Khan et al., 2020 [66] | Saudi Arabia | Paramedics / EMTs | Saudi Red Crescent Authority (SRCA), Makkah district | Cross-sectional | November 2018-February 2019 | Unclear | 104 | 32.5±6.1 | M | Beck Depression Inventory (BDI-2) | No conflict | Not reported |
| Khani et al., 2016 [67] | Iran | Nurses | Hospitals affiliated to Neyshabur University of Medical Sciences | Cross-sectional | 2014 | Non-probability based sampling | 196 | Mean age: 32.1 |  | Beck Depression Inventory (BDI-2) | Unknown | Unknown |
| Khodadadi et al., 2016 [68] | Iran | Nurses | Tabriz hospital | Cross-sectional | . | Non-probability based sampling | 242 | Mean age: 33.13±6.1 | M: 57 (23.5%) F: 185 (76.5%) | Depression Anxiety Stress Scale (DASS-21) | Not reported | Not reported |
| Kousha et al., 2018 [69] | Iran | Resident physicians | University of Medical Science, Rasht, Iran | Cross-sectional | 2014-2015 | Unclear | 100 | <30: 48 ≥30: 52 | M: 26 F: 74 | Depression Anxiety Stress Scale (DASS-21) | No conflict | This research was conducted as a residency thesis and has been supported by our University of Medical Sciences and Grant No: 90320104. |
| Koushali et al., 2013 [70] | Iran | Nurses | selected hospitals of Medical Sciences University in Tehran | Cohort, but initial stage (prior to Ramadan fasting) can be considered cross-sectional | 2010 | Non-probability based sampling | 313 | 37.82±7.17 | M: 177 (56.5%) F: 136 (43.5%) | Depression Anxiety Stress Scale (DASS-21) | No conflict | Religion and Medicine Research Centre and Nursing Department of Baqiyatallah University of Medical Sciences |
| Lafta et al., 2016 [71] | Iraq | Junior doctors | 20 major teaching hospitals in Baghdad | Cross-sectional | July-August 2016 | Non-probability based sampling | 323 | 24-39 (mean 29.5+ 3.8) | M: 55.1% F: 44.9% | Center for Epidemiologic Studies Depression Scale (CES-D) | Not reported | Not reported |
| Maghrabi et al., 2019 [72] | Saudi Arabia | Physicians (Consultants, specialists & residents) | 3 governmental hospitals, and its linked Primary Health Care Centers and 2 private hospitals, Jeddah | Cross-sectional | . | Non-probability based sampling | 288 | Mean: 36.9 | M: 36.8% F: 63.2% | Patient Health Questionnaire (PHQ-9) | Not reported | None |
| Mahmodi et al., 2013 [73] | Iran | Nurses | Beasat, Tohid and Gods hospital in Sanandaj city | Cross-sectional | January - May 2009 | Non-probability based sampling | 314 | . | M: 95 F: 219 | Beck Depression Inventory (BDI-2) | Unknown | Unknown |
| Mahmoudi et al., 2009 [74] | Iran | Nurses | Education hospitals in Mazandaran townships | Cross-sectional | 2005 | Non-probability based sampling | 504 | <30:34.3% 30-40: 37.7% >40: 28% | M: 41.9% F: 58.1% | Beck Depression Inventory (BDI-2) | Not reported | Not reported |
| Malik et al., 2017 [75] | Bahrain | Primary care providers (family physicians and GPs) | All public sector local healthcare centers in governorates of Bahrain | Cross-sectional | .June 2014 | Non-probability based sampling | 210 | < 35: 23.8% 35-50: 60.0% > 50: 16.2% | M: 21.9% F: 78.1% | Depression Anxiety Stress Scale (DASS-21) | Not reported | Not reported |
| Mami et al., 2014 [76] | Iran | Nurses | State hospitals in Ilam | Cross-sectional | . | Unclear | 117 | . | . | Beck Depression Inventory (BDI-2) | Unknown | Unknown |
| Marzouk et al., 2018 [77] | Tunisia | Medical residents | Faculty of Medicine of Tunis | Cross-sectional | 14-22 December 2015 | Non-probability based sampling | 1700 | Mean age: 28.5±2 Range: 27-30 | M: 39.2% F: 60.8% | Hospital Anxiety and Depression Scale (HADS) | No conflict | None |
| Mehdi et al., 2020 [78] | Pakistan | Health care professionals (doctors, nurses & paramedics) | Sahiwal Medical College, Sahiwa using online questionnaire | Cross-sectional | . | Unclear | 237 | Range: 20 - >50 | M: 43.1% F: 56.9% | Hospital Anxiety and Depression Scale (HADS) | No conflict | None |
| Mirmohammadi et al., 2009 [79] | Iran | Nurses | Shahid Sadoughi University Hospitals, Yazd | Cross-sectional | 2009 | Unclear | 110 | 38.46±5.48 | . | Beck Depression Inventory (BDI-2) | Unknown | Unknown |
| Mobasher et al., 2015 [80] | Egypt | Intern doctors | Kasr Al-Ainy Hospitals | Cross-sectional | March - June 2013 | Non-probability based sampling | 300 | . | M: 149 (49.7%) F: 151 (50.3%) | Beck Depression Inventory (BDI-2) | No conflict | Not reported |
| Mogharab et al., 2016 [81] | Iran | Nurses | Critical care units and emergency rooms of Valiasr and Imam Reza hospitals, Birjand, | Descriptive, analytical study | 2015 | Non-probability based sampling | 109 | . | M: 17.8% F: 82.2% | Beck Depression Inventory (BDI-2) | Not reported | Not reported |
| Mohammed et al., 2014 [82] | Egypt | Resident physicians | Suez Canal University Hospital | Cross-sectional | . | Non-probability based sampling | 84 | . | M: 46 (54.8%) | Beck Depression Inventory (BDI-2) | No conflict |  |
| Mohammed et al., 2019 [83] | Sudan | Healthcare providers (doctors, nurses, pharmacists, medical assistants, midwives and technicians) | Governmental health facilities (18 health centers + 1 teaching hospital) in Alhasaheesa locality, Al Gezira state | Cross-sectional | October 2016-July 2017 | Non-probability based sampling | 208 | 20-29: 139 (66.8%) 30-39: 37 (17.8%) 40-49: 23 (11.1%) 50-59: 9 (4.3%) | M: 85 (40.9%) F: 123 (59.1%) | Hospital Anxiety and Depression Scale (HADS) | Not reported | Not reported |
| Momeni et al., 2016 [84] | Iran | Emergency medicine residents and specialist | Different educational hospitals in Tehran | Cross-sectional | . | Non-probability based sampling | 204 | 26-30: 46 (22.5%) 31-35: 82 (40.2%) 36-40: 47 (23%) 41-45: 23 (11.3%)  ≥45: 6 (2.9%) | M: 146 (71.6%)  F: 58 (28.4%) | General Health Questionnaire-28 (GHQ-28) | No conflict | Not reported |
| Monsef et al., 2015 [85] | United Arab Emirates | Residents | Dubai Health Authority | Cross-sectional | April 2012-February 2013 | Unclear | 169 | 21-26: 51 (30.18) 26-30: 110 (65.09) >30: 8 (4.73) | M: 24 (14.2) F: 145 (85.8) | Depression Anxiety Stress Scale (DASS-21) | Not reported | Not reported |
| Mustahsan et al., 2013 [86] | Pakistan | House officers and postgraduate trainees | 1 private and 4 public tertiary care hospitals in Karachi | Cross-sectional | February - May 2012 | Unclear | 364 | . | M: 90 (24.72%) F: 274 (75.27%) | Clinical diagnosis by doctors | Not reported | Not reported |
| Naser et al., 2020 [87] | Jordan | Healthcare providers | Online | Cross-sectional | March 22-28, 2020 | Probability based sampling | 1163 | 18- >50 | M: 43.9% F: 56.1% | Patient Health Questionnaire (PHQ-9) | No conflict | This study was supported by Isra University (Amman, Jordan). |
| Nisar et al., 2012 [88] | Pakistan | Doctors | Armed Forces Postgraduate Medical Institute Rawalpindi | Cross-sectional | June - Dec 2010 | Non-probability based sampling | 100 | . | . | Beck Depression Inventory (BDI-2), Urdu version | Not reported | Not reported |
| Nooli et al., 2017 [89] | Saudi Arabia | Medical Interns | King Khalid University, Abha | Cross-sectional | . | Unclear | 49 | . | M | Patient Health Questionnaire (PHQ-9) | No conflict | None |
| Pournamdarian et al., 2012 [90] | Iran | Nurses | Be’sat hospital in Hamadan. | Cross-sectional | . | Non-probability based sampling | 120 |  |  | Depression Anxiety Stress Scale (DASS-21) | Unknown | Unknown |
| Poursadeghiyan et al., 2016 [91] | Iran | Nurses | Different cities in Iran | Cross-sectional | . | Unclear | 250 | Mean age: 36.4±7.69 Age range: 24-61 years | . | Beck Depression Inventory (BDI-2) | Not reported | Not reported |
| Raessi et al., 2015 [92] | Iran | Nurses | Ganjavian Dezfool hospital | Correlational study | . | Unclear | . | . | . | General Health Questionnaire-28 (GHQ-28) | Unknown | Unknown |
| Raffah et al., 2013 [93] | Saudi Arabia | Physicians + Dentists | 30 PHCCs of ministry of health in Makkah al-Mukarramah | Cross-sectional | September 2013 | Non-probability based sampling | 90 | Mean age: 43.42±9.60 | M: 71.1% F: 28.9% | Beck Depression Inventory (BDI-2) | Not reported | Not reported |
| Rahmati et al., 2019 [94] | Iran | First-year emergency medicine residents | Shahid Beheshti University of Medical Sciences | Cross-sectional | 2014-2015 | Non-probability based sampling | 99 | Mean age: 33.93±5.92  Age range: 26 – 55 | M: 43.4% F: 56.6% | Minnesota Multiphasic Personality Inventory-2 (MMPI-2) test | No conflict | None |
| Raza et al., 2017 [95] | Pakistan | Doctors (House officers, trainee medical officers & consultants) | 3 Public and private tertiary care hospitals of Peshawar | Cross-sectional | April-September 2015 | Non-probability based sampling | 320 | Range: 22-54 | M: 67.2% F: 32.8% | Beck Depression Inventory (BDI-2) | No conflict | Not reported |
| Roughani et al., 2019 [96] | Iran | Nurses | Imam Khomeini Hospital and Martyr Mostafa Khomeini Hospital in Ilam | Cross-sectional | . | Non-probability based sampling | 110 | Mean age: 30.26 ± 8.25 | M: 37.3% F: 62.7% | Beck Depression Inventory (BDI-2) | No conflict | Not reported |
| Sadeghi et al., 2007 [97] | Iran | Residents | Hospitals of 3 medical universities in Tehran | Cross-sectional | . | Unclear | 2251 | . | M/F | Beck Depression Inventory (BDI-2) | Unknown | Unknown |
| Shabany et al., 2018 [98] | Iran | Nurses | Tehran University of Medical Sciences | Cross-sectional | August 2015 - August 2016 | Non-probability based sampling | 113 | Mean age: 25.3±0.5 | M: 66 (58.4%)  F: 47 (41.5%) | Beck Depression Inventory (BDI-2) | Not reported | Not reported |
| Sheikh et al., 2018 [99] | Pakistan | Senior doctors | Teaching hospitals in cities of Lahore and Faisalabad | Cross-sectional | January - February, 2017 | Probability based sampling | 268 | 45-50: 239 (89.2%)  50-60: 29 (10.8%) | M: 142 (53%) F: 126 (47%) | Hospital Anxiety and Depression Scale (HADS) | No conflict | None |
| Taghinejad et al., 2014 [100] | Iran | Nurses | 3 hospitals, affiliated to Iranian health ministry, in Ilam city (western Iran) | Cross-sectional | . | Probability based sampling | 81 | Mean age: 32.77 ± 6.06 | M: 49.4% F: 50.6% | General Health Questionnaire-28 (GHQ-28) | No conflict | Not reported |
| Taghva et al., 2014 [101] | Iran | Nurses | 5 different hospital departments of AJA hospitals, Tehran | Cross-sectional | 2012 | Non-probability based sampling | 173 | Mean age: 45±6.63 |  | Beck Depression Inventory (BDI-2) | Unknown | Unknown |
| Tajvar et al., 2015 [102] | Iran | ICU nurses | Shahid Mohammadi Hospital in Bandar Abbas City | Cross-sectional | 2013 | Non-probability based sampling | 56 | Range: 23 to 45 Average: 31.57±5.86 | F | General Health Questionnaire-28 (GHQ-28) | No conflict | Not reported |
| Talih et al., 2016 [103] | Lebanon | Residents and interns | All specialties at the American University of Beirut Medical Center | Cross-sectional | August - October 2013 | Unclear | 118 | 18-25: 26 (22 %)  26-35: 92 (78 %) | M: 62 (53 %)  F: 56 (47 %) | Patient Health Questionnaire (PHQ-9) | No conflict | Not reported |
| Talih et al., 2018 [104] | Lebanon | Nurses | Academic medical center in Beirut | Cross-sectional | September - October 2013 | Non-probability based sampling | 91 | < 25: 25 (27.4%) 26 - 35: 52 (57.1%) 36 - 65: 14 (15.3%) | M: 39 (42.8%)  F: 52 (57.1%) | Patient Health Questionnaire (PHQ-9) | Not reported | Not reported |
| Vahedian-Azimi et al., 2020 [105] | Iran | Medical staff | Medical staff treating Covid-19 patients of Baqiyatallah Hospital, Tehran | Cross-sectional | At least a week February-March 2020 | Unclear | 217 | Mean: 39.57±6.71 Range: 28-62 | M: 51.2% F: 48.8% | Depression Anxiety Stress Scale (DASS-21) | No conflict | None |
| Yasemi et al., 2014 [106] | Iran | Nurses | ICUs & ORs of Ilam hospitals | Cross-sectional | 2013 | Non-probability based sampling | 60 | Mean age: 31.86±8.35 | M: 52.1% F: 47.9% | Beck Depression Inventory (BDI-2) | Unknown | Unknown |
| Younesi et al., 2017 [107] | Iran | Hospital staff | Gilan Province | Cross-sectional | 2012 | Non-probability based sampling | 57 | Mean age: 31.5 Age Range: 23-43 | M: 11% F: 89% | General Health Questionnaire-28 (GHQ-28) | No conflict | None |
| Yousuf et al., 2011 [108] | Pakistan | Residents & fellows | Aga Khan University Hospital (AKUH), Karachi, Pakistan | Cross-sectional | June-August 2008 | Probability based sampling | 172 | <30: 70.3% ≥30: 29.7% Not responded: 14.0% | M: 64.5% F: 35.5% | Zung Self-Rating Depression Scale | Not reported | Not reported |
| Zaher et al., 2016 [109] | Iran | Nurses with at least 1 year experience | Critical care and internal-surgical units in the selected hospitals of social security organization of Tehran | Cross-sectional | March-October-2016 | Non-probability based sampling | 220 | Mean age: 34.92 | M: 38.6% F: 61.4% | Depression Anxiety Stress Scale (DASS-42) | Not reported | Not reported |
| Zaki et al., 2016 [110] | Egypt | Nurses | Mansoura University Hospitals | Cross-sectional | Oct 2013 - June 2014 | Probability based sampling | 150 | Range: 20-35 | . | Hospital Anxiety and Depression Scale (HADS) | Not reported | Not reported |

No conflict: Authors reported no conflict of interest

Not reported: Authors failed to report conflict of interest

None: Authors reported no source of funding

Unknown: Full texts were in Farsi or could not be retrieved, so we are unable to determine if authors reported conflicts of interest / funding

**Additional Material 5: Risk / Protective Factors and Recommendations**

| **Included Study** | **Country** | **Risk & Protective Factors** | **Recommendations** | **Limitations** |
| --- | --- | --- | --- | --- |
| Abbas et al., 2012 [3] | Saudi Arabia | We couldn't find statistically significant association between both overtime work and night shift and the manifestations of depression in our study we found that nursing staff who are smokers are more likely to have depression symptoms | Major health education and health promotion programs are required to foster exercise and no smoking Culture among nursing staff. Annual HAD scale might be helpful in identifying nursing staff who are considered as probable clinical | . |
| Abdulrahman et al., 2018 [4] | United Arab Emirates | Significant differences in depression was not observed among the subspecialties (P = 0.7) Marital status affected depression among the medical residents (P = 0.07) (Singles more depressed) Residents who did not smoke (P = 0.0008), found job satisfaction (P = 0.001), never had thoughts of quitting the residency program (P = 0.001), and received emotional support from their family and friends (P = 0.002) were significantly less likely to be depressed as compared to their counterparts | . | . |
| Al Ghailani et al., 2018 [6] | United Arab Emirates | Spending <8h in pleasurable activities and depression (p=0.005) | Our reported numbers are much lower than those reported from the USA in 2006, in which 65% of the residents reported working for >80 h a week on an average. This difference can be explained by better regulation of duty hour requirements and violations in recent years. However, program directors should be more aggressive while dealing with duty hour violations.  Our reported numbers are much lower than those reported from the USA in 2006, in which 65% of the residents reported working for >80 h a week on an average.[11] This difference can be explained by better regulation of duty hour requirements and violations in recent years. However, program directors should be more aggressive while dealing with duty hour violations. | Seeking to assess twenty residency programs in six institutes may produce heterogeneous results as the challenges and specific problems may be different in different locations and different programs. However, there remains some common issues that could be teased out by this generic study. It is a cross‐sectional study, thus only giving an assessment at one time point but does not allow how to ascertain outcomes of how residents dealt with challenges and obstacles. |
| Al-Amer et al., 2020 [7] | Jordan | A significant positive relationship was found between depression and gender (r = 0.200; p < 0.01); number of children (r = 0.206; p < 0.01); and dealing with persons with COVID-19 (r = 0.180; p < 0.01). Multiple linear regression analysis: The full model that contained all predictors of depression was statistically significant (F (3, 216) = 11.801; p < 0.001). Findings showed that gender, number of children, and dealing with person with COVID-19 were the main significant predictors of depression [(B = 0.176; p < 0.01), (B = 0.232; p < 0.01), (B = 0.255; p < 0.01)]. Meanwhile, dealing with a person with COVID-19 was the strongest predictor, which appeared to be a serious factor for depression (partial = 0.264, part = 0.254). | . | Utilized a cross- sectional design, some limitations were observed. This study used self-reporting questionnaires, which is well recognized in the research realm. However, it increases social desirability biases. Furthermore, cross-sectional studies cannot determine the cause and effect between the study variables but, rather, will only report associations between study variables. |
| Al-Ghafri et al., 2014 [8] | Oman | The rate of depression was not statistically associated with gender, age, or residency level | . | . |
| Al-Hammad et al., 2012 [9] | Saudi Arabia |  | Coping strategies such as workshops, counseling group meetings have to be scheduled in order to decrease physiological and psychological problems related with shiftwork. Nursing staff's free time can be arranged so as to facilitate recovery from shift work, especially after night shift. | We did not determine the effect of specialty on the level of psychosocial stress and health of nurses. Although this was done in one of the biggest multicultural nursing settings in Riyadh, still the results cannot be generalized to all the hospital settings of KSA. |
| Al-Houqani et al., 2020 [10] | Oman | Being female (p=0.030), not engaging regularly in physical activity (p=0.019) and extremes of sleep (p=0.001) A senior resident is more likely to suffer any kind of depression (33% vs 19.6%), but a junior resident is more likely to suffer severe depression (3.5% vs 1.5%) (p=0.006) | Health education and promotion is recommended to increase awareness of the benefits of early screening and diagnosis to prevent a major form of depression and to address the progression to harmful effects of depression on the quality of life and academic performance. Further research on the consequences of depression and its effects is needed in this area and to highlight. | The actual prevalence of depression might be overestimated because the self-reporting nature of this study may have resulted in recall bias and over- reporting of the symptoms. Besides, other factors associated with depression need to be addressed as they could be an important confounder that may affect and lead to depression, such as chronic diseases and major life events. even though the PHQ-9 is a well-known and validated screening tool, it cannot be used alone for diagnosing and managing depression. It should be followed by proper clinical evaluation and assessment. |
| Al-Hussein et al., 2010 [11] | Iraq | . | Development of nurses and sub-medical personnel, relationship, communications with patient's family and community; financially, psychologically and legally may be undertaken. Pay more attention to the nursing staff regarding the work-related aspects. | . |
| Al-Maddah et al., 2015 [12] | Saudi Arabia | Statistically significant association between the number of working hours per night duty and depressive symptoms (P = 0.003) and working hours per week and depressive symptoms (P = 0.047) Association between acute sleep deprivation and depressive symptoms among residents was statistically significant (P = 0.009), with 45.9% of acutely sleep deprived residents showing mild depressive symptoms and 21.2% reporting moderate to severe depressive symptoms. The percentage of female residents showing moderate to severe depressive symptoms was more than double that of the male residents. No significant associations between chronic sleep deprivation and depressive symptoms among residents (P = 0.115) No significant associations between depressive symptoms and sociodemographic factors, with the exception of gender (F>M, p = 0.033) No significant relationship was found between the prevalence of depressive symptoms and choice of specialty or level of residency | A clear detailed guideline be set stating the number of working hours and night duties for residents. Specific guidelines for pregnant female residents should also be included. These new guidelines should be assessed on a regular basis by further studies. These new guidelines should be assessed on a regular basis by further studies. Additionally, the authors recommend that all medical residents be educated on the importance of sleep and its relationship to depression. Residents should be capable of predicting symptoms of sleep deprivation and depression and amend their sleeping patterns accordingly. Furthermore, a well-trained mentor for each resident is necessary to help minimize stress in the professional setting. Regular meetings should also be organized for all residents, including newly graduated specialists, in order for them to share their knowledge and experiences with one another. | A few residents faced difficulties in answering the questions that depended on recalling their frequency of sleep. Furthermore, self-reported values may have been biased or inaccurate. Certain residents did not respond due to either a lack of time or interest and therefore were not included in the study. As the study was cross-sectional, it was difficult to determine causality. The simple random sampling technique was difficult to apply due to the limited time frame of the study and the number of residents in the study population. Finally, potential comorbidities and medications among the residents were not evaluated, which may have affected the results. |
| Al-Zahrani et al., 2014 [13] | Saudi Arabia | Age, marital status, income level, physical activity, presence of chronic disease were not statistically correlated to risk of depression 31.6% of physicians who were performing recreational activities had depression compared to 56.5% of physicians who were not performing it. This difference was statistically significant (p value <0.013). 75% of physicians who were smoker had depression compared to 25% of physicians who were not smoker. These differences were statistically significant p<0.05. 3% of physicians who had recent or previous history of depression found to be depressed compared to 42% of physicians who were not. This difference was statistically highly significant p value<0.008. | The main recommendations to high authority are to give more care to recreational activity for physicians, healthy lifestyle particularly smoking cessation through a planned program, frequent assessment of stressor facing ER physicians which may predispose to depression. Finally, screening, diagnosis and treatment of depression among ER physicians seems of help to them. Furthermore, enough support to ER physicians suffering from depression through arrangement of follow up visit to prevent relapse | . |
| AlFahhad, 2018 [15] | Saudi Arabia | Singles had a higher rate of initial diagnosis of depression (6.3%) while married had 1.9% which was statistically significant with a p-value 0.05. Also, singles had a higher major depressive disorder (9.9%) while married had 3.8% with a p-value of 0.03 The mean of age of workers who had major depressive symptoms was 28.4 years with SD of 6.3 years and p-value (0.03) which was statistically significant. The relation between age and depression was (−0.2) which is negatively strongly correlated with a p-value (0.00), and the relation between depression score and the years of experience was (−0.1) which is negatively strongly related with a p-value (0.00), and it is statistically significant Differences in depression due to gender, job type, working hours, shift work, but these differences were statistically insignificant | Addressing the prevalence of depression and associated risk factors will help reduce this disorder among the working population that will improve productivity. It is recommended to conduct an optional annual screening of depression among HCWs and to design the working hours and the shifts schedule in a way that balances the sleep cycle and social life with work life. | . |
| AlFaris et al., 2019 [16] | Saudi Arabia | The rate of depression was higher among the female residents than their male counterparts , and the difference was statistically significant (p = 0.007) Statistically significant association between the training year and the severity of depression, the senior residents had more severe depression compared to their junior counterparts (p = 0.026). Senior residents are more likely to report depressive symptoms than their junior counterparts There was a statistically significant moderate negative correlation between BDI-II score and the SSI total score (r = − 0.447, p < 0.001) | Implementing a course for a high level of study skills among residents may help them to improve their mental health. This is especially so, if later studies find that poor study skills are one of the contributing factors for depressive symptoms among residents | The low reliability of the BDI II could be explained by the relatively small sample size in this study. As this study is correlational in nature, causality cannot be claimed. Also, since the study did not take into account the training culture and educational environments of the residents, how these factors impact on depressive symptoms and study skills could not be elucidated. Since multiple factors affect both study skills and depression, this study should be viewed as an investigation of two multi-factorial constructs, without controlling for the other known and unknown factors. another limitation is this study was conducted in a single institution, which may reduce the generalizability of the results. Therefore, we need a multi-center prospective study to provide a better understanding of this topic. |
| AlGhamdi et al., 2020 [17] | Saudi Arabia | . | First, this was not a comparative study with simultaneous prospective data collection from different countries; therefore, the data cannot be used to draw rm conclusions about the effect of curfew regulations on psychological burden during pandemics. Second, we used a convenience sample. This may have resulted in selection bias: individuals with very low or very high levels of anxiety may have refrained from participating in the study because they avoid accessing the news media on which the study tool was disseminated. Third, the design was sufficiently powerful to detect major differences in psychological distress scores, but may not have been sufficiently powerful to detect small differences between some subgroups (such as older adults, who constituted a relatively small proportion of our sample). Fourth, the sample was mostly drawn from the western region of the KSA, which may limit the generalizability of the findings to the rest of the country, let alone to other countries. | First, this was not a comparative study with simultaneous prospective data collection from different countries; therefore, the data cannot be used to draw rm conclusions about the effect of curfew regulations on psychological burden during pandemics. Second, we used a convenience sample. This may have resulted in selection bias: individuals with very low or very high levels of anxiety may have refrained from participating in the study because they avoid accessing the news media on which the study tool was disseminated. Third, the design was sufficiently powerful to detect major differences in psychological distress scores, but may not have been sufficiently powerful to detect small differences between some subgroups (such as older adults, who constituted a relatively small proportion of our sample). Fourth, the sample was mostly drawn from the western region of the KSA, which may limit the generalizability of the findings to the rest of the country, let alone to other countries. |
| AlHarby, 2009 [18] | Saudi Arabia | Females (53.3%) significantly more than males (28.6%)  Prevalence higher among physicians who are not performing physical exercise or recreational activity  Young age or recently graduated | Make physicians aware about RF predisposing to depression and try to enjoy their life as general population does | . |
| Alhifzi et al., 2018 [19] | Saudi Arabia | Difference between cases and controls was statistically significant (p<0.02) There was a strong correlation between emergency physicians with poor sleep quality (i.e., scores of ≥5 on PSQI) and depressive symptoms as assessed by the BDI scores (r = 0.437, P < 0.001). | Several coping strategies have been described to prevent fatigue and insomnia among shift workers. These strategies may include scheduled napping, exposure to light at work, and special nutrition guidelines such as taking caffeine, especially at the start of a night shift. Scheduled short napping which minimizes sleep inertia has generally been shown to be helpful. Bright light is a promising effective method to reduce both fatigue and insomnia. However, there is still a lack of standardized recommendations for light therapy. It is proposed that effective use of light may result in an adaptation to the extended night shift period and enhance the subsequent readaptation to daytime activities Whenever possible, shift schedules should be in a manner consistent with circadian principles that rotate in a clockwise manner (day to evening to night). In addition, when possible, long shifts on consecutive nights should be avoided. Moreover, the ACEP recommends scheduling isolated night shifts or relatively long sequences of night shifts, and it also advocates that shift duration should be no longer than 12 h, there should be regular scheduling of at least 24 h off-duty and a place must be provided to sleep before driving home after night shifts.[14] Future call schedules should consider the total number of hours worked by each physician and the intervals of time off between shifts. To modify the circadian rhythm and adapt to the new shift, the ACEP stresses that physicians should have regularly scheduled periods of at least 24 h off work. Night shift workers’ schedules need to be meticulously designed to provide for planned sleep periods. A critical point that needs clear attention of emergency physicians and the management of emergency departments is providing a place to sleep before driving home after night shifts. | This study has a few limitations. First, the proportion of male participants is higher than female participants. Thus, the results cannot be generalizable to both sexes. Second, the number of participants in both groups was relatively small. Nevertheless, the findings may simulate a larger national study to assess the impact of shift work on emergency physicians. Third, shift time and frequency were not accounted for in the questionnaire, which are parameters that may affect the degree of sleep deprivation and its consequences. |
| Alipoor et al., 2015 [20] | Iran | Significant relationship between age and depression was found (P=0.006). But there wasn’t any significant relationship between depression and level of education, gender and marital status. | Considering the important role of nurses in improvement and promotion of public health, elimination of underlying factors that cause emotional reactions in nursing must be addressed, as a health priority | . |
| Alkhazrajy et al., 2014 [21] | Iraq | Statistically significant association (p=0.001) between depression and age >50 yrs: 77.8%  Statistically significant relationship between depression and number of children among medical participants being high percent of depression (82%)who had between(3-5 )child No significant association between depression and gender, educational level, marital status, monthly income, years of experience, monthly income or presence of chronic illnesses demographic data | Increase awareness of primary health care providers about depressive symptoms. Improvement of the economic status of the Iraqi families. Screening for depressive symptoms should be considered to primary health care employee with chronic diseases, marital status (divorced and widowed) and those with large families. Adapt suitable environment at primary health care centers | A convenience sample were taken but short time interview with each participants due to preoccupation with work because of overcrowding of patients that will not gave us a very precise estimate of the questionnaire. Furthermore, it was hard to involve all PHCC included within AlKadhmiya district because of limited period the research was taken. |
| Almutairi et al., 2020 [22] | Saudi Arabia | Depression was reported to be significantly associated with taking medications for NCDs (P = 0.050) and sleeping >8 h/day (P = 0.007). In terms of depression, respondents on medications for NCDs in the last 3 months were at almost fourfold higher risk for depression than those without a medication history (AOR = 4.43; 95 CI = 1.24–15.79), while those with 6–8 h of sleep/day were at almost 60% lower risk for depression than those with <6 h sleep/day (AOR = 0.42; 95 CI = 0.20–0.89) [Multivariate regression analysis] | . | It should be noted that the generalizability of this finding is limited to on‑field emergency medical professionals of SRCA, Riyadh, as other health‑care professions were not included. A limitation of this study is that due to the cross‑sectional design, a temporal relationship between risk factors and the outcome could not be assessed. In addition, the sample of this study is very specific; this limits its direct comparisons with other studies and also its consequent cumulative effect. Despite those limitations, the study could have a public health importance in exploring this important 2020issue in Saudi Arabia, which also can impact the quality of care delivered to emergency patients. |
| Alshardi et al., 2019 [23] | Saudi Arabia | Being unmarried, having a chronic medical condition, and being at a higher residency level (R4) were significant risk factors for depression (mild–severe) (OR 2.81, 95% CI 1.13, 7.01; OR 9.45, 95% CI 1.12, 80.05; OR 6.14, 95% CI 1.31, 28.93; respectively) Being in an emergency program was associated with a higher risk of severe depression (p = .01) Residents in surgery and emergency programs were at a higher risk for moderate/severe depression (OR 2.60, 95% CI 1.13, 5.98; OR 4.90, 95% CI 1.08, 22.20; respectively) Medical residents in higher years showed a lower risk for severe depression, although a statistical difference was only observed among R3 residents compared to R1(p= .046) | Residents may not look for help mostly because of the stigma associated with seeking mental health care and an increased expectation of high performance and minimal complaints while under training. Mental health services, adequate mentoring, managing workloads, and social off-duty activities could mitigate the problem and reduce the long-term associated burden not only on future physicians’ quality of life and well-being but also on patients’ quality of care | The current study’s findings might be affected by the relatively small sample size (e.g., emergency residents group) which may lead to an overestimate of the depression prevalence and severity. Also, the study reflects the experience of a single residency training center in Saudi Arabia that limits generalizability of the findings. Other limitations are related to data self-reporting and the lack of gold-standard diagnostic clinical interviews. |
| Andejani et al., 2017 [24] | Saudi Arabia | . | Reducing the number of working hours, giving the post-call day off, and calling for a shift in institutional policies to support residents. Furthermore, treatment is indicated in residents with depressive symptoms. An American Council of Review Committee of residents and fellows conducted an inquiry on resident’s wellness and depression.12 The group recommended to increase the awareness of stress and depression during residency. They also recommended the promotion of a supportive culture and other measures to be taken by all stakeholders of postgraduate medical education.12 Screening residents in-training for depressive symptoms may not be practical. However, “facilitated discussion group intervention” has been found effective to reduce the prevalence of burnout and depression among high risk junior residents.13 Residents with moderate-to-severe depression require formal psychiatric assessment and anti-depression medications. An alarming finding was reported by Stoesser and Cobb14 who investigated depression among residents at the University of Utah: only 27% of residents with moderate to severe depression were receiving medication.  Co-authors suggest making 2 sets of examinations, 1 for junior and 1 for senior residents. Another suggestion that is being made is psychiatric assessment of the residents and treatment of depression if required. We also plan to repeat the study in a few years, after the implementation of these suggestions. | Small sample size Use of self-administered data |
| Ansaripour et al., 2016 [25] | Iran | Depression showed a higher rate in female health workers compared to males. There was a significant relationship between depression and age, work experience, and residential type (P < 0.050). Health workers’ performance had an inverse relationship with depression severity (P < 0.050). Moreover, the level of health workers’ education had an inverse relationship with depression and a direct relationship with performance. | . | . |
| Ardekani et al., 2008 [26] | Iran | Depression was more prevalent among single nurses(p<0.05) | Paying more attention to shift work nurses' health particularly female nurses, through shift work health education program and occupational health consultation and periodical examinations is recommended | . |
| Arefian et al., 2009 [27] | Iran | The two groups were statistically different for those aged more than 40 years (7.5% in the special and 19.5% in the general group, p value=0.04) but in those aged younger than 40, there was no difference. (p =0.05) | To recognize the differences and the prevalence of depression or anxiety in special and general wards nurses, more delicate prospective studies are needed and we recommend that the prevalence of depression in nurses regarding other contributing factors such as type of patient’s disease, nursing authority, and communication to doctors, patients and head-nurses be evaluated to devise better programs for these hard-working health care professionals. | . |
| Ariapooran, 2019 [28] | Iran | No statistically significant differences between female and male nurses suffering from depression Depression was found to be higher in single nurses than the married ones Workaholism is positively correlated to depression (p < 0.04) Work Compulsively is correlated to depression (p < 0.02) Age (p < 0.001) and job experience (p < 0.01) are positively correlated to depression and were statistically significant predictors of depression | Attention to sleep problems and depression among Iranian nurses and their predictive variables (such as workaholism) is very important for those who are dealing with resolving the mental problems of nurses. In addition, the use of psychological and therapeutic strategies to solve the psychological problems of nurses can be involved in reducing their psychological problems of nurses. | The limitations of this study were related to the statistical sample and using the self-reported questionnaires as tools for measuring the variables; that is to say, the sample was limited to the hospitals in Malayer. The results should be interpreted with caution. Moreover, answering self-report questionnaires is usually associated with bias. It is suggested that future studies use interviews to measure the rates of workaholism, sleep problems, and depression. |
| Asad Zandi et al., 2011 [29] | Iran | Scores of depression were higher in female nurses than male nurses. Significant relation between the age and depression but there was no correlation between the age and depression.  There was a significant association between the prevalence of depression and marital status (married), (higher) education level and (higher) hours of overtime per week. | Awareness of the symptoms will lead to future interventions and increased mental health and occupational satisfaction in this group. Given that care and health sector is one of the most important areas of sustainable development of the health in human societies, therefore enhancement in service rendering and sufficient support will lead to healthy nurses and thereby society health promotion. Periodical study regarding levels of anxiety, depression and stress among nurses spatially nurses working at military hospitals is an essential work and is in line with prohibitive efforts of health system. Conducting comparative studies of these indicators among nurses of different countries will determine the difference of welfare and occupational level of this group and provide occupational welfare and mental safety of this patient and laborious class of the society. | It is worth to mention that lack of similar studies inside and outside the country and the confidentiality of the statistical data of military individuals are regarded as the limitations of this study and because of mentioned issues, comparison between similar individuals was not possible. |
| Ashraf et al., 2019 [30] | Pakistan | Significant association of person related burnout with depression (χ2=28.35, p<.0001 and work. Further, 5% (n=8) participants demonstrating excessive severe level of depression also exhibited severe level of person related burnout, 2% (n=4) of participants with excessive level of depression reported severe level of client related burnout, and 4% of the sample illustrating excessive severe depression indicated severe level of work-related burnout. related burnout with depression (χ2=29.74, p<.0001). The findings indicated significant association of depression (χ2=31.28, p<.0001), anxiety (χ2= 23.03, p<.0001) and stress (χ2=38.81, p<.0001) with psychological burnout. Further, findings indicated that 6% (n=10) of the study participants reporting excessive severe depression also reported sever burnout. | . | This correlational study explained a link between psychological burnout and mental health issues but failed to draw a causal inference which limits the interpretations in the broader context. A longitudinal research with same parameters may provide causal inferences. Further, data was collected from urban areas of Islamabad/Rawalpindi which limits the generalizability of the findings to the exposure of stressful situations MHPs face in rural areas. Moreover, by controlling the confounding factors of subjects (e.g., age, gender, education, and work experience) and work related dimensions (e.g., placement, working hours, facilities), more rigorous effect of psychological burnout on mental health could be assessed. In extension to that dose-response relationship in context of work related stressors and mental and physical health complaints could be designed. |
| Aslam et al., 2013 [31] | Pakistan | No significant differences regarding gender and age | It is strongly suggested that measures should be adopted to reduce anxiety and depression in doctors working at resident level by reviewing and reforming training structure and introducing stress management training workshops | . |
| Atif et al., 2016 [32] | Pakistan | Doctors with lesser service had higher scores(p-0.011); 11.46% and 8.3% had less than 4 years and 5-9 years’ service respectively. | Prevention should be employed as best cure to eliminate anxiety and depression in doctors. Further research and analysis may yield more prolific results, helping us to formulate guidelines to screen anxiety and depression in doctors, and deal with suffers more aptly and at early stages. Healthy doctors can warranty explicit health care delivery. | Generalization of the study cannot be warranted as all doctors of Lahore could not be screened. Response rate was low, thus selection bias was inevitable. Respondents could be more health conscious, having extra interest in medical research, could spare time to participate or they may be less afraid of stigmatization of having mental health. disorder. There is a high probability of interview bias as at least some of respondents may not be willing to declare their actual mental health state. |
| Badahdah et al., 2020 [33] | Oman | Using a chi-square analysis revealed that a significantly higher proportion of females scored less than 28 compared to males (χ2 (1) = 3.14, p = .05). Similarly, a significantly higher proportion of HCWs who did not work with COVID-19 patients scored higher than 28 compared to those who did (χ2 (1) = 3.27, p = .05). The same test revealed no significant differences in the proportion of nurses and doctors (χ2 (1) = .68, p = .24), nor in the proportion of married and nonmarried who scored <28 on WHO-5 (χ2 (1) = 1.94, p = .11). | Support for HCWs, especially those who display signs of trauma and stress, is critical as we go through this cata- strophic global pandemic. Fear of contagion, stress, anxiety and concern for their well-being and significant others endanger the mental health of HCWs. There are many options for health care leaders to support and protect HCWs during this difficult time, such as implementing mindfulness and cognitive behavioral therapy intervention programs | First, we relied on a convenience sample, which is not representative of HCWs in Oman. Therefore, the findings are not generalizable and should be interpreted with some caution. Second, the cross-national design of this study prohibits us from drawing conclusions about causality. Thus, more research is needed, especially longitudinal studies, to allow us to identify the important causes of psychiatric problems among HCWs. Finally, because we recruited our participants via WhatsApp, we could not calculate a response rate. |
| Bana et al., 2019 [34] | Pakistan | Depression was found to be highest in second group of more than five years of professional experience; n=69 (71.8%) and the calculated P value= 0.010. | Different types of stressors should be incorporated in the questionnaire so that mental health promotion and mental disorders preventive strategies would be formulated by targeting the type of stresses. This is also recommended that stress management together with personal and professional awareness training should be included in the undergraduate curriculum so that threats to mental and social wellbeing, which might occur during the professional life, can be avoided or addressed. Further research and analysis may yield more prolific results, helping us to formulate guidelines to screen anxiety and depression in dentists, and deal with suffers more appropriately and at early stages. Healthy dentists can warranty explicit oral health care delivery. | Sample technique was also the limitations of this study and the results from our sample cannot be deduced to the wider population of dentists in Karachi, Pakistan. |
| Behnam et al., 2016 [35] | Iran | There was a significant inverse correlation between serum vitamin D levels and depression score (r = -0.338, P = 0.001). | . | . |
| Bukhari et al., 2016 [36] | Pakistan | . | This was a cross-sectional study and cannot establish causality link between occupational risk factors and depression. Furthermore, the study was conducted on a sample of nurses working in a federal government tertiary care hospital in Islamabad and as such may not be generalizable to those working in provincial government or private sector hospital settings. | Longitudinal studies should be conducted to confirm the present findings |
| Bukhari et al., 2019 [37] | Pakistan | Statistically significant association was found between depression and the nurses who had a work experience of 5–7 years (p=0.000) The prevalence of depression in nurses working on rotating shifts was significantly higher (p=0.012) as was the case with those having inflexible working hours (p=0.032). A statistically significant association was found between depression and nurses who thought that they had more responsibilities and less authorities at work (p=0.018), who believed that they had to work fast at job (p=0.004), who thought that they had to do extra physical work at job (p=0.003) and who had suffered from injury during job (p=0.010). A statistically significant higher proportion of nurses who had experienced verbal abuse by patient or attendant were suffering from depression (p=0.001) as well as those who had experienced harassment by patient or attendant (p=0.001). A statistically significant association was found between depression and nurses who were not satisfied from their job (p=0.012) and who believed that their job is stressful (p=0.000) Higher prevalence of depression in nurses who were not satisfied from their job (55.6%) compared to those who were satisfied (36%). | . | This was a cross-sectional study and cannot establish causality link between occupational risk factors and depression. Longitudinal studies should be conducted to confirm the present findings. Furthermore, the study was conducted on a sample of nurses working in a federal government tertiary care hospital in Islamabad and as such may not be generalizable to those working in provincial government or private sector hospital settings. |
| Dachraoui et al., 2017 [38] | Tunisia | . | . | . |
| Darawad, 2009 [39] | Jordan | . | . | As the present study was cross-sectional, it is difficult to conclude on causal directions. Furthermore, actigraphic assessment or administration of sleep diaries to objectively quantify sleep disorders would have been a point of strength. Linking the findings with work errors and quality of performance together with the screening of cancer susceptibility would have added much to the current findings. |
| Dehghan et al., 2012 [40] | Iran | Prevalence of depression in women and single rural health workers was more than male and married rural health workers (p<0.05) | Efforts should be made to remove depression symptoms in the group that will lead to better performance and will lead to community health promotion | . |
| Dehghani et al., 2009 [41] | Iran | Depression intensity of Nurses who work in emergency ward and critical care units were more than depression level of the rest(P=0.001). Also, there was significant statistical relationship between depression severity and Nurses' satisfaction of their sleep (P=0.015). | Hospital nurse offices should use the psychiatric mental health nurse for consult services and education to nurses about coping strategies and management of depressed mood | . |
| El Kissi, 2014 [42] | Tunisia | Prevalence of Major Depressive Episodes (MDE) was associated with females (p = 0.017) as well as distance from the workplace in km (p = 0.016).  The prevalence of MDE was associated with family history of organic pathologies and personal history of organic pathologies and psychiatric.  As for occupational factors, the prevalence of MDE was associated with relational working conditions, feeling perceived security and the desire to change establishment. | There is a need to use a validated international diagnostic tool for search for specific diagnostic entities.  The solutions adopted to deal with the stress among nurses would be ineffective if they are not integrated into a common strategy encompassing all factors involved.  Health authorities should carry out a regular recruitment of nurses allowing compensate for the growing shortage of staff, as well as a continuing education.  Hospital administrations should review the distribution of staff, timetables and the organization of tasks.  Occupational medicine would play a role fundamental role in screening for health problems in relationship with any suffering at work. | We only studied the nurses of the CHU Farhat Hached, which does not allow us to generalize our given to other circles such as regional hospitals or hospitals with greater admissions.  A service-based analysis, although important parameters were not conducted given the small sample size. |
| El-Hamrawy et al., 2018 [43] | Egypt | Socioeconomic standard was of no statistical significance in relation to depressive symptoms Factors affecting depressive symptoms among doctors with statistical significance are women sex (65.4%), married (63.8%), income not enough, and unpaid loans and not enough and big loans (100%), postgraduate degree (64.4%), assigned doctors (69%); factors affecting depressive symptoms for nurses with statistical significance are women sex 60.8%), mean age (42.72 years), not enough and small loan income (88.6%), college degree (65.3%), nurses (62.5%), have mean years of experience (22.22), mean working hours per week (41.52), and dealing with patients per day of a mean (36.57) | . | . |
| Farag et al., 2019 [45] | Egypt | Socioeconomic stress it was found to be significantly related to depression (P value 0.008) and to suicide (P-value 0.010), While suicide was evidently found to be related to depression (P-value <0.001). Beck depression inventory was found to be statistically significant (P value 0.025) with pediatrics department having the least percentage of residents scoring normal (0% out of 13 residents) followed by Obstetrics and gynecology (3 out of 19 residents, 15.79%) with equal ranges in internal medicine (30 out of 101 residents, 29.70%), surgery (10 out of 33 residents, 30.30%) and in anesthesia (3 out of 13 residents, 23.08%) while in departments not dealing with patients as radiology and clinical pathology only 7 out of 25 residents (28%) were found to have normal ups and downs. | Professional and personal stress is a significant and frequently overlooked component of a resident’s life. In addition, residents are at higher risk to develop depression as studies have suggested that medical students experience high rates of depression and suicidal ideation. Intervention studies are needed, e.g. support groups, more intense coaching by senior physicians, training programs on ‘breaking bad news’ and teaching of stress management skills to study how to prevent or reverse burnout. | . |
| Ghazwin et al., 2016 [47] | Iran | Depression scale was significantly related to scores of anxiety and stress (P<0.0001, r=0.75 and‎ P<0.0001, r = 0.76, respectively) female gender was correlated to more severe depression (P: 0.010) More severe depression ‎was related to lower scores of SWLS (P: 0.001, r = -0.32) Marital status was not related to severity of depression | . | Satisfaction with life is a complex construct, which is affected by various factors. In this study, ‎some of the demographic and personal variables had been taken into consideration. However, ‎many environmental factors including social support, which have the potential to affect life ‎satisfaction, were not assessed. It is recommended that future studies evaluate the social ‎determinants of satisfaction with life in Iranian nurses. ‎ |
| Halayem-Dhouib S et al., 2010 [49] | Tunisia | High scores in emotional tiredness (1 domain of burnout) were correlated to depression (P=0.000; R=0.56) | . | . |
| Halvani et al., 2012 [50] | Iran | There was also a significant association between depression and marital status (Married > single) (P-Value=0.009, F=6.93), morning shifts and turn over shifts (P-Value=0.032, F=1.11), the satisfaction level of occupation (P-Value=0.000, F=7.641), and satisfaction of the employer (P- Value=0.001, F=5.414). | . | . |
| Haqqi, 2013 [51] | Pakistan | No statistically significant relationship between depression and gender, training levels or campuses | . | . |
| Hasan et al., 2018 [52] | Egypt | A positive significant correlation was detected between depression and stress level (Pearson's correlation = 0.710 and p = .01), which reflects that when the level of work-related stress increased, the level of depression also increased. Depression and coping strategies (Pearson's correlation = -0.80 and p = .01) Marital status and working unit, years of experience were significant predictors of using effective coping strategies, accounting for 67% of the variance in the depression level [F (3, 32) = 8.722, P ˂0.05].  A total of 78.5% of variance in depression level [F (3,14) = 16.984] was explained by age, level of education, shift and working hours per week | Moreover, empowering psychiatric nurses may increase self-efficacy and reduce the psychological distress experienced by improving coping strategies, making problematic behavior more understandable and therefore less burdensome. Further studies need to measure empowerment and psychological levels. To implement programs aimed at teaching psychiatric nurses how to deal with occupational stress and its effects, as well as improving their coping strategies and problem- solving ability. | . |
| Hassannia et al., 2020 [53] | Iran | The prevalence of depression was significantly higher in doctors and nurses compared with other occupations (OR=1.5, 95% CI: 1.154-2.021, P=0.003). The prevalence of depression was not significant in education levels (general vs specialist physicians) (X2=22.800, P=0.064). | . | In this cross-sectional study, we were not able to establish a causal link; furthermore, we used the self-rating scale to evaluate the depression and anxiety symptoms of the medical staff and the general public. Finally, the age group did not include those above 60 years of age possibly due to the lack of access to Internet smartphones, or applications. |
| Ibrahim et al., 2016 [54] | Saudi Arabia | . | Screening programs for IBS, anxiety, depression, and sleep disorders are required. Multifaceted approaches are needed to decrease symptoms among patients, such as dietary education and management of psychosocial problems. Further multicenter studies are recommended to increase the representation of male nurses. | . |
| Ibrahim et al., 2019 [55] | Saudi Arabia | The odds of having symptoms of depression were 61% higher for nurses with 6–7 h of sleep per day (OR = 1.61; 95% CI: 1.17–2.22) and 110% higher for nurses with ≤5 h (OR = 2.10; 95% CI: 1.36–3.25) (fully adjusted model; reference ≥8 h) The odds of a ‘mild to moderate’ depression score were 54% higher for nurses with 6–7 h of sleep per day (OR = 1.54; 95% CI: 1.10–2.16) and 65% higher for nurses with ≤5 h (OR = 1.65; 95% CI: 1.04–2.64). The odds of a ‘severe to very severe’ depression score were 93% higher for nurses with 6–7 h of sleep (OR = 1.93; 95% CI: 1.10–3.39) and 321% higher for nurses with ≤5 h of sleep (OR = 4.21; 95% CI: 2.18–8.11) (fully adjusted model; reference: ≥8 h) The odds of a ‘severe to very severe’ depression score were 93% higher for nurses with 6–7 h of sleep (OR = 1.93; 95% CI: 1.10–3.39) and 321% higher for nurses with ≤5 h of sleep (OR = 4.21; 95% CI: 2.18–8.11) (fully adjusted model; reference: ≥8 h). Adjustment for age and other covariates made little change to the estimates. Trend tests indicated that there was a monotonic increase in the odds ratios of depression across the decreasing sleep categories, and these trends were statistically significant  The odds of severe to extremely severe depression were highly significant for the comparison categories (ie, sometimes, usually, and always) when compared with the reference (ie, never) for job insecurity and fear of litigation variables. The corresponding strength of associations for those comparison categories for a mild to moderate form of depression were much less strong, albeit significant | Hospital and nursing administrators should address long working hours and heavy workloads (e.g., nurse to patient ratio) in order to allow nurses sufficient time to sleep. Furthermore, administrators should initiate employee health programs that promote positive lifestyle choices. | Our estimates might not be totally accurate as sleep data was not assessed by a validated scale, and the DASS-21 is not a diagnostic tool for depression. Our analysis considered a single aspect of sleep, i.e., duration, and lacked vital information on other aspects such as sleep quality, sleep latency, sleep efficiency, sleep disturbance, medication use, and daytime dysfunction. We also lack information on chronic diseases and pain, which could have been critical in the analyses. |
| Jabeen et al., 2020 [56] | Pakistan | . | . | This study has limitations. First, data obtained from self-reported questionnaires was not verified with medical records. Secondly the study did not assess socioeconomic status of different HCWs groups. Finally, this study was done in one medical college and its attached teaching hospital. The results obtained cannot be generalized. For this purpose, a larger, multicenter study would be required. |
| Kamimura et al., 2018 [57] | Iraq | The following factors were associated with higher levels of depression: fewer work hours per week (p<0.05), sleep problems in the past 2 weeks (p<0.01) and lower job satisfaction (p<0.05). | Due to these safety concerns, it is important to develop policy and safety measures to improve physician work environments in Iraq in order to make remaining in Iraq more attractive to Iraqi medical school graduates Future research should identify factors causing sleep problems in order to develop intervention programs for physicians. | Since this study used an anonymous online survey, there was no way to check the eligibility of the participants. However, this study did not offer any incentives to participants. Thus, it is very unlikely that it had ineligible participants. Because the link to the survey was forwarded from initial contacts, a response rate is unknown. Additionally, since the total number of the participants was very small, the sample size may not large be enough to generalize the results to the entire breadth of the young physician population in Iraq. Like any cross-sectional research, this study did not identify causal directions among variables. |
| Kashani et al., 2017 [58] | Iran | . | . | One of the limitations of this study was its low sample size, which may influence the power of the study. The nature of evaluating personality dis- orders is another limitation of this study. In most cases, personality disorders are not a single problem and several diagnoses are made for an individual. Therefore, it is possible that the reported percentages are different from reality to some extent. In addition, the psychologist who makes the diagnosis also plays a role. |
| Kassani et al, 2014 [59] | Iran | According to path analysis, important related variables with depression were respectively, mental domain Quality of Life (QOL): 28%, married status: 25%, physical domain QOL: 24%, shift working: 18%, age: 16%(, and overtime hours working: 12%; that direct effect of these variables were more than indirect effect; through of mental and physical domains of nurses’ QOL on their depression . | Nurse managers must be planning to increase nurses’ mental health, attention to variety aspects of family such as marriage, decrease and distribution of times working especially in night shift could be increased quality of life in nurses and decreased their depression. | . |
| Kassani et al., 2015 [60] | Iran | Independent Variables having direct effects on nurses' depression:  age (p=0.01); Overtime (p=0.04); Night shift (p=0.03); Marital status (p=0.02); History of depression (p=0.01); Sex (p=0.02); Physical component of Quality of Life (p=0.01); Mental component of Quality of Life (p=0.01) | Promoting mental health is considered the index of social system efficiency which is, in practice, measured through the appraisal of people’s life in community. Policy makers and planners should pro- mote nurses’ quality of life with the available resources. Given the psychological and physical after-effects of night shifts as a contributing factor to depression, careful plans are necessary for reducing night shifts hours. Nurses’ physical health is also an important factor in de- pression as the better a nurse’ physical health, the lower the incidence of depression. In this regard, focusing on physical health through a healthy lifestyle could be helpful. Direct effects of quality of life (mental and physical components), marital status, night shifts, and overtime are important findings yielded from path analysis which makes it essential for policy makers and health care officials to closely concentrate on them. Further research can help scrutinize the relationship between depression and quality of life among nursing community. | . |
| Kavari et al., 2007 [61] | Iran | Connection between depression prevalence and marital status was meaningful (P<0.0001). Divorced and widow persons were more depressed than single and married persons. Meaningful relationship between intensity of depression and level of educations (P<0.005). Parent’s death before 11th age had a meaningful connection with depression (P<0.001) Over time hours in a week and intensity of depression had a meaningful connection with depression (P<0.02) | Head nurses especially should attend more to young nurses because age and work experience increases support. | . |
| Khalid et al., 2010 [63] | Pakistan | Nurses of 25-29 years group had the highest frequency of depression while nurses of 35-40 years age group had the highest severity of depression Nurses with 5-9 years length of service had the highest frequency (71.3%) of depression.  Nurses with 10-14 years length of service had the highest severity of depression (66.7%) (P value < 0.01) for all of the above | Recognizing problems and dealing with them positively and pro- actively, is the cost-effective way forward in the management of stress. These results imply that the focus of change should be on prevention at the primary level. For example, providing transport facility and better working environment, appropriate pay and benefits, revising job plans, regular vaccination program, ensuring adequate hours of work and adequate number of nurses to share the workload and responsibilities and provision of adequate resources and physical working conditions. In addition, the role of social support and relaxation techniques as stress coping strategies should not be overlooked as contributory factors for the well being of the nurses. Thus an integrated approach for successful occupational stress management should be advocated, which seeks to manage stress at the individual and organizational levels. Individual approaches include stress management training and one-to-one psychological ser- vice - clinical, occupational and health counseling while organizational interventions ranging from structural (for example work schedules, physical environment) to psychological (for example social support, control of over- work, participation). However, it is also necessary that nurses are encouraged to use these services. Studies have been conducted to evaluate the effects of coping strategies and job satisfaction to relieve the nursing work related stress. The key interventional strategies for managing work-related stress in relation to nursing are prevention, timely reaction and rehabilitation. | . |
| Khalilzadeh et al., 2005 [64] | Iran | Significant relationship among job stress, depression and anxiety but the relationship of job stress and type of employment isn't significant | . | . |
| Khamseh et al., 2011 [65] | Iran | Depression & marital status (p<0.05) Depression & gender (p<0.05) | . | . |
| Khan et al., 2020 [66] | Saudi Arabia | . | future studies must focus on investigating the cognitive functions of paramedics during duty and any effects on their performance and with regards to safety. Employing more paramedics in ambulance services and implementing fatigue risk management strategies [62] may help to improve the sleep and mental health outcomes by reducing the workload. | Response bias is a common issue with such a design but can be solved by conducting follow-up or prospective cohort studies. In addition, self-selection bias is another common issue that may overestimate the current findings. A comparison between rosters (rotating shift vs fixed/rural shifts) could not be done due to the fact that the majority of both samples reported working in rotating shift schedule. The difference in the response rate for the Saudi and Australian paramedics is likely due to the recruitment procedure. The survey was conducted online for the Australian paramedics, whereas the Saudi paramedics were actively recruited at workstations with printed copies. The usage of emails for work purposes is different between Saudi and Australia. So, the recruitment procedure was conducted in such a way as to make the study known to the Saudi paramedics. In addition, this study included only male paramedics from Saudi Arabia, as this occupation employs only males in Saudi Arabia, which may affect the comparison to previous findings. However, a comparison was conducted to only male paramedics from Australia. Moreover, due to data availability, the study could not specify the paramedic/citizen ratio for the studied states (Makkah District in Saudi and Victoria in Australia). Generalizing to the total population sizes was the closest available way of describing this relationship. |
| Khani et al., 2016 [67] | Iran | There was a significant relationship in depression with workplace and the number of shifts per month. (P = 0.0001). | Identifying the factors that influence the incidence of depression is very important and it is necessary for decisions making regarding prevention or remediation of such problems. | . |
| Khodadadi et al., 2016 [68] | Iran | Women had higher depression scores than men (p<0.05) Significant inverse relationship between education level and depression rate among nurses with a graduate or Masters of Science in Nursing (MScN) degree at (p<0.05) Having interest in the nursing profession had an inverse statistical significance depression among nurses (p<0.05) Statistically significance between marital status and depression, whereas married nurses had less depression than others (p<0.05) | We recommend an overview and revision in the Iranian healthcare system and modification of current situation for nurses by adopting new strategies to address the stated problems and resolve the issues. There is a need for nurse managers to develop appropriate intervention programs to reduce workload, make regular shift schedules, and provide positive reinforcements for nurses to reduce anxiety, stress and depression. This intervention strategy may help increase job satisfaction and demonstrate organizational commitment in nurses' health and wellbeing | Sampling population that could not be generalizable as a full representative of Iranian nurses. Second limitation related to the use of certain hospital wards such as medical, surgical, pediatric, ICU, CCU, emergency and burn unit, which omitted other specialties and could not be generalizable, similar to other studies recommending further studies to encompass all areas of nursing profession. Third limitation involved data collection based on nurses’ self-reports, which could have been biased. |
| Kousha et al., 2018 [69] | Iran | Depression was significantly higher in resident physician younger than 30 years. significant relation between higher scores of stress and depression in single residents. Depression was not significantly different in males and females, surgery or non-surgery field of work, year of residency, and residents who had a higher number of night staying in hospital Higher Emotional Intelligence appears to be good predictors of low depression in a resident physician β: -0.145 SD: 0.017 P<0.0001). | . | The most important limitation in this study was low response rate (50%). We did not use any reward for participants and the term of intelligence may suggest some sort of to be judge. Self‐rated method used for gathering data could led to information bias. Furthermore, distribution of residents in different level was not balanced; finally, the cross‐sectional method used in the present study, limited result for interpretation of observed associations between measured factors. We did not differentiate dimensions of Emotional Intelligence. |
| Lafta et al., 2016 [71] | Iraq | Doctors’ exposure to violence was significantly associated with depressive symptoms. A significant association was found between reporting depressive symptoms and exposure to insult, assault or threats ng many cases of violent injury/death during work (OR= 4.834, p .004), Exposure to pressure during work was also sign (OR=2.448, p < .001), exposure to any sort of violence at work (OR=2.547, p .002), or outside the work (OR=1.841, p .008). A significant association was also found between depression and history of killing, kidnapping or injury to family members (OR= 1.769, p .012) or colleagues (OR= 1.796, p .031) and seeing many cases of violent injury/death during work (OR= 4.834, p .004), Exposure to pressure during work was also significantly associated with depressive symptoms (OR=2.455, p .002). | . | As anxiety and depression could be multifactorial; the effect of confounders (personality, family history of psychological problems, threshold of stress) couldn’t be evaluated, however, we did not attempt to diagnose these diseases but we, rather, classified the respondents as having “probable” anxiety or depression on symptomatic basis. |
| Maghrabi et al., 2019 [72] | Saudi Arabia | Statistically significant association between workability index and DP (P=0.006) Lack of personal accomplishment (P= 0.019) and depression (P<0.001). No statistically significant difference in depression by setting | There is a need to establish system in order to early recognize physician at risk, and provide support system and programs to train them of coping strategies. Future research needed to focus on confirming the result and establish a causal relation between workability index and burnout syndrome and depression. | First, the study is cross sectional that has less determine of causal effect. Second, the survey was online and self-reported with no observation. Third, outside stressors was not assessed. |
| Mahmodi et al., 2013 [73] | Iran | Prevalence of major depression in night shift nurses was 5.6%, compared to day shift nurses, which was 3.4%, but not statistically significant Relationship between depression and job position was significant (P=0.01) No significant correlation between depression and demographic characteristics such as age, gender, marital status, job history and educational level | Paying more attention to shift work nurses' health, particularly female nurses, through shift work education program, occupational health consultation and periodical examinations is recommended. | . |
| Mahmoudi et al., 2009 [74] | Iran | Significant relationship was observed between degree of depression and nurse's gender, ie female (p=0.007) and occurrence of main (depressive) event in the last year (p=0.018).  No significant relationship was observed between degree of depression and age (p=0.112), marital status (p=0.393), number of children (p=0.768), type of service (p=0.605), years of experience (p=0.452), work place (p=0.592), income (p=0.793), birth order (p=0.206), accommodation (p=0.433) and educational degree (p=0.252) | Considering the high prevalence rate of depression in the nurses, screening test for diagnosis of depression and its beginning at time of treatment accompanied with follow up seems necessary | . |
| Malik et al., 2017 [75] | Bahrain | Family physicians were found to be 4.5 times more likely to be depressed than consultant family physicians (P = 0.001) Primary Care Providers (PCPs) with salaries ranging from 1200 to 1600 BD were four times more likely to be depressed than those earning more than 2500 BD (P = 0.032) PCPs who worked for < 40 h per week were 14.3 times more likely to be depressed than those who worked for > 50 h per week (P = 0.019). Whereas PCPs who spent 30-40 h (P = 0.016) consulting patients per week were 13.1 times more likely to be depressed compared to those who spent < 30 h per week. With regards to patient load, PCPs who consulted < 200 patients per week were 33.3 times and 27 times more likely to be depressed compared to those who consulted 200-350 patients (P = 0.001) and > 350 patients per week (P = 0.008), respectively PCPs who took more than 3 days of sick leaves (P = 0.011) in the past 6 months were 7.2 times more likely to suffer from depression than those who took 0-1 days leave during the same period. Finally, PCPs who answered “No” (P < 0.001) when asked about job satisfaction were 12 times more likely to be depressed compared to their satisfied colleagues. Despite being statistically insignificant, PCPs with factors such as taking > 30 min to reach workplace (P = 0.105) were found more likely to exhibit depression | Intervention is promptly required for PCPs, where full assessment, support, and therapy needs to be provided for current sufferers of DAS. Increasing allocated time per patient during consultation could be a solution to ensure adequate care was provided. Also, a higher salary bracket could act as an incentive for doctors, who are increasingly dissatisfied with their jobs. Improving public education and community awareness could also decrease the burden on health centers and their doctors | This study might have been biased, as some PCPs were made to answer under duress, specifically due to time constraints. Also, the study might have a limitation since the obtained data were self- reported, hence, some degree of response bias due to under-reporting might have occurred. Some of the PCPs did not do the same amount of work and did not consult the same number of patients adding to selection bias. There were other confounding factors, which were not kept in consideration, for example, most of the PCPs were women in the study and women being more prone to mental illness compared to men. |
| Mami et al., 2014 [76] | Iran | Male nurses showed to have a significantly higher level of depression (p<0.05) A significant difference was observed between the depression level and job satisfaction (p<0.05) Depression level of nurses was not significantly affected by depression level and marital variables (p>0.05) and satisfaction with accommodation facilities (p>0.05) | Considering the importance of nursing occupation as well as the findings of our research, it seems necessary to consider the problem of depression among working nurses more seriously since depression adversely affects on their function and performance qualities to patients. It should be particularly considered in male nurses more seriously due to the extra over-load and over-time work they should experience as a routine | . |
| Marzouk et al., 2018 [77] | Tunisia | In comparison with residents without definite depression (HAD-D<11), the group of residents with definite depression (HAD-D≥11) was older and more often married (45.7% vs 36.2%, p<0.0001). Surgical specialties with high workload (22.9% vs 17.5%, p=0.011) were significantly associated with depressive symptoms in contrast to medical specialties (44.7 vs 52.6%, p=0.003), which were associated with lower depressive symptom Poisson regression analysis disclosed the following variables as associated with the total HAD score: age (OR=1.014, 95% CI 1.006 to 1.023, p=0.001), female gender (OR=1.114, 95% CI 1.083 to 1.145, p<0.0001), number of night shifts per month (OR=1.048, 95% CI 1.016 to 1.082, p=0.03) and number of working hours per week (OR=1.008, 95% CI 1.005 to 1.011, p≤0.0001). Compared with medical specialties, the medico-surgical ones were independently associated with a higher HAD score (OR=1.459, 95% CI 1.172 to 1.816, p=0.001 | Any strategy should target the individual level to amend the identified and potentially actionable factors, and include more measures targeting the general organization of residents’ work modalities, and residents' relationship with hierarchy. Structures able to provide aid to health professionals exposed to and suffering from stress, anxiety and depression are anyway non-existent in Tunisian hospitals. Our study suggests that such structures can no longer be considered an option, but the Ministry of Health should provide support at the institutional level. At the individual level, more generally accepted risk factors such as older age, gender, marital status, stressors outside of work, sleep deprivation or lifestyle require more personal attention and lifestyle education. Residency program factors and the pace of work should also be better managed.  Although still debated, the issue of work-hour restriction could be effective in reducing high emotional exhaustion, despite the fact that it carries the risk of alteration in the quality of care and education by reducing the number and the actual presence of medical residents. | . |
| Mehdi et al., 2020 [78] | Pakistan | Females were slightly more depressed than males Moderate depression was reported equally by doctors and nurses. | There should be an unambiguous assurance to the health care workers about the safety of themselves and their families. | Due to the nature of the cross-sectional data, it is challenging to mark pivotal implications. There is still a need for further studies to validate the facts and also to determine more factors leading to anxiety and depression. |
| Mirmohammadi et al., 2009 [79] | Iran | This study showed the higher frequency of depression in shift workers comparing to non-shift-workers and the difference is statistically significant | . | . |
| Mobasher et al., 2015 [80] | Egypt | There is a positive correlation be- tween scores of Internet Addition Test (IAT) and BDI-II; this correlation is of statistical significance (P=0.000) A mild positive correlation between scores of BDI-II and hours spent online per week; this correlation is of statistical significance (P = 0.000) | There is a need for a larger funded analytical study with a random representative sampling method, for more accuracy to study the internet use, behavioral problems associated with Pervasive Internet Use (PIU) in Egypt, and to determine the temporal relationship between PIU and depressive disorders. | Findings of this study, as well as other studies reported in literature, indicate that there is an associative relationship between PIU and depressive disorders. However, as- sociation does not mean causality. |
| Mogharab et al., 2016 [81] | Iran | Depression was correlated negatively with intrinsic religious orientation (r, -0.37; P = 0.001) and positively with extrinsic religious orientation (r, 0.24; P = 0.01). Depression was significantly more prevalent among female nurses (P = 0.03). | Specialized counseling services by religious experts can help improve nurses’ religiosity and reduce their depression. | . |
| Mohammed et al., 2014 [82] | Egypt | Female residents were significantly different from their male colleagues regarding severity of depression (p< 0.001). Higher distribution of more severe depression among female residents Married and single residents differed significantly regarding the severity of depression(p=0.003) All (100%) of severely depressed participants were single while 60% of the married group showed minimal-to-mild depression Neither depression nor burnout was found to correlate with work hours. Both depression and burnout affected the physicians no matter what work schedule they had. A total of 100% of the moderately-to-severely depressed residents were in the burnout group. Minimally depressed residents were 14 times less likely to get burned-out (OR 0.07) while moderately depressed residents were 32 times more likely to have burnout A significantly positive correlation was found between emotional exhaustion and severity of depression (r=0.61; p< 0.001) A significantly positive correlation was found between depersonalization and severity of depression (r=0.63; p<0.001) A significantly negative correlation was found between personal accomplishment and severity of depression (r=-0.56; p< 0.001) | . | Residents' response rate (64.6%) seemed rather low.  A major limitation of the study was the small sample size. The anonymous nature of the questionnaires is a potential source of selection bias that is difficult to assess. For example, residents with higher levels of burnout may have been less inclined to take on the additional task of completing the questionnaires. Also, despite the promise of anonymity, residents may have been anxious about providing answers critical of the training programs. A limitation inherent in correlational research designs that utilize surveys for data collection is the respondents’ self-reported information on the survey41. Consequently, respondents may report what they think rather than what they do41. In turn, the participants’ responses may be a truthful representation of their thoughts and attitudes, but may not be an accurate representation of their actions and circumstances. The authors used the cut-off points of Maslach et al.16 and Beck et al.15, which need not be the same for Egyptians, as both tools have not been standardized on an Egyptian population. There is a limited amount of literature on the Egyptian physicians’ context on the relation between depression and burnout. There was no separate control group, but the use of non- burned-out and minimally-to-mildly depressed physicians as the comparison group meant that both groups experienced similar conditions, giving a high degree of matching. |
| Mohammed et al., 2019 [83] | Sudan | Statistically there was insignificant association between demographic characteristics (Gender, Age and Marital Status) and HADS Scale (Depression) (P-value > 0.05), and we found a highly significant association between depression and level of education (more protective for PhDs than Master's than Bachelor's) | The demographic risk factors that found to be associated depression included the academic levels. This factor can be rectified by provision of better work condition to abolish or reduce their negative impact as risk factors for depression. Further studies needed to elucidate the full dimension of this issue to propose effective tools to manage them for the sake of this important health sector and their clients. | . |
| Momeni et al., 2016 [84] | Iran | Participants with a history of previous mental health problem, were more exposed to depression than ones without (P=0.002) | . | The main limitations of our study were small sample size and short duration of follow-up. Furthermore, we did not include a control group to compare the prevalence of psychiatric disorders with the general population. Further controlled investigations are recommended with longer follow-up to validate findings reported here. |
| Monsef et al., 2015 [85] | United Arab Emirates | Depression was more common in residents with the income less than 10000 AED (69.1%) and the difference from other groups was statistically significant (p value: 0.046) | Further research should be conducted using other tools like DSM-IV or PHQ-9. Establishing residency counseling office is suggested to deal with residents’ problems in way that supports their needs and leads to a best working environment | . |
| Mustahsan et al., 2013 [86] | Pakistan | 30% of sleep deprived doctors were diagnosed with depression | . | . |
| Naser et al., 2020 [87] | Jordan | Being female [OR=1.48, 95% CI: 1.11 – 1.97], divorced [OR=1.86, 95% CI: 1.02 – 3.38] and a pulmonologist [OR=4.17, 95% CI:1.94 – 9.00] were RF for depression | These findings raise awareness amongst policy makers and mental health providers in order to take the necessary measures to attend to psychological wellbeing of individuals during global pandemic. Further studies to investigate the impact of time on mental health are needed. | There are limited studies that explored the prevalence of depression and anxiety during COVID-19 pandemic worldwide and in the Middle East specifically, a fact that limited our ability to compare our findings with similar healthcare environment and culture. The sample size of ENT physicians’ subgroup was small due to small population in this category nationwide. The impact of time on mental health was not captured here due to the nature of this study and further studies are necessary. It would be useful to repeat the study after the COVID-19 pandemic reach a peak to determine the effect of time on the results. Although depression and anxiety are closely related, depression is almost related to disparate life events, and needs a longer duration in time than the 2-weeks, that are monitored by the PHQ-9 instrument. However, this remark is not valid for anxiety and GAD-7 is relevant to the subject. The above-mentioned remarks may explain the surprising conclusion that USs are more affected and have higher depression and anxiety rates compared to HCPs who are in the center of the risk and seriously affected by this pandemic disease. Finally, we used an online survey for data collection and therefore, we may have missed some of the targeted population. |
| Nisar et al., 2012 [88] | Pakistan | . | Adequate salaries/fringe benefits should be offered to doctors. Adequate recreational facilities, leaves and suitable pays should be granted to doctors. The placement of doctors at various appointments should be in accordance with their capabilities/experience and qualifications. Security of doctors/healthcare organizations should be assured. WHO should play an important part in this regard at global level and the state governments should play their role at national level. | . |
| Nooli et al., 2017 [89] | Saudi Arabia | . | . | Limitation of the study is that the study was cross-sectional survey. |
| Pournamdarian et al., 2012 [90] | Iran | Meta cognitive beliefs, especially negative beliefs associated with the uncontrollable and the need to control thoughts, are the best predictor for depression, anxiety and stress. | . | . |
| Poursadeghiyan et al., 2016 [91] | Iran | Work related stress was significantly associated with depression (p-value: 0.006) | The findings suggest that there is need to pay attention to knowledge about the development of preventive strategies against stress as well as conducting rehabilitation exercises for nurses with psychological distress in the work place, thereby decreasing work-related stress and subsequently develop nurses’ mental health which should be used as part of the management strategies of organizations. Further researches that have the tendency to explore specific strategies for stress management may reduce the impact of stress on mental health of nurses and subsequently lessen absenteeism and turnover. The achievement of these evidence-based strategies that are intended to make better work environments where the security of nurses is guaranteed and they have enough resources to successfully complete their jobs will help improve their health outcomes. | . |
| Raessi et al., 2015 [92] | Iran | Depression, and Herzberg hygiene factor (p < 0/01 , r = - 0/ 227 )  Depression, and Herzberg motivational factor (p < 0/01, r = - 0/ 227) are significant. | . | . |
| Raffah et al., 2013 [93] | Saudi Arabia | Females likelier to be depressed (46.2% Vs 21.9%) p<0.03  Average number of patients seen per day for physicians without depression was 53±19 and among depressed physicians was 65 ± 34 patients. P value < 0.03. | We recommended that female physicians have less workload, more leave schedule according to her physiological changes and psychology and less working hours. This arrangement has to be carried out by high authority with ministry of health. As one of the risk factors is seeing more patients, therefore need more physicians to be assigned in Primary Health Care Centers to handle the workload | . |
| Rahmati et al., 2019 [94] | Iran | . | . | Small sample size, which was also seen in previous studies. Inability to control some confounding factors such as the menstrual cycle, and personal and family problems were also among the limitations of this study. Another important point is that a control group was not available for performing more comparisons. |
| Raza et al., 2017 [95] | Pakistan | Prevalence of depression significantly elevated in private sector hospitals (p=0.003) Positive smoking history and presence of chronic disease (p=0.01) | . | . |
| Roughani et al., 2019 [96] | Iran | The study showed that there is a significant relationship between job stress and stress associated with life with depression (p = 0.001, p = 0.004, respectively). There was no significant relationship between depression and educational degree (p=0.56), specialty of nurses (p=0.78), marital status (p = 0.16), gender (p=0.3), type of employment (p = 0.18). | Attention should be paid to stress and stressful occupation of nursing and to think about it and to take preventive measures. | . |
| Sadeghi et al., 2007 [97] | Iran | Symptoms of depression were 2.3 times more frequent in females | . | . |
| Shabany et al., 2018 [98] | Iran | The correlation coefficient between BDI and total EI was 0.2, P was 0.008, indicating weak statistical significance. | . | First, it was conducted at university affiliated wards and second, the authors did not include nurses of all wards. |
| Sheikh et al., 2018 [99] | Pakistan | Anxiety scores were strongly associated with depression scores (r=0.65, P <0.001) | . | . |
| Taghinejad et al., 2014 [100] | Iran | . | . | Considering the data collected by questionnaires, honesty of respondents was kept covert to researchers; hence, before distribution of questionnaires to nurses the aims and significance of the study were justified and explained to them. |
| Taghva et al., 2014 [101] | Iran | . | In addition to concern for mental fatigue and burnout among nurses in ICU and surgical wards, it is important to pay attention to psychological issues of nurses in other wards - including psychiatry - which have a prominent role in the promotion of mental health. | . |
| Tajvar et al., 2015 [102] | Iran | No significant differences between demographic features of the nurses and depression as subscale of mental disorders (p > 0.05) | Results can be used to establish policies for hospitals to promote the health and welfare of their staff members. A comprehensive health program be implemented in this field to reduce occupational stress and enhance the level of nurses’ mental health so that the effectiveness and performance of the ICU can be improved. As suggested by other authors, training programs to enhance communication skills could be beneficial in improving basic/intuitive communication strategies; it seems apparent that providing training programs will help promote safety and improve health in the workplace | A limitation of this study is that the number of nurses who participated in the study was relatively small. It is suggested that the association of these factors be investigated further in future studies. |
| Talih et al., 2016 [103] | Lebanon | Depression was found to be significantly correlated with mental health services use (r = 0.29, p = 0.001), self-administration of psychotropic medication (r = 0.45, p < 0.001), anxiety (r = 0.43, p < 0.001), burnout (r = 0.72, p < 0.001), and drug abuse(r = 0.25, p = 0.007). Anxiety (β = 0.37, p < 0.001) and family/ social instability (β = 0.37, p < 0.001) were significant predictors of depression scores, R2 = 30 %, p < 0.001 | Implications for Academic Leaders: Balancing residents’ training needs with a manageable workload remains a significant challenge. Residents’ psychological well-being is an integral part of their health and ability to function properly | The main limitations are that our results were obtained in a cross- sectional design, which precludes evaluation of temporality and causality of the observed relationships and the relatively small sample size. Therefore, these results should be interpreted as associations rather than as definitive causation. Larger samples are needed to better clarify the associations among these variables. Our study is limited by the low response rate and the small numbers of residents in some specialties. Low response rates could be associated with stigma. We did not have data on the non-responders and they may be more depressed, burned out, or have more suicidal ideation than responders. Another limitation is the exclusive reliance on self-reported scales rather than diagnostic interviews. tools used in our study, although well validated, cannot diagnose mental health disorders, so results should be interpreted cautiously. |
| Talih et al., 2018 [104] | Lebanon | Depression was found to be significantly correlated with anxiety (r = .50, p < .001), burnout (r = .48, p < .001), and drug abuse (r = .30, p = .001) Anxiety (ß = .30, p < .001) and burnout (ß = .27, p < .001) were significant predictors of PHQ-9 scores Personal or social stressors were not correlated with depressive symptomatology in this sample. | It is worth noting that the overall study results suggest that both residents and nurses at the same facility are struggling to cope with depression and burnout. Culturally, in Lebanon a physician’s role is more valued socially and this is a possible ameliorating factor for residents. Promoting more empathy and collaboration between residents and nurses may be of mutual benefit to both groups. Nursing and healthcare administrators should routinely educate and raise awareness among nurses regarding depression and burnout and provide better working conditions such as better pay, better scheduling and stress free environments. Providing mental health interventions for those at risk is an important investment for healthcare systems in developing countries. | However, the institution surveyed is a training center and recruits nurses from across the country. Lebanon is a geographically small and culturally diverse country making this sample reasonably representative. Findings of this study should be interpreted with caution and within the context of Lebanon’s cultural diversity and postwar situation. Although the civil war officially ended around 1991, its political, social and financial sequelae have an ongoing negative impact on the Lebanese population The low response rate and a relatively small sample size is another limitation which lowers the sample’s representativeness and as such, these results should be interpreted as associations rather than definitive causation. Low response rates could be due to stigma and fear of discrimination in the workplace. Culturally in Lebanon there is considerable stigma regarding mental health issues. This stigma is likely to have adversely affected the overall response rate. Also we suggest that responders may be more psychologically minded or in actual distress when compared to non-responders, which raises the question of self-selection or response bias. It is important to note that the residents surveyed at the same medical center as part of the same research project had double the response rate. One potential explanation is that residents may be less busy than nurses and may have more time to participate in the sur- vey. Residents may be less concerned with stigma due to their exposure to psychiatry in medical school clerkships. Finally, it is possible that due to burnout among the non-responders, they were not motivated enough to respond to the questionnaire, which may have been viewed as burdensome by exhausted nurses. |
| Vahedian-Azimi et al., 2020 [105] | Iran | The average score of depression in female medical staff was higher than males (F/M: 27.08±4.6 vs. 25.33±4.3, P=0.011, 95% CI: 0.39-3.08) Marital status had not affected the level of stress, anxiety and depression of patients in any group (P>0.05) | Designing psychological interventions is essential for improving mental health during and after the pandemic. | . |
| Yasemi et al., 2014 [106] | Iran | Prevalence of depression was higher among women, singles, smokers, physically inactive individuals, younger age, and anesthesia personnel, although the difference was statistically insignificant (P>0.05) | Reducing nurses’ working hours and holding educational and counseling courses for improving their lifestyle are recommended | . |
| Younesi et al., 2017 [107] | Iran | Age, gender, marital status, shift work were not significantly correlated with depression scores | Hence, planning to meet the needs of personnel and training classes in various fields is very necessary. | . |
| Yousuf et al., 2011 [108] | Pakistan | The significant (p < 0.25) independent predictors of depression among post graduate trainees were none or occasional peer support (OR 2.05; CI=1.01, 4.18) and working hours less than 76 hours (OR 3.716; CI=1.677, 8.233) and 76-90 hours (OR3.150; CI= 1.422, 6.976) compared to those working >91 hrs / week Age(>30), religion (being Muslim), ethnicity(Urdu speaking), marital status(being married), living status(with family), specialty (faculty of Internal Medicine followed by Allied Surgery, Allied Medicine and General Surgery), supervisor support(No or occasionally), not enough time for academics, and smoking status(not smoking) were also associated with depression | There is also an urgent need to develop programs for screening of medical and surgical post graduate trainees for detecting depression earlier and take appropriate actions to manage this morbid condition. | Convenience sampling was used to draw the sample; which is inferior to probability sampling in representation of the population, and this limits the external validity of the study. However, efforts were made to include respondents from different departments of the hospital to provide better overall representation. As the study was carried out in only one institute it cannot be generalized. Some potential confounding variables were not evaluated. For example, it is possible that any recent stressful event like death in the immediate family or preparing for exams in the near future or presence of any co morbid condition could possibly result in depression. The survey was done in the summer and did not ask about seasonal depressive effects. Therefore surveying trainees at a different time of the year could have resulted in different rates of depression. Although we used the validated tool for the assessment of depression, recommended by WHO for study purposes, it is not yet validated in Pakistani population. The reason for not using a validated tool was because no tools for measuring depression only have been validated in Pakistan. Validating the tools was not in the scope of this study. This could have led to imprecise measure of some outcome variables and leaves room for more comprehensive studies. |
| Zaher et al., 2016 [109] | Iran | Significant difference between nurses working in intensive care units and internal and surgical in terms of average depression (p=0.006) No significant correlation can be seen that would be associated with incidence of depression and age, type of employment, education, marital status and working experience (p> 0.005). But there is a significant correlation between gender and incidence of depression among the nurses (p<0.005) and the incidence of mood disorders in nurses and the rate of depression female nurses is more than male nurses (p <0.005) | . | . |

**Additional Material 6: Excluded Studies**

| **Excluded Study** | **Reason of Exclusion** |
| --- | --- |
| Abo Ali EA et al., 2015 [111] | No tool used to measure depression |
| Almehdar AS et al., 2019 [112] | Not outcome of interest |
| Ariapooran S et al., 2019 [113] | Not outcome of interest |
| Atawneh FA et al., 2003 [114] | No tool used to measure depression |
| Basfr W et al., 2019 [115] | No tool used to measure depression |
| Bazazan A et al., 2018 [116] | Not outcome of interest |
| Ben-Ezra M et al., 2011 [117] | Non-EMR population |
| Ben-Ezra et al., 2013 [118] | Non-EMR population |
| Brahem A et al., 2016 [119] | Not population of interest |
| Bushra R et al., 2010 [120] | Not population of interest |
| Choobineh A et al., 2006 [121] | Not outcome of interest |
| Elsayed S et al., 2018 [122] | Overlapping data with another study |
| Erol A et al., 2007 [123] | Non-EMR population |
| Hussain SS et al., 2014 [124] | No tool used to measure depression |
| Jafei W et al., 2003 [125] | No tool used to measure depression |
| Jahrami H et al., 2012 [126] | Not outcome of interest |
| Kasemy ZA et al., 2016 [127] | Not population of interest |
| Khan SA et al., 2012 [128] | Not outcome of interest |
| Kheyri F et al., 2017 [129] | Not outcome of interest |
| Khuwaja AK et al., 2004 [130] | Not outcome of interest |
| Mushtaq M et al. 2015 [131] | Not outcome of interest |
| Nabi N et al., 2012 [132] | Not outcome of interest |
| Saifan AR et al., 2019 [133] | Not outcome of interest |
| Saquib J et al., 2020 [134] | Overlapping data with another study |
| Saquib N et al., 2019 [135] | Overlapping data with another study |
| Tomas-Sabado J et al., 2010 [136] | Non-EMR population |
| Zafar W et al., 2016 [137] | Not outcome of interest |
| Zaghloul MS et al., 2019 [138] | Overlapping data with another study |
| Zahid MA et al., 1999 [139] | No tool used to measure depression |

**Additional Material 7: Quality assessment of primary studies**

| **Included study citation** | **1. Were study participants sampled in an appropriate way?** | **2. Was the sample size adequate?** | **3. Were the study subjects described in detail?** | **4. Was the setting described in detail?** | **5. Were valid methods used for the identification of the condition?** |
| --- | --- | --- | --- | --- | --- |
| Abbas et al., 2012 [3] | Unclear | Low risk of bias | Low risk of bias | Low risk of bias | Low risk of bias |
| Abdulrahman et al., 2018 [4] | Unclear | Low risk of bias | Low risk of bias | Low risk of bias | Low risk of bias |
| Ahmed et al., 2009 [5] | Unclear | High risk of bias | High risk of bias | Low risk of bias | Low risk of bias |
| Al Ghailani et al., 2018 [6] | Unclear | Low risk of bias | High risk of bias | Low risk of bias | High risk of bias |
| Al-Amer et al. 2020 [7] | Low risk of bias | Low risk of bias | Low risk of bias | Low risk of bias | Low risk of bias |
| Al-Ghafri et al., 2014 [8] | Low risk of bias | Low risk of bias | Low risk of bias | Low risk of bias | Low risk of bias |
| Al-Hammad et al., 2012 [9] | Unclear | Low risk of bias | Low risk of bias | Low risk of bias | Low risk of bias |
| Al-Houqani et al., 2020 [10] | Low risk of bias | Low risk of bias | Low risk of bias | Low risk of bias | Low risk of bias |
| Al-Hussein et al., 2010 [11] | High risk of bias | Low risk of bias | Low risk of bias | Low risk of bias | Low risk of bias |
| Al-Maddah et al., 2015 [12] | Unclear | Low risk of bias | Low risk of bias | Low risk of bias | Low risk of bias |
| Al-Zahrani et al., 2014 [13] | High risk of bias | Low risk of bias | Low risk of bias | Low risk of bias | Low risk of bias |
| Albajjar et al., 2019 [14] | Low risk of bias | High risk of bias | Low risk of bias | Low risk of bias | Low risk of bias |
| AlFahhad, 2018 [15] | Unclear | Low risk of bias | Low risk of bias | Low risk of bias | Low risk of bias |
| AlFaris et al., 2019 [16] | High risk of bias | Low risk of bias | Low risk of bias | Low risk of bias | Low risk of bias |
| AlGhamdi et al., 2020 [17] | High risk of bias | Low risk of bias | Low risk of bias | Low risk of bias | Low risk of bias |
| AlHarby, 2009 [18] | Unclear | Low risk of bias | Unclear | Low risk of bias | Low risk of bias |
| Alhifzi et al., 2018 [19] | Low risk of bias | High risk of bias | Low risk of bias | Low risk of bias | Low risk of bias |
| Alipoor et al., 2015 [20] | Low risk of bias | Low risk of bias | High risk of bias | Low risk of bias | Low risk of bias |
| Alkhazrajy et al., 2014 [21] | Unclear | Low risk of bias | Low risk of bias | Low risk of bias | Low risk of bias |
| Almutairi et al., 2020 [22] | Unclear | Low risk of bias | Low risk of bias | Low risk of bias | Low risk of bias |
| Alshardi et al., 2019 [23] | Unclear | Low risk of bias | Low risk of bias | Low risk of bias | Low risk of bias |
| Andejani et al., 2017 [24] | Low risk of bias | High risk of bias | Low risk of bias | Low risk of bias | Low risk of bias |
| Ansaripour et al., 2016 [25] | Low risk of bias | Low risk of bias | Unclear | Low risk of bias | Low risk of bias |
| Ardekani et al., 2008 [26] | High risk of bias | Low risk of bias | Low risk of bias | Low risk of bias | Low risk of bias |
| Arefian et al., 2009 [27] | Low risk of bias | Low risk of bias | High risk of bias | Low risk of bias | Low risk of bias |
| Ariapooran, 2019 [28] | Low risk of bias | Low risk of bias | Low risk of bias | Low risk of bias | Low risk of bias |
| Asad Zandi et al., 2011 [29] | Low risk of bias | Low risk of bias | Low risk of bias | Low risk of bias | Low risk of bias |
| Ashraf et al., 2019 [30] | Low risk of bias | Low risk of bias | High risk of bias | Low risk of bias | Low risk of bias |
| Aslam et al., 2013 [31] | Low risk of bias | Low risk of bias | Low risk of bias | Low risk of bias | Low risk of bias |
| Atif et al., 2016 [32] | High risk of bias | High risk of bias | Low risk of bias | Low risk of bias | Low risk of bias |
| Badahdah et al., 2020 [33] | Low risk of bias | Low risk of bias | Low risk of bias | Low risk of bias | Low risk of bias |
| Bana et al., 2019 [34] | High risk of bias | Low risk of bias | Low risk of bias | Low risk of bias | Low risk of bias |
| Behnam et al., 2016 [35] | Unclear | Low risk of bias | Low risk of bias | Low risk of bias | Low risk of bias |
| Bukhari et al., 2016 [36] | Low risk of bias | High risk of bias | Low risk of bias | Low risk of bias | Low risk of bias |
| Bukhari et al., 2019 [37] | Low risk of bias | High risk of bias | Low risk of bias | Low risk of bias | Low risk of bias |
| Dachraoui et al., 2017 [38] | Unclear | Low risk of bias | Low risk of bias | Low risk of bias | Low risk of bias |
| Darawad, 2009 [39] | High risk of bias | Low risk of bias | Low risk of bias | Low risk of bias | Low risk of bias |
| Dehghan et al., 2012 [40] | Unclear | High risk of bias | Unclear | Low risk of bias | Low risk of bias |
| Dehghani et al., 2009 [41] | Unclear | Low risk of bias | High risk of bias | Low risk of bias | Low risk of bias |
| El Kissi et al., 2014 [42] | Low risk of bias | Low risk of bias | Low risk of bias | Low risk of bias | Low risk of bias |
| El-Hamrawy et al., 2018 [43] | Low risk of bias | Low risk of bias | High risk of bias | Low risk of bias | Low risk of bias |
| Fahim et al., 2018 [44] | Unclear | High risk of bias | Unclear | Low risk of bias | Low risk of bias |
| Farag et al., 2019 [45] | Unclear | Low risk of bias | Low risk of bias | Low risk of bias | Low risk of bias |
| Farahani et al., 2017 [46] | Unclear | Low risk of bias | Unclear | Low risk of bias | Low risk of bias |
| Ghazwin et al., 2016 [47] | Unclear | High risk of bias | High risk of bias | Low risk of bias | Low risk of bias |
| Habibi et al., 2014 [48] | Unclear | High risk of bias | Low risk of bias | Low risk of bias | Low risk of bias |
| Halayem-Dhouib S et al., 2010 [49] | Unclear | High risk of bias | Unclear | Low risk of bias | Low risk of bias |
| Halvani et al., 2012 [50] | Unclear | Low risk of bias | Low risk of bias | Low risk of bias | Low risk of bias |
| Haqqi, 2013 [51] | Unclear | High risk of bias | Unclear | Low risk of bias | Low risk of bias |
| Hasan et al., 2018 [52] | Unclear | High risk of bias | Low risk of bias | Low risk of bias | Low risk of bias |
| Hassannia et al., 2020 [53] | Low risk of bias | Low risk of bias | Unclear | High risk of bias | Low risk of bias |
| Ibrahim et al., 2016 [54] | Low risk of bias | Low risk of bias | Low risk of bias | Low risk of bias | Low risk of bias |
| Ibrahim et al., 2019 [55] | Low risk of bias | Low risk of bias | Low risk of bias | Low risk of bias | Low risk of bias |
| Jabeen et al., 2020 [56] | High risk of bias | Low risk of bias | Low risk of bias | Low risk of bias | Low risk of bias |
| Kamimura et al., 2018 [57] | High risk of bias | Low risk of bias | Low risk of bias | High risk of bias | Low risk of bias |
| Kashani et al., 2017 [58] | Low risk of bias | Low risk of bias | Low risk of bias | Low risk of bias | Low risk of bias |
| Kassani et al, 2014 [59] | Unclear | Unclear | Unclear | Low risk of bias | Low risk of bias |
| Kassani et al., 2015 [60] | High risk of bias | Low risk of bias | Low risk of bias | Low risk of bias | Low risk of bias |
| Kavari et al., 2007 [61] | High risk of bias | Low risk of bias | High risk of bias | Low risk of bias | Low risk of bias |
| Kazemi et al., 2010 [62] | High risk of bias | Low risk of bias | Unclear | Low risk of bias | Low risk of bias |
| Khalid et al., 2010 [63] | High risk of bias | High risk of bias | Low risk of bias | Low risk of bias | Low risk of bias |
| Khalilzadeh et al., 2005 [64] | Low risk of bias | Low risk of bias | Unclear | Low risk of bias | Low risk of bias |
| Khamseh et al., 2011 [65] | Low risk of bias | Low risk of bias | Unclear | Low risk of bias | Low risk of bias |
| Khan et al., 2020 [66] | Unclear | Low risk of bias | Low risk of bias | Low risk of bias | Low risk of bias |
| Khani et al., 2016 [67] | Low risk of bias | Low risk of bias | Unclear | Low risk of bias | Low risk of bias |
| Khodadadi et al., 2016 [68] | Low risk of bias | Low risk of bias | Low risk of bias | Low risk of bias | Low risk of bias |
| Kousha et al., 2018 [69] | Unclear | Low risk of bias | Low risk of bias | Low risk of bias | Low risk of bias |
| Koushali et al., 2013 [70] | Low risk of bias | Low risk of bias | Low risk of bias | Low risk of bias | Low risk of bias |
| Lafta et al., 2016 [71] | Low risk of bias | Low risk of bias | Low risk of bias | Low risk of bias | Low risk of bias |
| Maghrabi et al., 2019 [72] | Low risk of bias | Low risk of bias | Low risk of bias | Low risk of bias | Low risk of bias |
| Mahmodi et al., 2013 [73] | Low risk of bias | Low risk of bias | Unclear | Low risk of bias | Low risk of bias |
| Mahmoudi et al., 2009 [74] | Low risk of bias | Low risk of bias | Low risk of bias | Low risk of bias | Low risk of bias |
| Malik et al., 2017 [75] | Low risk of bias | Low risk of bias | Low risk of bias | Low risk of bias | Low risk of bias |
| Mami et al., 2014 [76] | Unclear | Unclear | Unclear | Low risk of bias | Low risk of bias |
| Marzouk et al., 2018 [77] | Low risk of bias | Low risk of bias | Low risk of bias | Low risk of bias | Low risk of bias |
| Mehdi et al., 2020 [78] | Unclear | Low risk of bias | Low risk of bias | Low risk of bias | Low risk of bias |
| Mirmohammadi et al., 2009 [79] | Unclear | Low risk of bias | Unclear | Low risk of bias | Low risk of bias |
| Mobasher et al., 2015 [80] | Low risk of bias | Low risk of bias | High risk of bias | Low risk of bias | Low risk of bias |
| Mogharab et al., 2016 [81] | Low risk of bias | Low risk of bias | High risk of bias | Low risk of bias | Low risk of bias |
| Mohammed et al., 2014 [82] | Low risk of bias | High risk of bias | High risk of bias | Low risk of bias | Low risk of bias |
| Mohammed et al., 2019 [83] | Low risk of bias | Low risk of bias | Low risk of bias | Low risk of bias | Low risk of bias |
| Momeni et al., 2016 [84] | Low risk of bias | Low risk of bias | Low risk of bias | Low risk of bias | Low risk of bias |
| Monsef et al., 2015 [85] | Unclear | Low risk of bias | Low risk of bias | Low risk of bias | Low risk of bias |
| Mustahsan et al., 2013 [86] | Unclear | Low risk of bias | High risk of bias | Low risk of bias | Low risk of bias |
| Naser et al., 2020 [87] | High risk of bias | Low risk of bias | Low risk of bias | High risk of bias | Low risk of bias |
| Nisar et al., 2012 [88] | Low risk of bias | Low risk of bias | High risk of bias | Low risk of bias | Low risk of bias |
| Nooli et al., 2017 [89] | Unclear | High risk of bias | High risk of bias | Low risk of bias | Low risk of bias |
| Pournamdarian et al., 2012 [90] | Low risk of bias | Low risk of bias | High risk of bias | Low risk of bias | Low risk of bias |
| Poursadeghiyan et al., 2016 [91] | Unclear | Low risk of bias | Unclear | Low risk of bias | Low risk of bias |
| Raessi et al., 2015 [92] | Unclear | Unclear | Unclear | Low risk of bias | Low risk of bias |
| Raffah et al., 2013 [93] | Low risk of bias | High risk of bias | Low risk of bias | Low risk of bias | Low risk of bias |
| Rahmati et al., 2019 [94] | Low risk of bias | High risk of bias | Low risk of bias | Low risk of bias | Low risk of bias |
| Raza et al., 2017 [95] | Low risk of bias | Low risk of bias | Low risk of bias | Low risk of bias | Low risk of bias |
| Roughani et al., 2019 [96] | Low risk of bias | Low risk of bias | Low risk of bias | Low risk of bias | Low risk of bias |
| Sadeghi et al., 2007 [97] | Unclear | Low risk of bias | Unclear | Low risk of bias | Low risk of bias |
| Shabany et al., 2018 [98] | Low risk of bias | Low risk of bias | Low risk of bias | Low risk of bias | Low risk of bias |
| Sheikh et al., 2018 [99] | High risk of bias | Low risk of bias | Low risk of bias | Low risk of bias | Low risk of bias |
| Taghinejad et al., 2014 [100] | High risk of bias | High risk of bias | Low risk of bias | Low risk of bias | Low risk of bias |
| Taghva et al., 2014 [101] | Low risk of bias | Low risk of bias | Unclear | Low risk of bias | Low risk of bias |
| Tajvar et al., 2015 [102] | Low risk of bias | High risk of bias | Low risk of bias | Low risk of bias | Low risk of bias |
| Talih et al., 2016 [103] | Unclear | Low risk of bias | Low risk of bias | Low risk of bias | Low risk of bias |
| Talih et al., 2018 [104] | Low risk of bias | High risk of bias | Low risk of bias | Low risk of bias | Low risk of bias |
| Vahedian-Azimi et al., 2020 [105] | Unclear | Low risk of bias | Low risk of bias | Low risk of bias | Low risk of bias |
| Yasemi et al., 2014 [106] | Low risk of bias | High risk of bias | Low risk of bias | Low risk of bias | Low risk of bias |
| Younesi et al., 2017 [107] | Low risk of bias | High risk of bias | Low risk of bias | Low risk of bias | Low risk of bias |
| Yousuf et al., 2011 [108] | High risk of bias | Low risk of bias | Low risk of bias | Low risk of bias | Low risk of bias |
| Zaher et al., 2016 [109] | Low risk of bias | Low risk of bias | Low risk of bias | Low risk of bias | Low risk of bias |
| Zaki et al., 2016 [110] | High risk of bias | Low risk of bias | High risk of bias | Low risk of bias | Low risk of bias |

**Unclear:** not reported

**Sampling method**: Probability sampling= Low risk of bias; Non probability sampling= High risk of bias

**Sample size:** if >100 there is low risk of bias; if <100 there is high risk of bias

**Study subject description:** we considered the following: 1. specific population 2. sex 3. age. If all 3 parameters reported= low risk of bias; if only 1 or 2 parameters reported= high risk of bias

**Setting:** if reported, low risk of bias

**Valid screening / diagnostic tools employed for the measurement of outcome:** low risk of bias

**Additional Material 8: Depression prevalence by instrument**

A total of 17 different instruments were used to measure depression and dysthymia in the studies included in our review, only 4 of which were diagnostic instruments [Diagnostic and Statistical Manual of Mental Disorders-IV (DSM-IV); The World Health Organization Composite International Diagnostic Interview (CIDI); Minnesota Multiphasic Personality Inventory-2 (MMPI-2); and the Present State Examination/Schedules for Clinical Assessment in Neuropsychiatry (PSE-10/SCAN)]. Six primary studies [21, 42, 58, 80, 86, 94] utilized either these diagnostic instruments or trained mental health professionals to identify those with depression. One of these studies, which was conducted in Pakistan [86], described only risk and protective factors, but did not provide prevalence data. The remaining five studies were conducted in Egypt, Iraq, Iran, and Tunisia. El Kissi *et al* [42] was the only study to use the DSM-IV criteria (gold standard during the study time period) provided a prevalence of major depressive episodes (7.5%) as well as dysthymia (5.7%). The remaining studies utilized a variety of screening instruments with varying sensitivity and specificity.

The most frequently used screening instruments included the Beck Depression Inventory (BDI, n = 25), the Depression, Anxiety and Stress Scale – 21 (DASS-21) (n = 18) and the Hospital Anxiety and Depression Scale (HADS) (n = 14, Table 2). Interestingly, only eight studies utilized the updated Beck Depression Inventory-2 (BDI-2), published in 1996, compared to twenty-five studies that utilized the original Beck Depression Inventory (BDI), originally published in 1961. Three primary studies [34, 36, 37], all conducted in Pakistan, used the Aga Khan University Anxiety Depression Scale (AKUADS), which provides a combined prevalence of anxiety and depression. These studies were thus excluded from the meta-analysis. Some studies that utilized the same instrument used a variety of cutoffs to demarcate the differing levels of depression, and some studies used their own terminologies or added further categories to describe the levels of depression. Additionally, pooled prevalence computed for all professions and EMR countries was significantly different between the screening instruments, varying between 9.23% for DASS-42 to 45.9% for BDI-2 (p<0.0001).

The administration of diagnostic instruments is onerous, requires a lot of training, is time consuming, and sacrifices anonymity that would otherwise not be a problem with self-reported screening instruments. Moreover, the use of non-validated cutoff points even while utilizing the same instrument augments the heterogeneity. For instance, the official cut-of points for none, mild, moderate, and severe depression for the Beck Depression Inventory-2 (BDI-2) are: 0-13, 14-19, 20-28, and 29-63, respectively. While most studies utilized these cutoffs, several used 0-9, 10-19, 20-29 and 30-63, which are the cutoffs for the original version of the BDI. The wide variability in terms of instruments used and differing cutoffs, makes the comparison between different primary studies precarious. However, we attempted to do so by stratifying our results by the instrument wherever possible. As few studies utilized diagnostic instruments, these were grouped together for the meta-analysis. This illustrates a clear need for consistent use of a standard measurement for depression with corroborated cutoff thresholds.

**References**

1. Moher D, Liberati A, Tetzlaff J, Altman DG. Preferred reporting items for systematic reviews and meta-analyses: the PRISMA statement. J Clin Epidemiol. 2009;62(10):1006-12.

2. Beller EM, Glasziou PP, Altman DG, Hopewell S, Bastian H, Chalmers I, et al. PRISMA for Abstracts: Reporting Systematic Reviews in Journal and Conference Abstracts. PLOS Medicine. 2013;10(4):e1001419.

3. Abbas MAF, Abu Zaid L, Hussaein M, Bakheet KH, AlHamdan NA. Anxiety and depression among nursing staff at king fahad medical city, Kingdom of Saudi Arabia. J Am Sci. 2012;8(10):778-94.

4. Abdulrahman M, Nair SC, Farooq MM, Al Kharmiri A, Al Marzooqi F, Carrick FR. Burnout and depression among medical residents in the United Arab Emirates: A Multicenter study. J Family Med Prim Care. 2018;7(2):435-41.

5. Ahmed I, Banu H, Al-Fageer R, Al-Suwaidi R. Cognitive emotions: depression and anxiety in medical students and staff. J Crit Care. 2009;24(3):e1-7.

6. Al Ghailani B, Al Nuaimi MA, Al Mazrouei A, Al Shehhi E, Al Fahim M, Darwish E. Well-being of residents in training programs of Abu Dhabi health services. Ibnosina Journal of Medicine and Biomedical Sciences. 2018;10(3):77-82.

7. Al-Amer R, Malak MZ, Aburumman G, Darwish MM, Nassar MS, Darwish M, et al. Prevalence and Correlates of Psychological Reactions Among Jordanian Nurses During the Coronavirus Disease 2019 Pandemic. [Pre-print]. 2020.

8. Al-Ghafri G, Al-Sinawi H, Al-Muniri A, Dorvlo AS, Al-Farsi YM, Armstrong K, et al. Prevalence of depressive symptoms as elicited by Patient Health Questionnaire (PHQ-9) among medical trainees in Oman. Asian J Psychiatr. 2014;8:59-62.

9. Al-Hammad FA, Raheel H, Al-Baiz LE. The effect of shift work on psychological stress, sleep pattern and health of nurses working at a tertiary hospital, Riyadh. Middle East Journal of Nursing. 2012;6(6):14-20.

10. Al-Houqani F, Al-Mukhaini A, Al-Kindi R. Prevalence of Depression among Oman Medical Specialty Board (OMSB) Residents. Oman Med J. 2020;35(2):e116.

11. Al-Hussein RY, Al-Mteiwty AM. Point Prevalence of Depression, Anxiety and Stress Among Nurses And Para-Medical Staff in Teaching Hospitals in Mosul. Al-Taqani. 2010;23(5):116-27.

12. Al-Maddah EM, Al-Dabal BK, Khalil MS. Prevalence of Sleep Deprivation and Relation with Depressive Symptoms among Medical Residents in King Fahd University Hospital, Saudi Arabia. Sultan Qaboos Univ Med J. 2015;15(1):e78-84.

13. Al-Zahrani AH, Kalo BB. Depression among emergency room physicians and its associated factors in Makkah Al-Mokarramah Hospitals. International Journal of Medical Science and Public Health. 2014;3(12):1501-7.

14. Albajjar MA, Bakarman MA. Prevalence and correlates of depression among male medical students and interns in Albaha University, Saudi Arabia. J Family Med Prim Care. 2019;8(6):1889-94.

15. AlFahhad N. Prevalence and factors associated with depression among health care workers in National Guard Hospital in Riyadh, KSA. Int J Med Develop Countries. 2018;2(3):92-6.

16. AlFaris E, AlMughthim M, Irfan F, Al Maflehi N, Ponnamperuma G, AlFaris HE, et al. The relationship between study skills and depressive symptoms among medical residents. BMC Med Educ. 2019;19(1):435.

17. AlGhamdi BS, AlAtawi Y, AlShehri FS, Tayeb HO, Taleb HAA, Binsalman A. Psychological Distress during COVID-19 Curfews and Social Distancing in Saudi Arabia: A Cross-Sectional Study. Research Square [Pre-print]. 2020.

18. AlHarby NMR. Prevalence of Depression and its Associated Factors among Physicians in the Primary Health Care Centers, Jeddah, Saudi Arabia. 1425H: Saudi Board of Family Medicine; 2009.

19. Alhifzi S, Al-Ghonimy A, Al Aboudi M, Al Abdullah R, Olaish A, BaHammam AS. Assessment of Sleep Quality, Daytime Sleepiness, and Depression among Emergency Physicians Working in Shifts. Journal of Nature and Science of Medicine. 2018;1(1):17-21.

20. Alipoor R, Ebrahimi A, Omidi R, Hedayati A, Ranjbar H, Hosseinpour S. Depression, anxiety, stress and related demographic variables in nurses of Valiasr hospital in Fasa University of Medical Sciences in 2014. Pajouhan Scientific Journal. 2015;13(4):51-9.

21. Alkhazrajy LA, Sabah S, Hassan SM. Prevalence of depressive symptoms among primary health care providers in Baghdad. Int J Health Psychol Res. 2014;2:1-20.

22. Almutairi I, Al-Rashdi M, Almutairi A. Prevalence and predictors of depression, anxiety and stress symptoms in paramedics at Saudi Red Crescent Authority. Saudi Journal of Medicine and Medical Sciences. 2020;8(2):105.

23. Alshardi A, Farahat F. Prevalence and Predictors of Depression Among Medical Residents in Western Saudi Arabia. J Clin Psychol Med Settings. 2019.

24. Andejani DF, Al-Issa SI, Al-Qattan MM. Depressive Symptoms among Plastic Surgery Residents. Plast Reconstr Surg Glob Open. 2017;5(10):e1516.

25. Ansaripour S, Hasanzadeh A, Gramian N, Akhavan S, Moghadas T. The prevalence of depression and its impact on health workers' performance in Isfahan University of Medical Sciences, Iran, 2013. Journal of Isfahan Medical School. 2016;33(357):1884-90.

26. Ardekani ZZ, Kakooei H, Ayattollahi SM, Choobineh A, Seraji GN. Prevalence of mental disorders among shift work hospital nurses in Shiraz, Iran. Pak J Biol Sci. 2008;11(12):1605-9.

27. Arefian NM, Sedighi A, Sedighi AS, Noubahar MR. Depression in the Nurses of the Special Wards Versus Nurses of the General Wards - a Comparative Study. International Journal of Cancer Management (Iranian Journal of Cancer Prevention). 2009;2(3):143-50.

28. Ariapooran S. Sleep Problems and Depression in Iranian Nurses: The Predictive Role of Workaholism. Iran J Nurs Midwifery Res. 2019;24(1):30-7.

29. Asad Zandi M., Sayari R., Ebadi A., Sanainasab H. Abundance of depression, anxiety and stress in militant Nurses. Journal of Military Medicine. 2011;13(2):103-8.

30. Ashraf F, Ahmad H, Shakeel M, Aftab S, Masood A. Mental health problems and psychological burnout in Medical Health Practitioners: A study of associations and triadic comorbidity. Pak J Med Sci. 2019;35(6):1558-64.

31. Aslam A, Nauman A, Batool S. Frequency of anxiety and depression among doctors at postgraduate resident level. Pakistan Journal of Medical and Health Sciences. 2013;7(3):664-7.

32. Atif K, Khan HU, Ullah MZ, Shah FS, Latif A. Prevalence of anxiety and depression among doctors; the unscreened and undiagnosed clientele in Lahore, Pakistan. Pakistan Journal of Medical Sciences. 2016;32(2):294-8.

33. Badahdah A, Khamis F, Al Mahyijari N, Al Balushi M, Al Hatmi H, Al Salmi I, et al. The mental health of health care workers in Oman during the COVID-19 pandemic. International Journal of Social Psychiatry. 2020:0020764020939596.

34. Bana KFMA, Abbassi ZA, Saleem S. Assessment Of Depression Among The Dentists Of Karachi By Using AKUAD Scale. Journal of Bahria University Medical and Dental College. 2019;9(3):183-7.

35. Behnam B, Semnani V, Haddadnia F, Mirmohammadkhani M. Vitamin D serum levels in nurses in Semnan educational hospitals and its association with depression. Koomesh. 2016;17(2):313-22.

36. Bukhari GMJ, Shaista H, Abbasi MMJ. Frequency of depression in nurses working in various departments of a federal government hospital of Islamabad. Pakistan Journal of Public Health. 2016;6(1):41-4.

37. Bukhari GMJ, Habibullah S, Mushtaq M, Abbasi J, Malik JS. Occupational Factors Associated With Depression In Nurses Working In A Federal Government Tertiary Care Hospital In Islamabad. J Ayub Med Coll Abbottabad. 2019;31(3):233-6.

38. Dachraoui F, Mghirbi A, Adhieb A, Nakkaa S, Hraiech K, Ousji A, et al. Anxiety and depression in critial care staff of the university hospital of Monastir, Tunisia. Annals of Intensive Care. 2017;7(1):136.

39. Darawad MW. An examination of the role discrepancy, depressive symptoms, and turnover intention among the Jordanian nursing workforce: ProQuest Information & Learning; 2009.

40. Dehghan A, Ghavami L, Ghahramani F, Bazrafshan MR, Namavar S. Prevalence of Depression and Its Relation with Their Performance in Larestan Rural Health Workers in 2010. Journal of Rafsanjan University of Medical Sciences. 2012;11(1):79-84.

41. Dehghani M, Zoladl M, Boland-Parvaz S, Keshtkaran Z, Mahmoudi R, Jabbarnejad A. A survey on depression and its related factors in Nurses who work in Namazi Hospital of Shiraz University of Medical Sciences-2008. Iran Occupational Health. 2009;6(3):24-31.

42. El Kissi Y, Maarouf Bouraoui M, Amamou B, Bannour AS, Ben Romdhane A, Ben Nasr S, et al. [Prevalence of anxiety and depressive disorders among the nurses of Sousse Farhat Hached hospital: assessment by the Tunisian version of CIDI]. Tunis Med. 2014;92(1):18-23.

43. El-Hamrawy L, Hegazy N, El-Halawany S. Prevalence of depressive symptoms among healthcare providers in Shibin El-Kom city in Menoufia governorate. Menoufia Medical Journal. 2018;31(2):708-15.

44. Fahim A, Waheed A, Aly H. 762 Mental health morbidity among medical and surgical oncology residents. Occupational and Environmental Medicine. 2018;75(Suppl 2):A594.

45. Farag M, Elbatrawy A, Mahmoud D, Mohamed M. Depression and suicidal ideations in relation to occupational stress in a sample of Egyptian medical residents: Ain Shams University; 2019.

46. Farahani M, Khodadadi B, Dalvand S. The Study of the Prevalence of Depression and Associated Factors among Nurses in Khoram Abad Hospitals during 2016. Journal of Nursing and Midwifery Sciences. 2017;10.

47. Ghazwin M, Kavian M, Ahmadloo M, Jarchi A, Golchin Javadi S, Latifi S, et al. The Association between Life Satisfaction and the Extent of Depression, Anxiety and Stress among Iranian Nurses: A Multicenter Survey. Iran J Psychiatry. 2016;11(2):120-7.

48. Habibi R. The Relationship between Job Stress and Depression among Nurses in Shahid Rajaie Hospitals and social security of Qazvin city in 2012. Edrak. 2014;9(35):45-50.

49. Halayem-Dhouib S, Zaghdoudi L, Zremdini R, Maalej I, Bechir MB, Labbene R. [Burnout among mental health professionals: A Tunisian experience]. Rev Epidemiol Sante Publique. 2010;58(6):403-8.

50. Halvani GH, Salmani Nodoushan I, Hoboubati H, Salmani Nodoushan M, Jafari Nodoushan R, Hajian N. Effect of Shift Work on the Frequency of Depression in Nursing Staff of Yazd University of Medical Sciences. SSU. 2012;1(2):104-9.

51. Haqqi S, editor The prevalence of depression among resident doctors working in a teaching hospital in Karachi, Pakistan. International Journal Of Psychiatry In Clinical Practice; 2013: IInforma Healthcare Telephone House, 69-77 Paul Street, London EC2A 4LQ, England.

52. Hasan AA, Elsayed S, Tumah H. Occupational stress, coping strategies, and psychological-related outcomes of nurses working in psychiatric hospitals. Perspect Psychiatr Care. 2018;54(4):514-22.

53. Hassannia L, Taghizadeh F, Moosazadeh M, Zarghami M, Taghizadeh H, Fathi Dooki A, et al. Anxiety and Depression in Health Workers and General Population During COVID-19 Epidemic in IRAN: A Web-Based Cross-Sectional Study. MedRxiv [Pre-print]. 2020:2020.05.05.20089292.

54. Ibrahim NK, Al-Bloushy RI, Sait SH, Al-Azhary HW, Bar NH, Mirdad GA. Irritable bowel syndrome among nurses working in King Abdulaziz University Hospital, Jeddah, Saudi Arabia. Libyan J Med. 2016;11(1):30866.

55. Ibrahim AY, Chamsi Basha A, Saquib J, Zaghloul MS, Al-Mazrou A, Saquib N. Sleep duration is associated with depressive symptoms among expatriate nurses. J Affect Disord. 2019;257:658-61.

56. Jabeen Z, Rani A, Ahmed S, Ghaffar S, Kazmi TH, Wasim ud D. Psychological Impact of COVID-19 Pandemic on Health Care Workers: A Cross Sectional Study. Biomedica. 2020;36:209-13.

57. Kamimura A, Chen J, Nourian MM, Stoddard M, Sarray A. Stress, Depression and Job Satisfaction among Physicians in Iraq. Diversity & Equality in Health and Care. 2018;15(2):71-6.

58. Kashani P, Mirbaha S, Forouzanfar MM, Meschi F, Baratloo A. The Prevalence of Personality Disorders among Emergency Nurses Based on MMPI-2 Questionnaire; a Cross-sectional Study. Emerg (Tehran). 2017;5(1):e17.

59. Kassani A, Niazi M, Menati R, Alimohamadi Y, Menati W. Relationship between nurses’ depression and quality of life: applying path analysis model. Quarterly Journal of Nursing Management. 2014;3(2):61-9.

60. Kassani A, Menati W, Menati R, Khammarnia M. Path Analysis of Depression and Quality Of Life Among Nurses. Health Scope. 2015;4(4):e23353.

61. Kavari H, Helyani M, Dehghani V. A study of depression prevalence in nurses and it’s effective factors in Shiraz Namazi Hospital. Rawal Med J. 2007;32(2):184-6.

62. Kazemi M, K. K. A study of depression prevalence of nurses and it’s effective factors in Tehran. Nurse and Physician Within War. 2010;14:8-11.

63. Khalid S, Irfan U, Sheikh S, Faisal M. Frequency of stress and depression in female nurses working in a teaching hospital. Kust Med J. 2010;2(1):10-4.

64. Khalilzadeh R, Khalkhali H, Yavarian R. The relationship of job stress, depression and anxiety of nursing staff of Urmia University of Medical Sciences. J Urmia Nurs Midwifery Fac. 2005;3(1):10-7.

65. Khamseh F, Roohi H, Ebaady A, Hajiamini Z, Salimi H, Radfar S. Survey Relationship between demographic factors and stress, anxiety and‎ depression in nurses working in selected hospitals in Tehran city‎. Journal of Holistic Nursing And Midwifery. 2011;21(1):13-21.

66. Khan WAA, Conduit R, Kennedy GA, Abdullah Alslamah A, Ahmad Alsuwayeh M, Jackson ML. Sleep and Mental Health among Paramedics from Australia and Saudi Arabia: A Comparison Study. Clocks & Sleep. 2020;2(2):246-57.

67. Khani H, Ghodsi H, Nezhadnik H, Teymouri S, Ghodsi A. Depression and its relationship with hypochondriasis in nurses in Neyshabur, Iran. MILITARY CARING SCIENCES. 2016;3(1 (7)):-.

68. Khodadadi E, Hosseinzadeh M, Azimzadeh R, Fooladi M. The relation of depression, anxiety and stress with personal characteristics of nurses in hospitals of Tabriz, Iran. International journal of medical research & health sciences. 2016;5(5):140-8.

69. Kousha M, Bagheri HA, Heydarzadeh A. Emotional intelligence and anxiety, stress, and depression in Iranian resident physicians. J Family Med Prim Care. 2018;7(2):420-4.

70. Koushali AN, Hajiamini Z, Ebadi A, Bayat N, Khamseh F. Effect of Ramadan fasting on emotional reactions in nurses. Iran J Nurs Midwifery Res. 2013;18(3):232-6.

71. Lafta RK, Dhiaa S, Tawfeeq WA, Al-Shawi AF. Association of violence with anxiety and depression among Iraqi Junior doctors. International Journal of Applied Psychology. 2016;6(6):163-70.

72. Maghrabi M, Kafi H, Jan A, Al-Raddadi R. The prevalence of burnout syndrome and depression in relation to workability among

physicians, Jeddah, Saudi Arabia. Journal of Preventive Medicine and Holistic Health. 2019;5(2):99-105.

73. Mahmodi S, Zehni K. The Comparison of Depression Prevalence Between Shift Work Nurses in Education Hospitals of Kurdistan Medical Sciences University. Iranian Journal of Nursing Research. 2013;8(28):-.

74. Mahmoudi G, Vahedi M, Hasani S. Study of depression in nurses at the universities of medical sciences affiliated hospitals in 2007. World Applied Sciences Journal. 2009;6(9):1200-4.

75. Malik H. Prevalence of depression, anxiety, and stress among primary care physicians in the Kingdom of Bahrain. Journal of the Bahrain Medical Society. 2017;29(3):19-27.

76. Mami S, Mehdian K, Davoodian Z. Investigation of depression rate in nurses working at state hospitals of the city of and its associated factors. 2014;22:51-6.

77. Marzouk M, Ouanes-Besbes L, Ouanes I, Hammouda Z, Dachraoui F, Abroug F. Prevalence of anxiety and depressive symptoms among medical residents in Tunisia: a cross-sectional survey. BMJ Open. 2018;8(7):e020655.

78. Mehdi M, Waseem M, Rehm MH, Aziz N, Anjum S, Javid MA. Depression and Anxiety in Health Care Workers during COVID-19. Biomedica. 2020;36(Special Issue S2):233-8.

79. Mirmohammadi S, Mehrparvar A, FallahMehrjerdi A. The effect of shift work on depression in nurses. Occupational medicine quarterly journal. 2009;1(1):24-7.

80. Mobasher MW, Fouad AA, Enaba DA, Shawky K, Moselhy HF. Impact of depression on pathologic internet use among intern doctors of Cairo university hospital (Kasr Al-Ainy). Addictive Disorders and their Treatment. 2015;14(4):182-7.

81. Mogharab M, Nateghi K, Shamaie-Zavareh A, Sharifzadeh G. The Correlation of Religious Orientation with Depression Among Critical Care and Emergency Room Nurses. Modern Care Journal. 2016;13(3):e12470.

82. Mohammed KA-M, Ali EG, Youssef IM, Fahmy MT, Haggag WE-l. Depression and burnout among residents. Arab Journal of Psychiatry. 2014;25(1):40-51.

83. Mohammed IE, Abdelrahman A, Bauomey EE. Demographic Risk Factors of Depression among healthcare Providers in Alhasaheesa Locality, Sudan 2016. IOSR Journal of Nursing and Health Science. 2019;8(1):62-7.

84. Momeni M, Fahim F, Vahidi E, Nejati A, Saeedi M. Evaluation of factors affecting psychological morbidity in emergency medicine practitioners. World J Emerg Med. 2016;7(3):203-7.

85. Monsef N, Al Hajaj K, Al Basti A, Al Marzouqi E, Al Faisal W, Hussein H, et al. Perceived Depression, Anxiety and Stress Among Dubai Health Authority Residents, Dubai, UAE. Am J Psychol Cognitive Sci. 2015;1(3):75-82.

86. Mustahsan SM, Ali SM, Khalid F, Ali AA, Ahmed H, Hashmi SA, et al. Sleep deprivation and its consequences on house officers and postgraduate trainees. J Pak Med Assoc. 2013;63(4):540-3.

87. Naser AY, Dahmash EZ, Al-Rousan R, Alwafi H, Alrawashdeh HM, Ghoul I, et al. Mental health status of the general population, healthcare professionals, and university students during 2019 coronavirus disease outbreak in Jordan: A cross-sectional study. MedRxiv [Pre-print]. 2020:e01730.

88. Nisar K, Khan KH, Shah M. Anxiety and depression in doctors undergoing postgraduate training courses at Armed Forces Postgraduate Medical Institute Rawalpindi. J Ayub Med Coll Abbottabad. 2012;24(3-4):171-3.

89. Nooli AEA, Asiri AAA, Asiri ANA, Alqarni MAH, Alhilali FMS, Alayafi MAM, et al. Prevalence of Depression Among Medical Interns in King Khalid University. International Journal of Medical Research Professionals. 2017;3(6):131-3.

90. Pournamdarian S, Birashk B, Farid AA. The clarification of contribution of metacognitive beliefs in explaining the symptoms of depression, anxiety and stress in nurses. Knowledge & Research in Applied Psychology. 2012;13(49):86-94.

91. Poursadeghiyan M, Abbasi M, Mehri A, Hami M, Raei M, Ebrahimi MH. Relationship between job stress and anxiety, depression and job satisfaction in nurses in Iran. The social sciences. 2016;11(9):2349-55.

92. Raeissi P, Raeissi N, Shokouhandeh L. The Relationship between Nurses' Mental health and Working Motivation. Advances in Nursing & Midwifery. 2015;24(86):35-42.

93. Raffah EA, Alamir AM. Depression among primary health care physicians in Makkah Al-Mukarramah. American Journal of Research Communication. 2013(12).

94. Rahmati F, Safari S, Hashemi B, Baratloo A, Khosravi Rad R. Prevalence of Depression and Personality Disorders in the Beginning and End of Emergency Medicine Residency Program; a Prospective Cross Sectional Study. Arch Acad Emerg Med. 2019;7(1):e5.

95. Raza A, Zainab H. Depression Among Doctors of Selected Public and Private Tertiary Care Hospitals in District Peshawar. Journal of Rehman Medical Institute. 2017;3(1+2):1-8.

96. Roughani A, Shaidaei MFB, Rohani A, Delpishe A, Sharifi Z, Ahmadzadeh A. The Relationship between Hospital Occupational Stress and Prevalence of Depression in Nurses Working in Ilam Hospitals. Journal of Pharmaceutical Research International. 2019:1-8.

97. Sadeghi AE, Navidi M, Sadeghi M. Depression among resident doctors in Tehran, Iran. Iranian Journal of Psychiatry. 2007;2(1):50-2.

98. Shabany M, Ghajarzadeh M. Emotional intelligence and depression among hospital nurses of tehran university of medical sciences. Archives of Neuroscience. 2018;5(4):1-5.

99. Sheikh MH, Waqas A, Naveed S, Shoaib M, Yousuf S, Butt SR, et al. Association of cognitive impairment with sleeping difficulties, anxiety and depression among Pakistani physicians. J Pak Med Assoc. 2018;68(6):932-5.

100. Taghinejad H, Suhrabi Z, Kikhavani S, Jaafarpour M, Azadi A. Occupational Mental Health: A Study of Work-Related Mental Health among Clinical Nurses. J Clin Diagn Res. 2014;8(9):WC01-3.

101. Taghva A, Yazdani A, Ebrahimi M, Alizadeh K, Sakhabakhsh M. Prevalence of depression in psychiatric nurses and comparison with other parts of the AJA hospitals. Journal of Nurse and Physician Within War. 2014(23-24):11-6.

102. Tajvar A, Saraji GN, Ghanbarnejad A, Omidi L, Hosseini SS, Abadi AS. Occupational stress and mental health among nurses in a medical intensive care unit of a general hospital in Bandar Abbas in 2013. Electron Physician. 2015;7(3):1108-13.

103. Talih F, Warakian R, Ajaltouni J, Shehab AAS, Tamim H. Correlates of depression and burnout among residents in a Lebanese academic medical center: A cross-sectional study. Academic Psychiatry. 2016;40(1):38-45.

104. Talih F, Ajaltouni J, Farhood L. Depression and burnout among nurses in a Lebanese academic medical center. Journal Medical Libanais. 2018;66(2):92-7.

105. Vahedian-Azimi A, Moayed MS, Rahimibashar F, Shojaei S, Ashtari S, Pourhoseingholi MA. Compare the severity of psychological distress among four groups of Iranian society in COVID-19 pandemic. Research Square [Pre-print]. 2020.

106. Yasemi M, Peyman H, Khajavikhan J, Nasiri AA, Najafi F, Hemati K, et al. Prevalence of depression among nurses working in the operating rooms and intensive care units. Journal of zabol university of medical sciences and health services. 2015;6(4):70-7.

107. Younesi A, Pele A, Rahmani R. Checking public health of a hospital in the Gilan Province using a questionnaire. Annals of Tropical Medicine and Public Health. 2017;10(4):963-5.

108. Yousuf A, Ishaque S, Qidwai W. Depression and its associated risk factors in medical and surgical post graduate trainees at a teaching hospital: a cross sectional survey from a developing country. Journal of Pakistan Medical Association. 2011;61(10):968.

109. Zaher SJ, Vafaei M, Abianeh EE. Comparing Depression, Anxiety and Stress among theNurses in the Critical care and Internal Surgical units at the Selected Hospitals of the Social Security Organization of Tehran in 2016. International Journal of Medical Research & Health Sciences. 2016;5(9):254-61.

110. Zaki NFW, Denewar KAF, El Sherif MAF, Elweheid A, Ibrahim HF, Pandii-Perumal SR. Psychological correlates of shift work sleep disorder among a sample of Egyptian nurses. Arab Journal of Psychiatry. 2016;27(2):127-43.

111. Abo Ali EA, Saied SM, Elsabagh HM, Zayed HA. Sexual harassment against nursing staff in Tanta University Hospitals, Egypt. J Egypt Public Health Assoc. 2015;90(3):94-100.

112. Almehdar AS. Direct and indirect effects of dispositional gratitude on physicians' professional quality of life and family satisfaction: ProQuest Information & Learning; 2019.

113. Ariapooran S, Raziani S. Sexual satisfaction, marital intimacy, and depression in married Iranian nurses with and without symptoms of secondary traumatic stress. Psychological Reports. 2019;122(3):809-25.

114. Atawneh FA, Zahid MA, Al-Sahlawi KS, Shahid AA, Al-Farrah MH. Violence against nurses in hospitals: prevalence and effects. Br J Nurs. 2003;12(2):102-7.

115. Basfr W, Hamdan A, Al-Habib S. Workplace Violence Against Nurses in Psychiatric Hospital Settings: Perspectives from Saudi Arabia. Sultan Qaboos Univ Med J. 2019;19(1):e19-e25.

116. Bazazan A, Dianat I, Rastgoo L, Mombeini Z. Factors associated with mental health status of hospital nurses. International Journal of Industrial Ergonomics. 2018;66:194-9.

117. Ben-Ezra M, Palgi Y, Wolf JJ, Shrira A. Psychiatric symptoms and psychosocial functioning among hospital personnel during the Gaza War: a repeated cross-sectional study. Psychiatry Res. 2011;189(3):392-5.

118. Ben-Ezra M, Palgi Y, Shrira A, Hamama-Raz Y. Somatization and psychiatric symptoms among hospital nurses exposed to war stressors. Israel Journal of Psychiatry and Related Sciences. 2013;50(3):182-7.

119. Brahem A, Ilahi S, Maoua M, Kalboussi H, El Maalel O, Chatti S, et al. Depressive syndrome and absenteeism among health personnel in Sousse (Tunisia). Annales Medico-Psychologiques. 2016;174(7):557-63.

120. Bushra R, Aslam N. Prevalence of Depression in Karachi, Pakistan. Oman Med J. 2010;25(4):e020.

121. Choobineh A, Rajaeefard A, Neghab M. Problems related to shiftwork for health care workers at Shiraz University of Medical Sciences. East Mediterr Health J. 2006;12(3-4):340-6.

122. Elsayed S, Hasan AA, Musleh M. Work stress, coping strategies and levels of depression among nurses working in mental health hospital in Port-Said city. International Journal of Culture and Mental Health. 2018;11(2):157-70.

123. Erol A, Saricicek A, Gulseren S. Burnout in residents: Association with job satisfaction and depression. Anadolu Psikiyatri Dergisi. 2007;8(4):241-7.

124. Hussain SS, Rahim R. Bullying of Postgraduate Medical Trainees in Tertiary Care Hospitals. Journal of Postgraduate Medical Institute. 2014;28(3):251-6.

125. Jafei W, Yakoob J, Jafri N, Maloni M, Hamid S, Shah HA, et al. Irritable bowel syndrome in health care professionals in Pakistan. J Pak Med Assoc. 2003;53(9):405-7.

126. Jahrami H, Al-Shuwaikh Z, Panchasharam G, Saif Z. Prevalence of mental health disorders among health care providers in the Psychiatric Hospital, Bahrain. Arab Journal of Psychiatry. 2012;23(1):30-4.

127. Kasemy ZA, Salama AA, Abo Salem ME, Negm N. Factors related to depression symptoms among working women in Menoufia, Egypt. J Egypt Public Health Assoc. 2016;91(4):163-8.

128. Khan SA, Farooq S, Bano A. Anxiety and depression in nurses working in government tertiary care teaching hospitals of Peshawar Khyber Pakhtunkhwa and their relationship with job stress. JPMI - Journal of Postgraduate Medical Institute. 2012;26(1):34-8.

129. Kheyri F, Seyedfatemi N, Oskouei F, Mardani-Hamooleh M. Nurses' mental health in Iran: A national survey in teaching hospitals. Scientific Journal of Kurdistan University of Medical Sciences. 2017;22(4):91-100.

130. Khuwaja AK, Qureshi R, Azam SI. Prevalence and factors associated with anxiety and depression among family practitioners in Karachi, Pakistan. J Pak Med Assoc. 2004;54(2):45-9.

131. Mushtaq M, Sultana S, Imtiaz I. The Trauma of Sexual Harassment and its Mental Health Consequences Among Nurses. J Coll Physicians Surg Pak. 2015;25(9):675-9.

132. Nabi N, Yousuf A, Iqbal A. Prevalence of anxiety and depression among doctors working in a private hospital in Pakistan. ASEAN Journal of Psychiatry. 2012;13(1):13-9.

133. Saifan AR, Al Zoubi AM, Alrimawi I, Melhem O. Exploring the psychological status of Jordanian nurses working with cancer patients. J Nurs Manag. 2019;27(1):215-22.

134. Saquib J, Taleb M, AlMeimar R, Alhomaidan HT, Al-Mohaimeed A, AlMazrou A, et al. Job insecurity, fear of litigation, and mental health among expatriate nurses. Arch Environ Occup Health. 2020;75(3):144-51.

135. Saquib N, Zaghloul MS, Saquib J, Alhomaidan HT, Al-Mohaimeed A, Al-Mazrou A. Association of cumulative job dissatisfaction with depression, anxiety and stress among expatriate nurses in Saudi Arabia. J Nurs Manag. 2019;27(4):740-8.

136. Tomas-Sabado J, Maynegre-Santaularia M, Perez-Bartolome M, Alsina-Rodriguez M, Quinta-Barbero R, Granell-Navas S. [Burnout syndrome and suicide risk among primary care nurses]. Enferm Clin. 2010;20(3):173-8.

137. Zafar W, Khan UR, Siddiqui SA, Jamali S, Razzak JA. Workplace Violence and Self-reported Psychological Health: Coping with Post-traumatic Stress, Mental Distress, and Burnout among Physicians Working in the Emergency Departments Compared to Other Specialties in Pakistan. J Emerg Med. 2016;50(1):167-77 e1.

138. Zaghloul MS, Saquib J, AlMazrou A, Saquib N. Mental Health Status of Expatriate Nurses in Northcentral Saudi Arabia. J Immigr Minor Health. 2019;21(6):1233-40.

139. Zahid MA, Al-Sahlawi KS, Shahid AA, Awadh JA, Abu-Shammah H. Violence against doctors: 2. Effects of violence on doctors working in accident and emergency departments. Eur J Emerg Med. 1999;6(4):305-9.
